# From Ru-bda to Ru-bds: a step forward to highly efficient molecular water oxidation electrocatalysts under acidic and neutral conditions

**DOI:** 10.1038/s41467-020-20637-8

**Published:** 2021-01-14

**Authors:** Jing Yang, Lei Wang, Shaoqi Zhan, Haiyuan Zou, Hong Chen, Mårten S. G. Ahlquist, Lele Duan, Licheng Sun

**Affiliations:** 1 Department of Chemistry, Shenzhen Grubbs Institute and Guangdong Provincial Key Laboratory of Energy Materials for Electric Power, Southern University of Science and Technology, Shenzhen, 518055 People’s Republic of China; 2 Department of Chemistry, School of Engineering Sciences in Chemistry, Biotechnology and Health, KTH Royal Institute of Technology, 10044 Stockholm, Sweden; 3 Department of Theoretical Chemistry & Biology, School of Engineering Sciences in Chemistry, Biotechnology and Health, KTH Royal Institute of Technology, 10044 Stockholm, Sweden; 4 School of Environmental Science & Engineering, Southern University of Science and Technology, Shenzhen, 518055 People’s Republic of China; 5 State Key Laboratory of Fine Chemicals, DUT − KTH Joint Education and Research Center on Molecular Devices, Dalian University of Technology (DUT), Dalian, 116012 People’s Republic of China; 6 Center of Artificial Photosynthesis for Solar Fuels, School of Science, Westlake University, 310024 Hangzhou, China

**Keywords:** Chemistry, Catalysis, Coordination chemistry, Electrochemistry

## Abstract

Significant advances during the past decades in the design and studies of Ru complexes with polypyridine ligands have led to the great development of molecular water oxidation catalysts and understanding on the O−O bond formation mechanisms. Here we report a Ru-based molecular water oxidation catalyst [Ru(bds)(pic)_2_] (**Ru-bds**; bds^2−^ = 2,2′-bipyridine-6,6′-disulfonate) containing a tetradentate, dianionic sulfonate ligand at the equatorial position and two 4-picoline ligands at the axial positions. This **Ru-bds** catalyst electrochemically catalyzes water oxidation with turnover frequencies (TOF) of 160 and 12,900 s^−1^ under acidic and neutral conditions respectively, showing much better performance than the state-of-art **Ru-bda** catalyst. Density functional theory calculations reveal that (i) under acidic conditions, the high valent Ru intermediate Ru^V^=O featuring the 7-coordination configuration is involved in the O−O bond formation step; (ii) under neutral conditions, the seven-coordinate Ru^IV^=O triggers the O−O bond formation; (iii) in both cases, the I2M (interaction of two M−O units) pathway is dominant over the WNA (water nucleophilic attack) pathway.

## Abstract (web summary)

Developing efficient molecular water oxidation catalysts for artificial photosynthesis is a challenging task. Here the authors introduce a ruthenium based complex with negatively charged sulfonate groups to effectively drive water oxidation under both acidic and neutral conditions.

## Introduction

Artificial photosynthesis is one of the most promising ways to build our society with sustainable energy systems while catalysis of water splitting is the key^1^. Two half-reactions are involved in the water-splitting process: water oxidation (2H_2_O → O_2_ + 4H^+^ + 4e^−^) and proton reduction (4H^+^ + 4e^−^ → 2H_2_). The first half reaction is widely regarded as the bottleneck of total water splitting. To date, only Ir-based catalysts display high catalytic activity and long durability towards electrochemical water oxidation under strongly acidic conditions^2,3^. The majority of noble-metal free water oxidation catalysts (WOCs) are confined under strong alkaline conditions whereas they show only moderate performances under neutral conditions and quickly deactivate in acidic media^4–6^. In contrast, molecular WOCs displayed their advantages over the majority of metal oxides with regard to stability and catalytic activity under acidic conditions. In the last decade, a great number of WOCs based on transition metal complexes such as Fe^7–9^, Ir^10^, Co^11,12^, Mn^13,14^, Cu^15,16^ and Ru^17–20^ have been reported, among which Ru-based WOCs with relatively simple polypyridyl ligands are the most representative due to their adequate stability and high catalytic activity under strongly acidic conditions^21–23^.

**Fig. 1:**
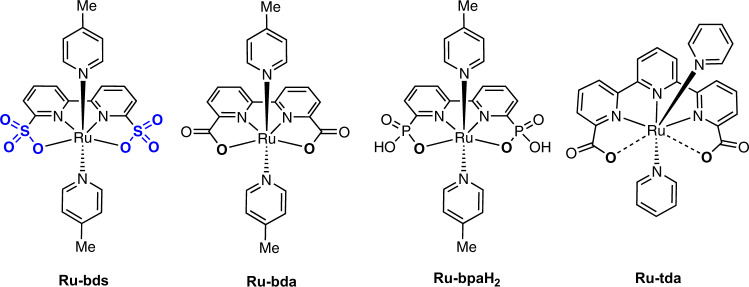
Structure illustration of complexes. Chemical structures of **Ru-bds**, **Ru-bda**, **Ru-bpaH**_**2**_ and **Ru-tda**.

Inspired by the first Ru-based WOC, [(bpy)_2_(H_2_O)Ru^III^ORu^III^(OH_2_)(bpy)_2_](ClO_4_)_4_ (bpy = 2,2′-bipyridine), reported by Meyer et al. in 1982^24^, many other Ru WOCs have been developed recently^25,26^, such as **Ru-Hbpp** (Hbpp^−^ = 3,5-bis(2-pyridyl)pyrazolate)^27^ by Llobet and **Ru-bnp** (bnp = 4-*tert*-Butyl-2,6-di([1′,8′]-naphthyrid-2′-yl)pyridine) by Thummel^28^. The mononuclear Ru catalysts, [Ru(bda)(L)_2_] (**Ru-bda**; H_2_bda = 2,2′-bipyridine-6,6′-dicarboxylic acid; L = N-heterocyclic ligands; Fig. 1), first developed by the Sun group, are among the most efficient molecular WOCs^17,21^. This type of catalysts catalyze the O−O bond formation via the I2M (interaction of two M−O units) pathway. Later, Llobet and co-workers reported a **Ru-tda** WOC (Fig. 1) with catalytic current density of 4.8 mA/cm^2^ at 1.56 V under pH 7.0 conditions^29^. Recently, Concepcion and co-workers modified the bda^2−^ ligand by introducing phosphate groups instead of carboxylate groups and prepared complexes **Ru-bpaH**_**2**_ (Fig. 1) and [Ru(bpHc)(L)_2_] (bpHc^2−^ = 6′-phosphono-[2,2′-bipyridine]-6-carboxylate) with more or less enhanced activity^30–32^. The **Ru-bda** is very efficient with cerium(IV) ammonium nitrate ((NH_4_)_2_[Ce^IV^(NO_3_)_6_]) as a sacrificial chemical oxidant. However, its electocatalytic performance is not among the best reported so far. Previous studies on the **Ru-bda** catalysts revealed that electron-withdrawing groups on the axial ligands could enhance water oxidation activity^33^.

In this work, by changing the carboxylate ligand to a less electron-donating sulfonate ligand, 2,2′-bipyridine-6,6′-disulfonate (bds^2−^), we invent a type of highly efficient water oxidation electrocatalyst, [Ru(bds)(pic)_2_] (**Ru-bds**; pic = 4-picoline; Fig. 1), that reaches turnover frequencies (TOF) of 160 and 12900 s^−1^ at pH 1.0 and 7.0 conditions respectively, which outperforms most reported Ru catalysts under similar conditions^21,29,30^, including the typical **Ru-bda**.

## Results and discussion

### Synthesis and characterization

The ligand H_2_bds was prepared by the nucleophilic substitution of the bromo groups on 6,6′-dibromo-2,2′-bipyridine by NaHS at elevated temperature, followed by the oxidation of the hydrosulfide groups by HNO_3_ to obtain the desired sulfonate containing ligand H_2_bds. Subsequent coordination of the H_2_bds ligand to [Ru(*p*-cymene)Cl_2_]_2_ in the presence of NEt_3_, followed by reaction with 4-picoline yields the corresponding mononuclear catalyst **Ru-bds**. The ^1^H/^13^C NMR and ^1^H-^1^H COSY spectra of H_2_bds and **Ru-bds** were shown in Supplementary Figs. 1–8.

**Fig. 2:**
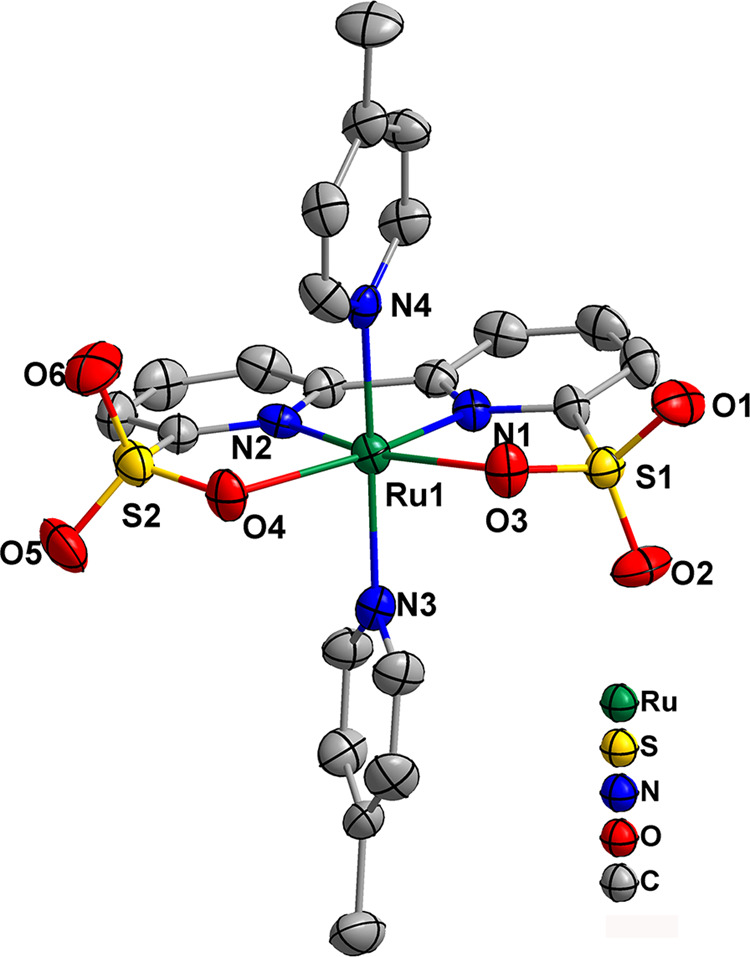
X-ray crystal structure. The X-ray crystal structure of **Ru-bds** with thermal ellipsoids at 50% probability (hydrogen atoms are omitted for clarity).

The **Ru-bds** crystallizes with two chemically identical but crystallographically distinct molecules in the asymmetric unit. One of the X-ray crystal structures was depicted in Fig. 2, and the Ru centre is in a typical distorted octahedral geometry. It is noticeable that the O3−Ru1−O4 bite angle of **Ru-bds** (114.7(3)°) is slightly larger than that of previously reported **Ru**^**III**^**-bpaH**_**2**_ (112.09(6)°)^30^ whereas smaller than that of **Ru**^**II**^**-bda** (123.0(2)°)^17^, all of which are significantly larger than the ideal 90° of an octahedron configuration. The wide O−Ru−O angle provides the open site of the catalyst for substrate water binding and favors the formation of seven-coordinate Ru intermediates during the catalytic process of water oxidation. Noteworthy, as depicted in Supplementary Fig. 9, the C19−C18−C17 angle of 127.1(8)° in **Ru-bds** is a little bit smaller than C8−C7−C6 (129.6(7)°) in **Ru-bda**, showing that the bipyridine ring of **Ru-bds** is less distorted in comparison with that of **Ru-bda**. Thereby, the bds^2−^ ligand backbone fits the Ru^II^ cation slightly better than the bda^2−^ ligand.

The ^1^H NMR spectrum of **Ru-bds** in CD_2_Cl_2_/CD_3_OD (Supplementary Fig. 10a) is in agreement with a *C*_2*v*_ symmetry of its chemical structure. Two doublets at 8.53 and 8.09 ppm and one triplet at 7.98 ppm were assigned to the proton resonances of the equatorial bds^2−^ ligand while two-resonance peaks at 7.82 and 7.03 ppm to two axial 4-picoline ligands. When the mixed CD_3_CN/D_2_O was used as the solvent, four broad proton resonances originating from the equatorial ligand appeared while two doublets of the 4-picoline protons remained but slightly downshifted to 8.00 and 7.19 ppm, pointing to a loss of *C*_2*v*_ symmetry (Supplementary Fig. 10b). This is attributed to the formation of acetonitrile-coordinating complex, [Ru(*k*_*3*_^O,N,N^-bds)(pic)_2_(CH_3_CN)] with a dangling sulfonate arm. Similar dissociation of the equatorial ligand, such as carboxylate group, was found occurring as well for **Ru-bda**^34^ and **Ru-pda** (H_2_pda is 1, 10-phenanthroline-2,9-dicarboxylic acid)^35^. Labile carboxylate chelation is important for water substrate coordinating to the metal center at Ru^III^ states^21^. Thus, the sulfonate chelation in **Ru-bds** should also promote the water coordination to Ru^III^ center and facilitate the further water oxidation steps.

The catalytic performance of this **Ru-bds** under Ce^IV^-driven water oxidation conditions is less effective but more durable than **Ru-bda** (Supplementary Fig. 11). Kinetic measurements revealed that the oxygen evolution is a first order reaction in regard to both catalyst (Supplementary Fig. 12) and Ce^IV^ (Supplementary Fig. 13) concentrations, meaning that the rate-determining step is an oxidation step, likely Ru^IV^−OH to Ru^V^=O. The low activity is due to the small driving force of Ce^IV^ (*E*(Ce^IV/III^) = 1.6 V vs. NHE^36^; all potentials herein are reported versus normal hydrogen electrode, NHE) in comparison with the catalytic onset potential of **Ru-bds** (*E*_onset_ = 1.6 V; *vide infra*). We thereby studied the electrochemical driven water oxidation by **Ru-bds** in the rest of this work.

### Electrochemistry under acidic conditions

**Fig. 3:**
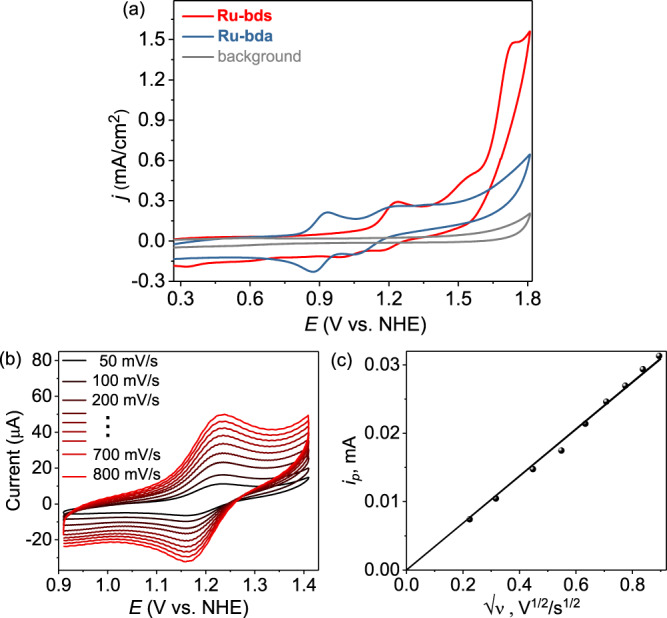
Electrochemical behavior under acidic conditions. **a** CVs of 1.0 mM **Ru-bda** and **Ru-bds** in pH 1.0 triflic acid aqueous solution containing 20% volume CH_3_CN with scan rate of 100 mV s^−1^ (the diameter of glassy carbon working electrode is 3 mm). **b** CV scan rate dependence with 1.0 mM **Ru-bds** from 0.91 to 1.41 V and scan rate varies from 50 to 800 mV s^−1^. **c** Dependence of the peak current for the Ru^III/II^ couple (*E*_1/2_ = 1.18 V) on the square root of scan rate.

First, the electrochemistry of **Ru-bda** and **Ru-bds** was studied in pH 1.0 triflic acid aqueous solutions with 20% CH_3_CN (to improve the solubility of **Ru-bds**; it should be noted that acetonitrile competes with water accessing the Ru center and thereby suppresses water oxidation activity^23^). As shown in Fig. 3a and Supplementary Fig. 14, **Ru-bda** displays three redox waves at 0.89, 1.12 and 1.33 V, which are related to three consecutive metal-based one-electron oxidation processes: Ru^II^−NCCH_3_ → Ru^III^−OH_2_ → Ru^IV^−OH → Ru^V^=O, respectively. A small increase in the current density of roughly 0.6 mA/cm^2^ in the 1.5−1.8 V zone indicates slow catalytic water oxidation. Nevertheless, a substantial enhancement of catalytic current density was observed from the CV curve of **Ru-bds** under the acidic conditions (Fig. 3a) reaching a value of 1.48 mA/cm^2^ at 1.74 V. The consecutive oxidation processes of **Ru-bds** were assigned according to the potential versus pH diagram (Supplementary Fig. 15; *vide infra*). The first redox wave is clearly visible at 1.18 V, attributed to the single electron process of Ru^II^−NCCH_3_ → Ru^III^−OH_2_. The second, irreversible oxidation wave at 1.41 V is corresponding to Ru^III^−OH_2_ → Ru^IV^−OH. Oxidation of Ru^IV^−OH to Ru^V^=O (1.58 V) triggers electrochemical water oxidation. All redox states are about 0.3 V higher relative to **Ru-bda**, which is, as expected, due to the relatively less electron-donating ability of the sulfonate group than the carboxylate group.

**Fig. 4:**
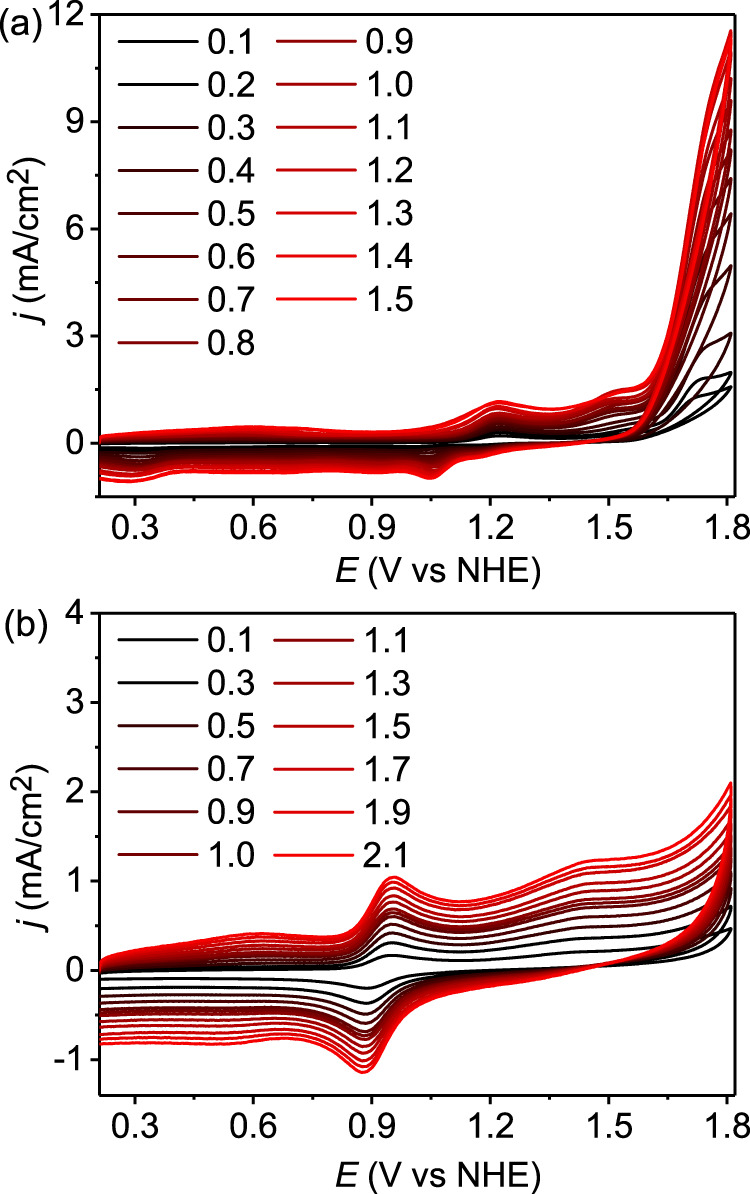
Electrochemistry under acidic conditions. **a** CVs of **Ru-bds** in pH 1.0/CH_3_CN at different scan rates from 0.1 to 1.5 V s^−1^. **b** CVs of **Ru-bda** in pH 1.0/CH_3_CN at different scan rates from 0.1 to 2.1 V s^−1^. Note: *i*_cat_ values at 1.78 V were used to calculate the TOF for both **Ru-bds** and **Ru-bda**.

The Ru^III/II^ redox couples of **Ru-bds** at various scan rates are depicted in Fig. 3b and its peak current (*i*_p_) varies linearly with the square root of scan rate (*ʋ*^1/2^), indicating a diffusion controlled electrochemical process (Fig. 3c). According to the Randles–Sevcik equation (Eq. 1; F is the Faraday constant, A is the electrode area, [Ru^II^] is the bulk concentration of catalyst, n_p_ = 1 is the number of electrons transferred, n_cat_ = 4 is the number of electrons required to complete a single catalytic cycle, D is the diffusion constant of catalyst, R is the ideal gas constant, and T is the temperature), the diffusion co-efficient was 5.02 × 10^−6^ cm^2^s^−1^. CV scans of **Ru-bds** and **Ru-bda** at various scan rates are plotted in Fig. 4a,b. The catalytic currents becomes relatively scan rate independent at 1.5 V s^−1^ for **Ru-bds** and 2.1 V s^−1^ for **Ru-bda**, indicating steady-state conditions are accomplished at such these high scan rates. According to the ratio of *i*_cat_ vs. the noncatalytic Faradaic current (*i*_p_) using an established method with the Eq. 3^37^, the TOF value of **Ru-bds** is calculated to be 160 s^−1^ at a scan rate of 1.5 V s^−1^ toward electrochemical water oxidation whereas **Ru-bda** displays a TOF of 7 s^−1^ at a scan rate of 2.1 V s^−1^. Given the above results, **Ru-bds** is surpassing the well-known mononuclear reference **Ru-bda** catalyst towards electrochemical water oxidation under acidic conditions.

1 $$i_p{\mathrm{ = 0}}{\mathrm{.446n}}_p{\mathrm{FA}}\left[ {{\mathrm{Ru}}^{{\mathrm{II}}}} \right]\left( {{\mathrm{n}}_p{\mathrm{F}}\upsilon D{\mathrm{/RT}}} \right)^{{\mathrm{1/2}}}$$

2 $$i_{{\mathrm{cat}}}{\mathrm{ = n}}_{{\mathrm{cat}}}{\mathrm{FA}}\left[ {{\mathrm{Ru}}^{{\mathrm{II}}}} \right]\left( {{\mathrm{D}}k_{{\mathrm{cat}}}} \right)^{{\mathrm{1/2}}}$$

3 $${\mathrm{TOF = 0}}{\mathrm{.1992}}\left( {\frac{{F\upsilon }}{{{\mathrm{RT}}}}} \right)\left( {\frac{{n_p^3}}{{n_{{\mathrm{cat}}}^2}}} \right)\left( {\frac{{i_{{\mathrm{cat}}}}}{{i_p}}} \right)^2$$

To provide more insights into these redox processes and the electronic effect of the equatorial bds^2−^ ligand on the redox properties, the potential versus pH diagram (Pourbaix diagram) of **Ru-bds** was constructed and shown in Supplementary Fig. 15. The potential of the first oxidation step is constant to be ca. 1.18 V up to pH 3.2. The pH independence indicates that only electron transfer is involved in the first oxidation, corresponding to the oxidation of Ru^II^−NCCH_3_ to 6-coordinate Ru^III^−OH_2_, [Ru^III^(*k*_*3*_^O,N,N^-bds)(pic)_2_(OH_2_)]^+^. Therefore, the p*K*_a_ value of Ru^III^−OH_2_ was determined to be 3.2, which is much smaller than the p*K*_a_ value of 5.5 in **Ru-bda**^21^; this is further in accordance with the relatively poor electron-donating ability of the sulfonate group compared with the carboxylate group. As the pH increases from 3.2 all the way to 8.1, the Ru^III/II^ redox couple decreases linearly as the solution pH increases with of a slope −53 mV per pH, corresponding to typical proton-coupled electron transfer (PCET) process and assigned to the oxidation of Ru^II^−NCCH_3_ to Ru^III^−OH. For the Ru^IV/III^ redox couple, as evidenced by a slope of −64 mV per pH over the range from pH 1.18 to 3.2, the PCET process can be ascribed to the oxidation of Ru^III^−OH_2_ to Ru^IV^−OH. Unfortunately, this redox event became too weak to be distinguished at pH > 3.0 and meanwhile it is overlapped with the catalytic current; thereby, the corresponding potential values were obtained by measuring the potential values at 3.0 × 10^−5^ mA for each scan. The potential of the Ru^IV/III^ is constant to be 1.26 V from pH 3.2 to 5.3, which is related to the process of Ru^III^−OH to Ru^IV^−OH with only electron transfer. As the pH increase from 5.3 to 8.1, there is a PCET process belonging to the oxidation process of Ru^III^−OH to Ru^IV^=O. For the Ru^IV^−OH species produced under acidic conditions, its oxidation via the PCET process leads to the formation of Ru^V^=O, a highly oxidizing state. Additionally, the CV of **Ru-bds** in D_2_O containing 0.1 M triflic acid was also recorded (Supplementary Fig. 18) and the wave of Ru^III^−OH_2_ to Ru^IV^−OH is too weak to be observed, indicating the slow kinetics of Ru^IV^−OH formation and thus its low concentration on the electrode surface.

The chronoamperometric curve of **Ru-bds** renders a moderate decay with 42% retention of the initial current density after 2 h bulk electrolysis in pH 1.0 (Supplementary Fig. 19). The corresponding Faradaic efficiency was recorded to be 95%. The post-electrolyzed CVs (Supplementary Fig. 20) demonstrated a two-third peak current loss for Ru^III/II^ associated with the catalytic current density declined (from 0.77 to 0.41 mA cm^−2^ at 1.7 V). After bulk electrolysis, **Ru-bds** species is still the dominated species, as shown in the HRMS spectra (Supplementary Figs. 21–24), along with the appearance of several new signals with weak intensity, which are presumably structure-evolved Ru-intermediates and probably contributed to the new redox waves in CVs.

### Density functional theory (DFT) calculations

**Fig. 5:**
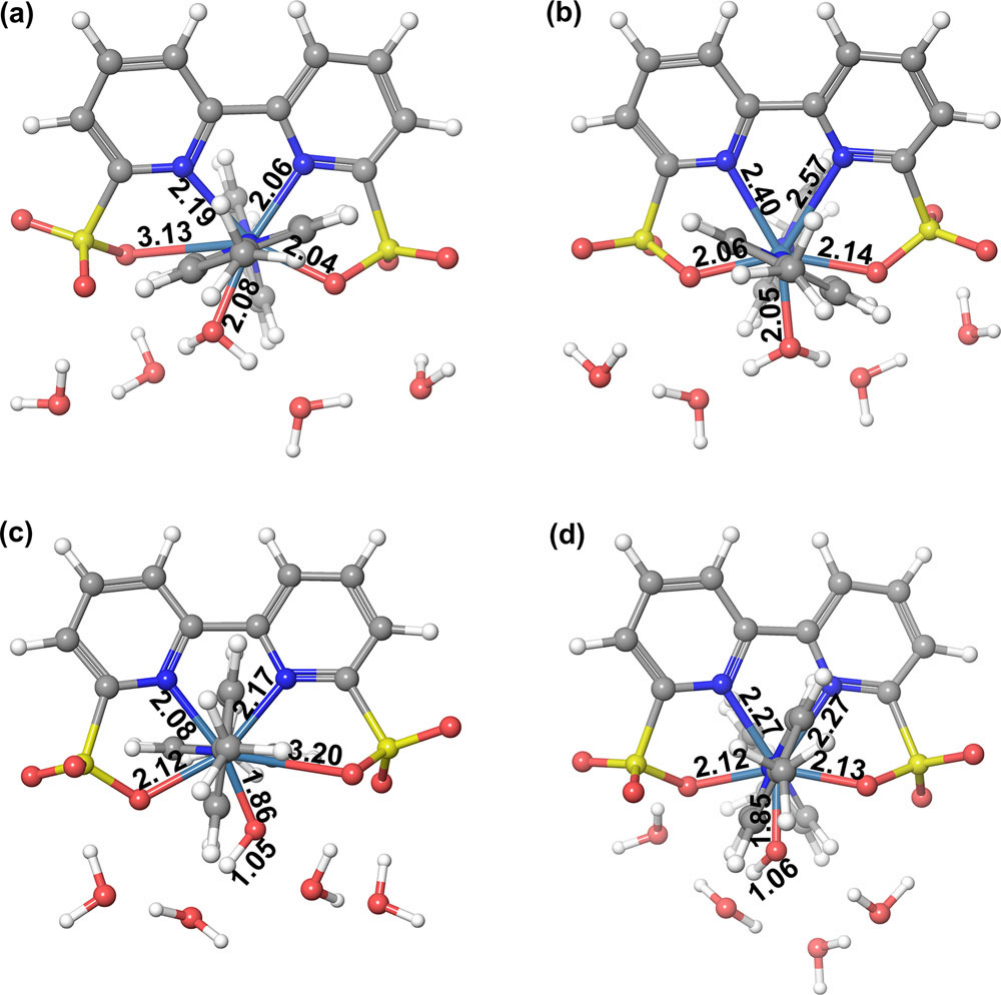
DFT optimized structures. DFT optimized geometries for the structure conversion of [Ru^III^−OH_2_]^+^ between **a** 6-coordination and **b** 7-coordination configurations, and [Ru^IV^−OH]^+^ between **c** 6-coordination and **d** 7-coordination configurations. The bond lengths are given in Å.

DFT calculations were performed to understand the reason on the high electrochemical driven water oxidation performance of **Ru-bds** and the reaction mechanism behind. As described in the previous study^38^, four extra water molecules were added around the metal complex to produce a reasonable hydrogen-bonding network. The calculated potential for the couple [Ru^II^]/[Ru^III^−OH_2_]^+^ at pH 1.0 is 1.15 V, which is close to the experimental value of 1.18 V. Both structures of 6-coordinate [Ru^III^(*k*_*3*_^O,N,N^-bds)(pic)_2_(OH_2_)]^+^ and 7-coordinate [Ru^III^(bds)(pic)_2_(OH_2_)]^+^ were optimized (Fig. 5a, b). The Gibbs free energy of the 6-coordination mode is 6.7 kcal mol^−1^ lower than that of the 7-coordination. The formation of Ru aqua species plays a vital role for water oxidation because it can facilitate further oxidation of the Ru catalyst to higher oxidation states via the PCET process. Upon oxidation of the Ru^III^ aqua species via a PECT process, [Ru^IV^−OH]^+^ is formed. The 7-coordinate [Ru^IV^(bds)(pic)_2_(OH)]^+^ (Fig. 5d) becomes more stable than the corresponding 6-coordinate mode [Ru^IV^(*k*_*3*_^O,N,N^-bds)(pic)_2_(OH)]^+^ (Fig. 5c) by a Gibbs free energy difference of 13.3 kcal mol^−1^. The corresponding redox potential of [Ru^III^−OH_2_]^+^_7-coordinate_/[Ru^IV^−OH]^+^_7-coordinate_ is 1.56 V, which is 0.29 V lower than that of 6-coordinate [Ru^III^−OH_2_]^+^. Further oxidation of [Ru^IV^−OH]^+^ leads to the formation of [Ru^V^=O]^+^ with a calculated oxidation potential of 1.85 V at pH 1.0, compared to the experimental value of 1.58 V.

**Fig. 6:**
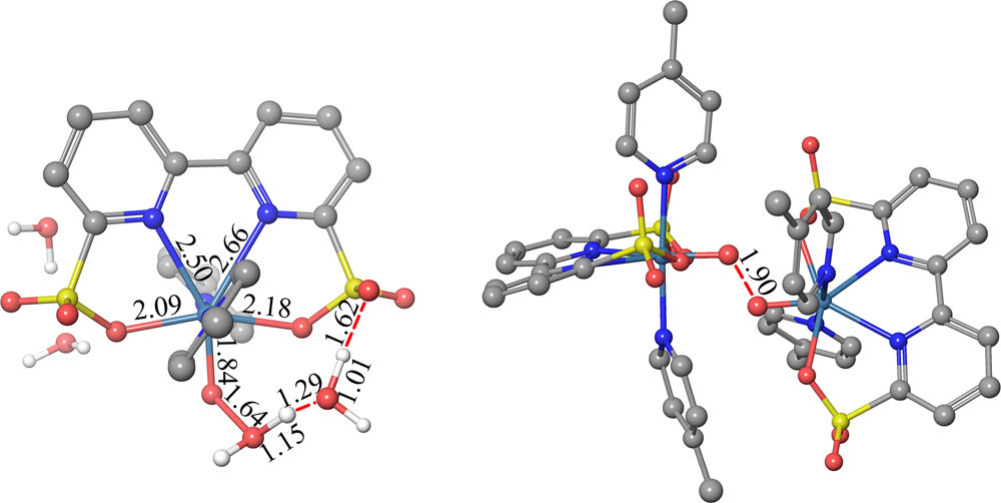
DFT optimized structures. The transition state structures of WNA (left) and I2M (right) pathway. H atoms except those bonding in water molecules are omitted for clarity. The bond lengths are given in Å.

After the formation of [Ru^V^=O]^+^, the O−O bond formation step was calculated via both WNA and I2M pathways (Supplementary Fig. 25) and their corresponding transition states are shown in Fig. 6. The computed activation free energy of the reaction via the WNA pathway is 15.5 kcal mol^−1^ and the reaction free energy is −3.6 kcal mol^−1^. For the I2M pathway, the full reaction includes diffusion of two [Ru^V^=O]^+^ species, encountering of two [Ru^V^=O]^+^ species to form the prereactive dimer and the radical coupling reaction in the full solvent^39–41^. The computed activation free energy for the radical coupling step is 6.9 kcal mol^−1^ from the prereactive dimer to the transition state, and the reaction free energy is −11.5 kcal mol^−1^. The encoutering step of two species was found to have a key role for the different catalytic reactivities of **Ru-bda** complexes. We therefore parameterized [Ru^V^=O]^+^ model according to our published report^41^ and tested stability of the model (Supplementary Fig. 26). Based on the analysis of the hydrogen bonding (Supplementary Fig. 27), the oxo of [Ru^V^=O]^+^ of **Ru-bds** is found to be hydrophobic, similar to the oxo of **Ru-bda**. We performed potential of mean force (PMF) simulations to estimate the binding free energy of the prereactive dimer in water phase. The calculated binding free energy of the two [Ru^V^=O]^+^ species in water phase is −4 kcal mol^−1^ (Supplementary Fig. 28), leading to a total activation energy of 2.6 kcal mol^−1^ for **Ru-bds** (the forward activation free energy of the O−O bond formation minus the free energy of the dissociation of two species; Supplementary Table 2) for the full dimerization. Apparently, the I2M pathway is feasible for **Ru-bds**. In comparison, the activation energy of **Ru-bds** is slightly lower than that of **Ru-bda** (3.9 kcal mol^−1^)^40^.

### Proposed mechanism

**Fig. 7:**
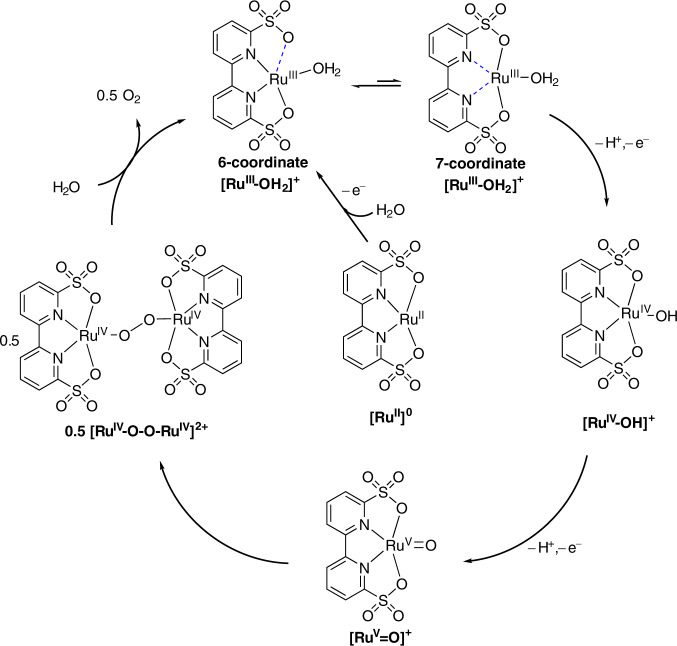
Proposed mechanism. Proposed electrochemical driven water oxidation and O−O bond formation pathway by **Ru-bds** under the acidic conditions.

On the basis of the above electrochemistry, mass spectrometry, kinetics studies together with DFT calculations, the reaction mechanism of water oxidation by **Ru-bds** under acidic conditions is proposed and depicted in Fig. 7^42^.

### Base-enhanced catalytic water oxidation

As shown previously by Meyer and co-workers for the base-enhanced PECT oxidation, the role of the added buffer base was explored by CVs of **Ru-bds** (1.0 mM) at pH 7.0 while maintaining the ionic strength at 0.5 M (compensated by NaNO_3_, Supplementary Fig. 31). Significant acceleration of catalytic current is achieved toward water oxidation as the total concentration of H_2_PO_4_^−^/HPO_4_^2−^ buffers increases gradually from 0.01 M to 0.2 M, during which a wave with nearly plateau shape was observed at the lowest buffer concentration where a maximum current reached at 1.28 V. Therefore, the involvement of the buffer base in PCET pathway directly contributes to the enhanced catalytic activity, which is associated with the electron transferring to electrode occurring along with proton transfer to the added base. CVs were also recorded with different catalyst concentrations at pH 7.0. As the concentration varies from 0.2 mM to 1.0 mM, a sharp enhancement of catalytic current for water oxidation was obtained (Supplementary Fig. 32).

### Electrochemistry under neutral conditions

**Fig. 8:**
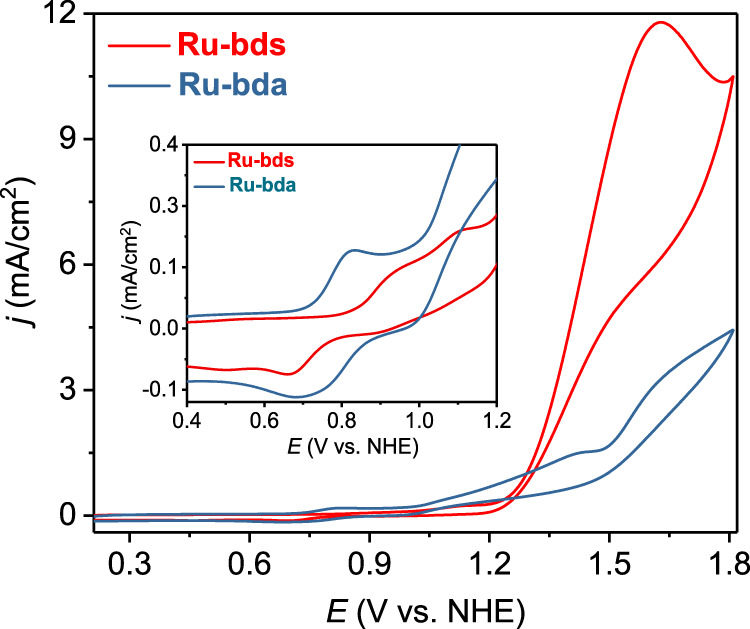
Electrochemical behavior under neutral conditions. CVs of 1.0 mM **Ru-bda** and **Ru-bds** in pH 7.0 phosphate buffer containing 20% volume CH_3_CN with scan rate of 100 mV s^−1^ [0.2 M phosphate buffer, I = 0.5 M (NaNO_3_)], the diameter of glassy carbon working electrode is 3 mm. Inset: Enlargement of the 0.4−1.2 V zone in the CV.

**Fig. 9:**
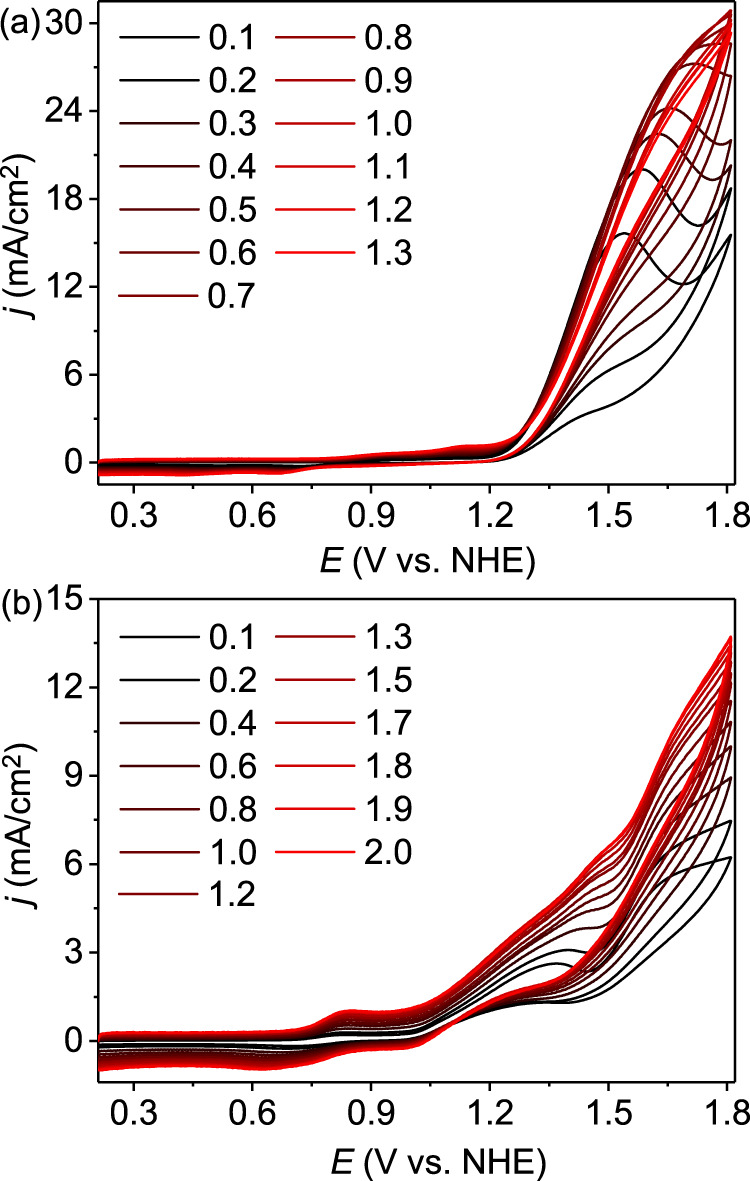
Electrochemistry under neutral conditions. **a** CVs of **Ru-bds** in pH 7.0 phosphate buffer containing 20% CH_3_CN at different scan rates from 0.1 to 1.3 V s^−1^. **b** CVs of **Ru-bda** in pH 7.0/CH_3_CN at different scan rates from 0.1 to 2.0 V s^−1^. Conditions: 0.2 M phosphate buffer, I = 0.5 M (NaNO_3_), glassy carbon working electrode (*Φ* 3 mm). Note: *i*_cat_ values at 1.70 V were used to calculate the TOF for both **Ru-bds** and **Ru-bda**.

Accordingly, electrochemical behaviors of 1.0 mM **Ru-bds** and **Ru-bda** were further explored in 0.20 M pH 7.0 phosphate buffer solutions (Fig. 8 and Supplementary Fig. 33). To our delight, **Ru-bds** exhibited an ultra-high electrocatalytic current density of 11.79 mA cm^−2^ (at 1.63 V with scan rate of 0.1 mV s^−1^, Fig. 8) with a low onset overpotential of 380 mV. In comparison, the current density of **Ru-bda** is only 3.26 mA cm^−2^ (at 1.63 V with scan rate of 0.1 mV s^−1^, Fig. 8). This significant current density enhancement by **Ru-bds** highlights the importance of PCET in avoiding the high energy barrier for the electrochemical water oxidation. To the best of our knowledge, such a value of the catalytic current density is among the highest ever reported in the literature under neutral conditions for Ru-WOCs catalysed water oxidation (Supplementary Table 3)^29,30,43,44^. Under the steady-state conditions, a TOF of 12900 s^−1^ was achieved by **Ru-bds** at the scan rate of 1.3 V s^−1^ (Fig. 9a) while **Ru-bda** displayed a TOF of 300 s^−1^ at the scan rate of 2.0 V s^−1^. The latter is about two orders of magnitude smaller than that of **Ru-bds** (Fig. 9b). Bulk electrolysis of **Ru-bds** in pH 7.0 for 2 h displays a decay with 30% retention of the initial current density (Supplementary Fig. 34a) while the Faradaic efficiency was determined to be 94%. The CVs (Supplementary Fig. 35) show that the Ru^III/II^ peak current retains after bulk electrolysis while the catalytic current density of 8.2 mA cm^−2^ only decays to 6.8 mA cm^−2^. The two signals of [Ru^II^(bds^2−^)(pic)_2_ + Na]^+^ and [Ru^II^(bdsNa^−^)(pic)_2_(CH_3_CN)]^+^ are still the dominate species without formation of new Ru species (Supplementary Figs. 36 and 37).

In our previous study, the mixed-valence Ru trimer species for **Ru-bda**-catalyzed water oxidation under neutral conditions were proved by using chemical and electrochemical methods and have been successfully isolated and characterized^45^. Herein, the spectroelectrochemistry of **Ru-bds** was examined at a constant potential of 1.21 V, where the absorption changes of the electrolysis solution were in situ monitored by UV-Vis spectroscopy. As shown in Supplementary Fig. 38, no observable band at 690 nm was observed where the band at 690 nm is a typical absorption peak of the trinuclear Ru species. Therefore, there is no *µ*-oxo oligomeric species formed in the case of **Ru-bds**. More importantly, the electrode surfaces before and after electrolysis have also been characterized by scanning electron microscopy (SEM) and energy-dispersive X-ray spectroscopy (EDX) (Supplementary Fig. 39). No Ru elements and species was deposited on the electrode surface, ruling out the potential catalyst decomposition into the RuO_2_.

As shown in the Pourbaix diagram, a pH independent oxidation process at 1.0 V appears at pH > 5.5 (grey stars in Supplementary Fig. 15), and this oxidation wave is relatively small compared with the Ru^III/II^ wave (Supplementary Fig. 33). The Ru^III/II^ and Ru^IV/III^ data points respectively aligned well in the whole pH range. Thereby, we propose that the pH independent oxidation process at 1.0 V belongs to a decomposed/evolved Ru species whose structure is currently unknown. Given that, the oxidation of Ru^III^−OH to Ru^IV^=O triggers water oxidation with *E*_onset_ = 1.2 V at pH 7.0.

### Density functional theory calculations at pH 7.0

**Fig. 10:**
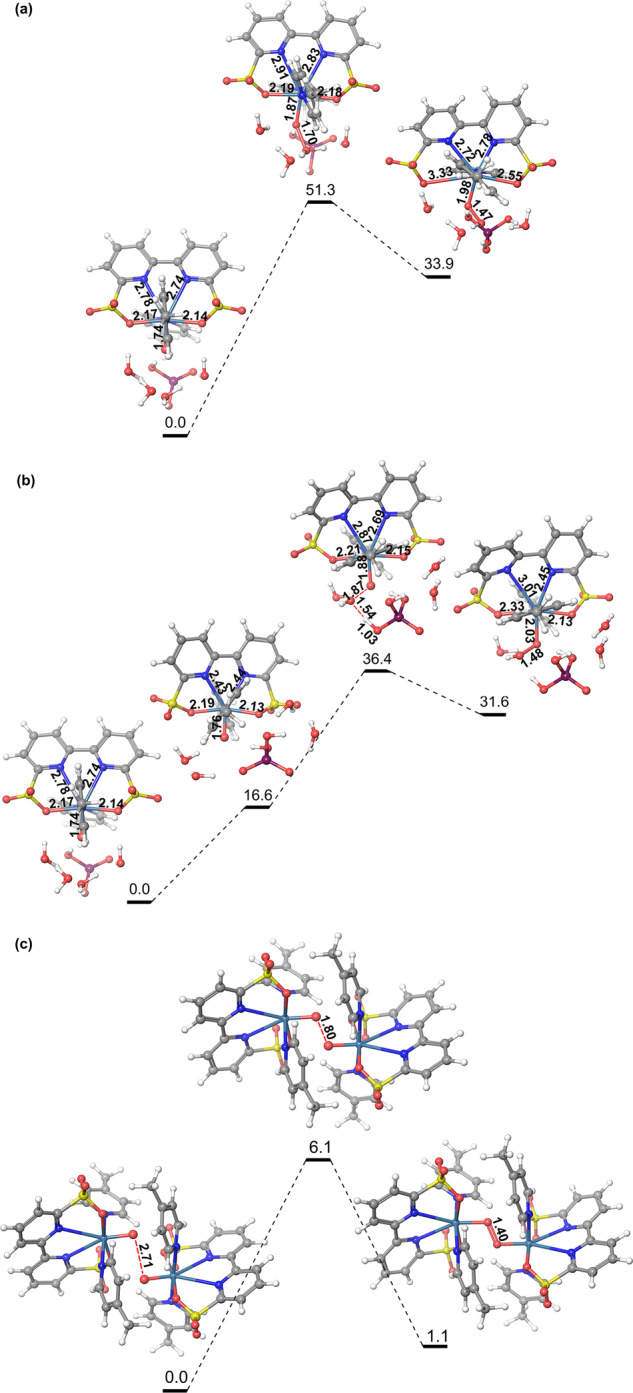
DFT calculated energy profiles. The energy profiles of O−O bond formation of Ru^IV^=O at pH 7.0 with the **a** WNA of triplet Ru^IV^=O using H_2_PO_4_^−^ as base, **b** WNA of singlet Ru^IV^=O using H_2_PO_4_^−^ as base, and **c** I2M mechanism. The units of energies are kcal mol^−1^.

DFT calculations were also performed to gain more insight in the reaction mechanism by **Ru-bds** at pH 7.0. From the Pourbaix diagram, the Ru^III^−OH will form the Ru^IV^=O species at pH > 4.6. Calculations showed that the Gibbs free energy of [Ru^III^−OH]^+^_(6-coordinate)_ is only 0.8 kcal mol^−1^ lower than that of the [Ru^III^−OH]^+^_(7-coordinate)_. In the phosphate buffer, [Ru^IV^−OH]^+^ is deprotonated by H_2_PO_4_^−^ (dominate species in pH 7.0 phosphate buffer), forming the Ru^IV^=O (Supplementary Fig. 40) species. Since the Gibbs free energy of Ru^IV^=O at triplet state is 16.6 kcal mol^−1^ lower than that of singlet geometry, we used triplet state of the Ru^IV^=O species for the calculations. The calculated redox potential of [Ru^III^−OH]^+^_(7-coordinate)_/[Ru^IV^=O]_T_ is 1.08 V at pH 7.0. When the Ru^IV^=O species form the O−O bond via the WNA pathway using H_2_PO_4_^−^ (a stronger base than water) as a proton acceptor, (i) at the triplet state the activation free energy is 51.3 kcal mol^−1^ and the reaction free energy is 33.9 kcal mol^−1^ (Fig. 10a), and (ii) at the singlet state the activation free energy is 19.8 kcal mol^−1^ and the reaction free energy is 15.0 kcal mol^−1^. Therefore the total activation free energy of WNA pathway is 36.4 kcal mol^−1^ and the reaction free energy is 31.6 kcal mol^−1^ (the energy profile at singlet plus the energy difference of 16.6 kcal mol^−1^ between the singlet and triplet of Ru^IV^=O species; Fig. 10b).

We have also calculated transition state of radical coupling of two Ru^IV^=O species by scanning the terminal O−O bond distance [Ru^IV^=O ∙ ∙ ∙ O=Ru^IV^] of the antiferromagnetic open-shell singlet, giving an activation free energy of 6.1 kcal mol^−1^ from the pre-reactive dimer to the transition state, and a reaction free energy of 1.1 kcal mol^−1^ (Fig. 10c). We have also parameterized Ru^IV^=O_T_ and tested stability by molecular dynamics (MD; Supplementary Fig. 26). The two neutral Ru^IV^=O_T_ species are more prone to form the prereactive complex with a lower binding free energy of −5 kcal mol^−1^ (Supplementary Fig. 28), compared to the two [Ru^V^=O]^+^ species. Therefore the overall activation energy of the O−O bond formation from two separated Ru^IV^=O_T_ complexes is only 1.1 kcal mol^−1^. Further oxidation of [Ru^IV^=O]_T_ to form the [Ru^V^=O]^+^ however has an oxidation potential of 1.89 V. The high oxidation potential of Ru^V/IV^ and the high activation free energy of the WNA pathway indicates that under neutral conditions the O−O bond formation would occur via the I2M pathway at the Ru^IV^=O state; the I2M pathway at the Ru^IV^=O state is a new discovery for the molecular based water oxidation catalysis while Meyer and coworkers have reported a Ru^IV^=O catalyzed WNA pathway^46^. Taking the electrochemistry results and theoretical calculations into account, we propose that the rate-limiting step in electrochemical water oxidation by **Ru-bds** at pH 7.0 is the oxidation of Ru^III^−OH to Ru^IV^=O, and the O−O bond forms via the radical coupling of two Ru^IV^=O species.

In summary, by introducing two sulfonate groups into a bipyridine ligand, a mononuclear Ru catalyst **Ru-bds** was developed for effective electrocatalytic water oxidation with TOF of 160 s^−1^ under acidic conditions while outstanding catalytic activity with a high TOF of 12900 s^−1^ was successfully obtained under neutral conditions. The bds^2−^ ligand, with the proper electron-donating ability and suitable ligation sites, plays a vital role on stabilizing high valent Ru states, adapting the 7-coordination configuration of reaction intermediates and promoting the O−O bond formation via the I2M mechanism. DFT calculations revealed that the radical coupling of two Ru^IV^=O species at neutral conditions proceeds with a low activation barrier of 1.1 kcal mol^−1^, providing a new insight in water oxidation mechanism catalyzed by Ru-based water oxidation catalysts under neutral conditions. In particular, this work illuminates the impact of structural changes on the electrochemical catalytic activity and further provides an inspiring strategy to design more efficient and robust WOCs for potential applications in electrocatalysis and photoelectrocatalysis.

## Methods

### Physical measurements and instrumentation

Nuclear magnetic resonance (NMR) spectra were obtained with 400 MHz of Bruker Advance spectrometer. Elemental analyses were performed with an Elementar Vario EL elemental analyzer. Mass spectrometry measurements were carried out by a Thermo Scientific LCQ Fleet mass spectrometer. High-resolution mass spectrometry measurements were performed on a Thermo Scientific Q Exactive mass spectrometer. Electrochemistry measurements were performed with a CHI760 electrochemical workstation. For cyclic voltammetry and differential pulse voltammetry measurements, a glassy carbon disk (diameter 3 mm) was used as the working electrode and a platinum column as the counter electrode, and measured versus Ag/AgCl reference electrode (3 M KCl; 210 mV vs. NHE) in aqueous solutions (conditions: [cat] = 1.0 mM; pH 1.0 triflic acid or pH 7.0, 0.2 M phosphate buffer, I = 0.5 M (NaNO_3_)). For bulk electrolysis, the working electrode was a glassy carbon piece (2.86 cm^2^), the counter electrode a platinum wire and the reference electrode a saturated Ag/AgCl electrode (3 M KCl). Typically, 7 mL of 0.5 mM **Ru-bds** in CH_3_CN/pH 1.0 or pH 7.0 (0.2 M phosphate buffer) solutions (1/4, v/v) was prepared and the electrolysis current was recorded with an applied potential of 1.7 V at pH 1.0 or 1.5 V at pH 7.0. The electrolytic oxygen evolution in a standard three-electrode H-cell configuration was monitored by GC9790Plus. The Ce^IV^-driven oxygen was detected by a pressure transducer (MIK-P300) driven at 10.00 V using a power supply (HY3005B) plus a data acquisition module (Omega OM-DAQ-USB-2401). The single crystal X-ray diffraction data were collected at 298 K on a Bruker SMART CCD 1 K diffractometer with a graphite-monochromated Mo-*K*_α_ radiation (λ = 0.71073 Å). The structure was solved and refined using the Olex2 program^47^. Small fraction of twinning domains was suspected in the present datasets. To understand the twining problem, the twin rotation law has been carefully studied with the Platon software (A.L.Spek, Acta Cryst. 2020, E76, 1–11). The twin law of (−1 0 0 0 0 −1 −0.36 −0.391 1) has been added into the instruction file for the final refinement. The final refinement has been successfully lower down the refinement R1 value from 8.05 % to 6.71 % with improved residue electron density. A twining fraction of 4.5 % as founded based on the refinement results. The spectroelectrochemistry was carried out in a Y-shaped quartz cuvette equipped with Pt mesh as working electrode, a Pt wire as counter electrode and an Ag/AgCl reference electrode. The quartz cuvette was filled with 1 mL 0.5 mM **Ru-bds** in phosphate buffer solution (pH 7, 0.2 M, 20% CH_3_CN). The path length of the Y-shaped quartz cuvette is 1.0 mM. UV−vis absorption spectra were obtained by Agilent Technologies Cary 8454. The morphologies of electrode surfaces before and after electrolysis were characterized by a JSM-7500F field emission scanning electron microscopy (SEM, JEOL, Japan).

### Synthesis of 2,2′-bipyridine-6,6′-disulfonic acid

To a solution of 6,6′-dibromo-2,2′-bipyridine (312 mg, 1.0 mmol) in dry DMF (8 mL), ten equivalents of sodium hydrosulfide (560 mg, 10 mmol) were added. The reaction mixture was heated under 180 °C for 2 h using Microwave reactor. The resultant orange mixture was dried under vacuum. Dissolve the residue in distilled water, and CH_3_COOH was added drop wised until the precipitation stopped. The precipitate was filtrated and washed by distilled water, finally dried under vacuum. The product of 6,6′-dithiol-2,2′-bipyridine was afforded as an orange powder (167 mg, 76% yield; its 1D and 2D NMR spectra were recorded to characterize this compound, as shown in Supplementary Figs. 1–2; ^1^H NMR (400 MHz, *d*_6_-DMSO) δ 13.67 (s, 2H), 7.47 (dd, *J* = 8.5, 7.3 Hz, 2H), 7.36 (dd, *J* = 8.7, 0.9 Hz, 2H), 7.03 (d, *J* = 6.3 Hz, 2H). HRMS: m/z^−^ = 219.0051 (M − H^+^); calcd, 219.0056.), and used for the next step synthesis without further purification. Compound 6,6′-dithiol-2,2′-bipyridine (100 mg) was dissolved in 8 mL nitric acid (70%, purified by redistillation, ≥99.999% trace metals basis), and the solution was heated at 100 °C for 2 h, and then the acid was removed by vacuum. The product of 2,2′-bipyridine-6,6′-disulfonic acid (bdsH_2_) was obtained as pale yellow powder in a quantitative yield. Its 1D and 2D NMR spectra were recorded (Supplementary Figs. 3–5). ^1^H NMR (400 MHz, D_2_O) δ 8.50 (dd, *J* = 8.0, 0.8 Hz, 2H), 8.20 (t, *J* = 7.9 Hz, 2H), 8.02 (dd, *J* = 7.8, 0.7 Hz, 2H). ^13^C NMR (101 MHz, D_2_O) δ 161.50, 157.37, 143.05, 127.28, 124.18. HRMS: m/z^−^ = 314.9750 (M − H^+^); calcd, 315.9751. Calcd. for bdsH_2_ (C_10_H_8_N_2_O_6_S_2_): C 35.93; H 2.55; N 8.86. Found: C 35.81; H 2.99; N 8.83.

### Synthesis of [Ru(bds)(pic)_2_]

To a solution of 2,2′-bipyridine-6,6′-disulfonic acid (100 mg, 0.32 mmol) in dry MeOH (5 mL), dichloro(*p*-cymene)ruthenium(II) dimer (98 mg, 0.16 mmol), ten equivalents of 4-picoline and ten equivalents of Et_3_N were added. The resulting mixture was then heated under 125 °C for 40 min using a microwave reactor. The solvent was removed by vacuum, and the crude product purified by column chromatography. The target complex was afforded as a dark red powder (65 mg, 35% yield). The 1D and 2D NMR spectra (Supplementary Figs. 6–8) were recorded to characterize the complex. ^1^H NMR (400 MHz, mixed CD_3_OD and CD_2_Cl_2_): δ 8.46 (d, *J* = 8.1 Hz, 2H), 8.09 (d, *J* = 7.5 Hz, 2H), 7.96 (t, *J* = 8.0 Hz, 2H), 7.82 (d, *J* = 6.6 Hz, 4H), 7.03 (dd, *J* = 6.6, 0.6 Hz, 4H), 2.31 (s, 6H). ^13^C NMR (101 MHz, *d*_6_-DMSO) δ 163.56, 159.57, 151.11, 148.83, 133.94, 126.01, 125.74, 122.68, 20.23. HRMS: calcd for 602.9973 (M + H^+^); found m/z^+^ = 602.9940. Anal. Calcd for [Ru(bds)(pic)_2_](C_22_H_20_N_4_O_6_RuS_2_): Calcd. C 43.92; H 3.35; N 9.31. Found: C 43.86; H 3.52; N 9.38. Single crystals of **Ru-bds** was obtained by diffusing diethyl ether into CH_3_OH/CH_2_Cl_2_ (1/5, v/v) solution of complex **Ru-bds**.

### Computational details

All DFT calculations for the estimation of Gibbs free energies were carried out with the Jaguar 8.3 program package by Schrödinger LLC^48^. Molecular geometries were optimized using Becke’s three-parameter hybrid functional and the LYP correlation functional (B3LYP)^49^ with D3 correction of Grimme et al^50,51^. with the LACVP** basis set^52^. Single-point energy corrections were performed with the B3LYP-D3 functional using the LACVP** + + basis set augmented with two f-functions on the metal. Frequency calculations were performed on the optimized geometries to verify that the geometries correspond to minima on the potential energy surface (PES). On the basis of the gas-phase optimized geometries, the solvation energies were estimated by single-point calculations using the Poisson−Boltzmann reactive field implemented in Jaguar (PBF) in water. To identify the transition states for O−O bond formation of I2M mechanism, we searched the potential energy surface by scanning the terminal O−O bond distance [Ru^V^=O ∙ ∙ ∙ O=Ru^V^]^2+^ and [Ru^IV^=O ∙ ∙ ∙ O=Ru^IV^] of the antiferromagnetic open shell singlet. The Gibbs free energy were defined as the following equation *G* = *E*(B3LYP-D3/LACVP** + + 2 f on Ru) + G_solv_ + ZPE + *H*_298_ - *TS*_298_ + 1.9 kcal/mol (the value 1.9 kcal/mol is a concentration correction to the free energy of solvation, which by default is calculated at 1 M (g) to 1 M (aq) in Jaguar). Four additional water molecules were included in the calculation.

### Force field parameterization

With the optimized geometries of [Ru^V^=O]^+^ and Ru^IV^=O_T_, equilibrium bonds, angles, and dihedrals were obtained. Then a series of relaxed coordinate scans were performed by DFT calculations from the equilibrium bonds, angles, and dihedrals to get the force field parameters. Each bond was stretched 0.3 Å in each direction for a total scan span of 0.6 Å, and each angle was stretched 15^o^ in each direction for a total scan span of 30^o^. The scans were made in 15 increments each way. The scans were fitted to a second-degree polynomial as the following equation where the square term coefficient, *k*_*b*_, is the force constant.

$$K_b\left( {r - r_0} \right)^2 = K_br^2 - 2K_brr_0 + K_br_0^2$$

The angle parameters have also been plotted with the force constants *vs* equilibrium angle. Because of the fairly planar structure, most of the dihedral angles have been ignored *i.e K*_*ψ*_ = 0, but the dihedral angles that rotates the axial ligand Ru-N bonds have been scanned with the DFT method, and crudely fitted with one cosine series in order to capture the barrier height and equilibrium angles.

Electrostatic potential (ESP) charges were calculated using the DFT method for [Ru^V^=O]^+^ (total charge = +1) and Ru^IV^=O_T_ (total charge = 0). As an improvement on performing only a single ESP charge calculation, a charge-averaging scheme was implemented, which was performed as follows: 1) Initial ESP charges were calculated at the starting geometry. 2) An MD simulation (gas phase, 300 K, step size 1 fs) was performed starting with the initial structure and charges. A harmonic restraint of 5.0 kcal mol^−1^ was added to Ru atom to restrain it from moving too far from its initial position while the other atoms could move freely during 100 ps of simulation time. A snapshot was extracted every 10 ps of this simulation, at which point new ESP charges were calculated. 3) The procedure was repeated three times, but with the exception that the initial charges of the second and third runs were taken as the average of the ESP charges from the previous run. Each time this procedure was repeated, the charges became more stable, and the procedure was stopped on the third round, when the charges had converged (small standard deviations between the partial charges of the different conformations). All van der Waals parameters used in this work are from the work of Bernardes *et. al*.^53^, where the vdW parameters were determined for M(CO)_n_ structures for a number for transition metals.

Since [Ru^V^=O]^+^ and Ru^IV^=O_T_ have the close to identical geometries with different oxidation states, we use same vdW parameters, bond stretch parameters, angle bend parameters, torsion parameters and improper torsion parameters for these two complexes with different calculated average electrostatic potential charges. In order to run MD simulations, the parameters were then transformed into the Gromacs topology format, where the Fourier coefficients of the dihedral potential term were transformed into the Ryckaerd-Bellemans type^54^.

### Molecular dynamics simulations

For two MD simulations, the catalyst was sequentially equilibrated in the following order: 1) A 10 ps simulation at 1 K, applying a 5.0 kcal mol^−1^ harmonic restraint on each atom to restraint them to their DFT optimized geometries, and using a 0.1 fs stepsize. 2) A 100 ps simulation at 100 K, applying a 5.0 kcal mol^−1^ harmonic restraint on each atom and increasing to a 1 fs step size. 3) A final 1 ns equilibration at 300 K, reducing the overall harmonic restraint on each atom to 1 kcal mol^−1^ and remaining the stepsize of 1 fs. Solvent was included in the simulations using a 20 Å radius sphere of explicit TIP3P water molecules centered on the centroid of the reacting atoms. The sphere was described as a multi-level system in which the inner 85% of the sphere was allowed to move freely, but all atoms in the last 25% of the sphere were subjected to a 10 kcal mol^−1^ Å^−2^ to maintain system stability, as is routine when using the Surface Constrained All Atom Solvent (SCAAS) model^55,56^.

### Potential of mean force

PMF were performed for the Ru-Ru distance of two complexes in water phase using umbrella sampling method with the Gromacs 5.1.4 MD software package^57^. Equilibrations were performed for 100 ps under an NVT ensemble, using the same methodology described in previous report^39^. The two Ru complexes were pulled apart for total 10 Å from the core structure over 200 ps, using a spring constant of 30 kJ mol^−1^ Å^−2^ and a pull rate of 0.05 Å ps^−1^. From these trajectories, snapshots were taken to generate the starting configurations for the umbrella sampling windows. Then we sampled the distances using roughly 0.5 Å spacing. Such spacing allowed for increasing detail at smaller Ru-Ru distance, and resulted in 21 windows. In each window, 20 ns of MD was performed for a total simulation time of 420 ns utilized for umbrella sampling. Analysis of results was performed with the weighted histogram analysis method (WHAM)^58^. All PMF simulations resulted in smooth dissociation curves and the mean value from three repeated simulations.

## Supplementary information

Supplementary Information

## Data Availability

All data needed to evaluate the conclusions of this paper are present in the paper and/or Supplementary Information. The source data underlying Supplementary Figs. 26−29 are provided as a Source Data file. The data that support the findings of this study are available from the corresponding author upon request. The X-ray crystallographic data of **Ru-bds** reported in this study has been deposited at the Cambridge Crystallographic Data Center (CCDC), under deposition numbers 1899698 [10.5517/ccdc.csd.cc21rsl6]. The data can be obtained free of charge from The Cambridge Crystallographic Data Center via http://www.ccdc.cam.ac.uk/ data_request/cif. Source data are provided with this paper.

## Source data

Source Data
