# Supplementary material for: From Ru-bda to Ru-bds: a step forward to highly efficient molecular water oxidation electrocatalysts under acidic and neutral conditions
Source: Nat Commun. 2021 Jan 14;12:373. doi: 10.1038/s41467-020-20637-8 (PMC7809030; doi:10.1038/s41467-020-20637-8)
Supplement: Supplementary file 1 — Supplementary Information [file 41467_2020_20637_MOESM1_ESM.pdf]

Supplementary Information for

From Ru-bda to Ru-bds: A Step Forward to Highly Efficient Molecular Water Oxidation Electrocatalysts under Acidic and Neutral Conditions

Jing Yang,[1,¥] Lei Wang,[2,¥] Shaoqi Zhan,[3,¥] Haiyuan Zou,[1] Hong Chen,[4] Mårten S. G. Ahlquist,[3] Lele Duan,*[,1,5] & Licheng Sun*[,2,5,6]

[1]Department of Chemistry, Shenzhen Grubbs Institute and Guangdong Provincial Key Laboratory of Energy Materials for Electric Power, Southern University of Science and Technology, Shenzhen, 518055, P. R. China.

[2]Department of Chemistry, School of Engineering Sciences in Chemistry, Biotechnology and Health, KTH Royal Institute of Technology, 10044 Stockholm, Sweden.

[3]Department of Theoretical Chemistry & Biology, School of Engineering Sciences in Chemistry, Biotechnology and Health, KTH Royal Institute of Technology, 10044 Stockholm, Sweden.

[4]School of Environmental Science & Engineering, Southern University of Science and Technology (SUSTech), Shenzhen, 518055, P. R. China.

[5]State Key Laboratory of Fine Chemicals, DUT−KTH Joint Education and Research Center on Molecular Devices, Dalian University of Technology (DUT), Dalian 116012, P. R. China.

[6]Center of Artificial Photosynthesis for Solar Fuels, School of Science, Westlake University, 310024 Hangzhou, China

[¥] These authors contributed equally: Jing Yang, Lei Wang, Shaoqi Zhan.

*E-mail: duanll@sustech.edu.cn (L. Duan)

*E-mail: lichengs@kth.se (L. Sun)

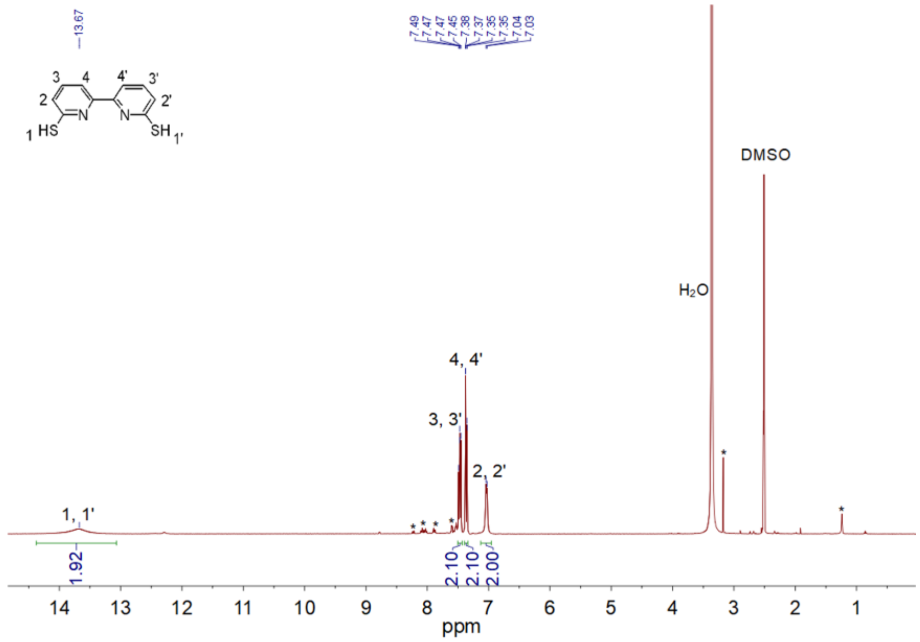

Supplementary Figure 1. ^{1}H NMR spectrum (400 MHz) of 6,6′-dithiol-2,2′-bipyridine in d_6-DMSO (the asterisks indicate impurities).

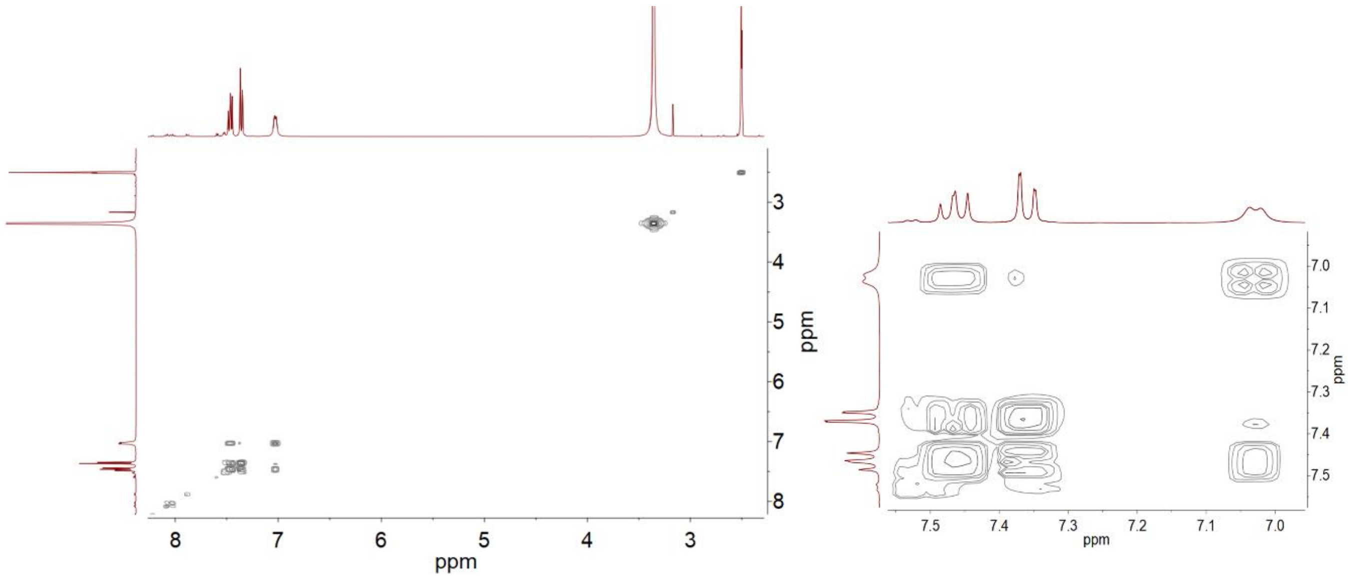

Supplementary Figure 2. ^{1}H−^{1}H COSY spectra of 6,6′-dithiol-2,2′-bipyridine in d_6-DMSO (left) and its enlargement of the aromatic region (right).

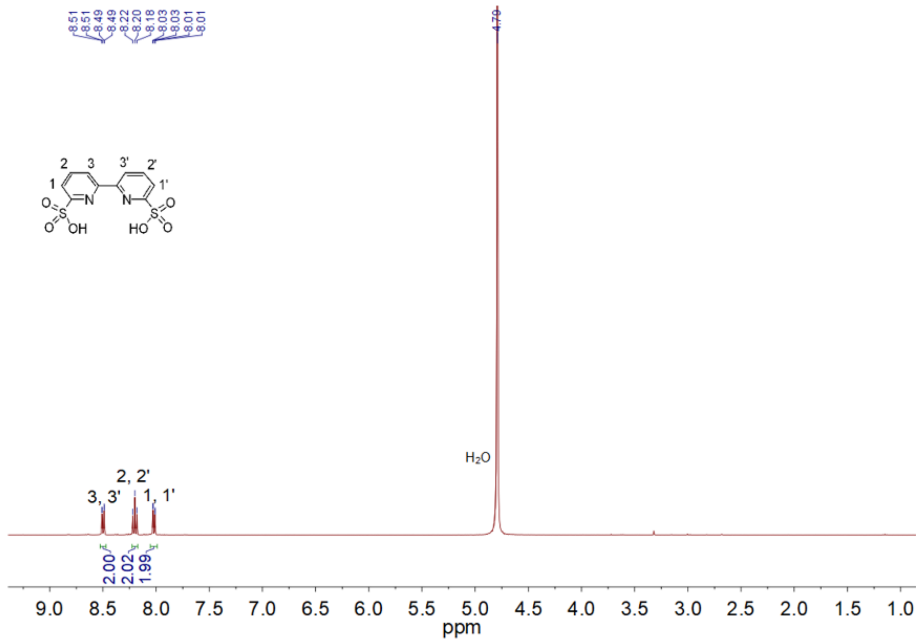

Supplementary Figure 3. ^{1}H NMR spectrum (400 MHz) of 2,2′-bipyridine-6,6′-disulfonic acid in D_2O.

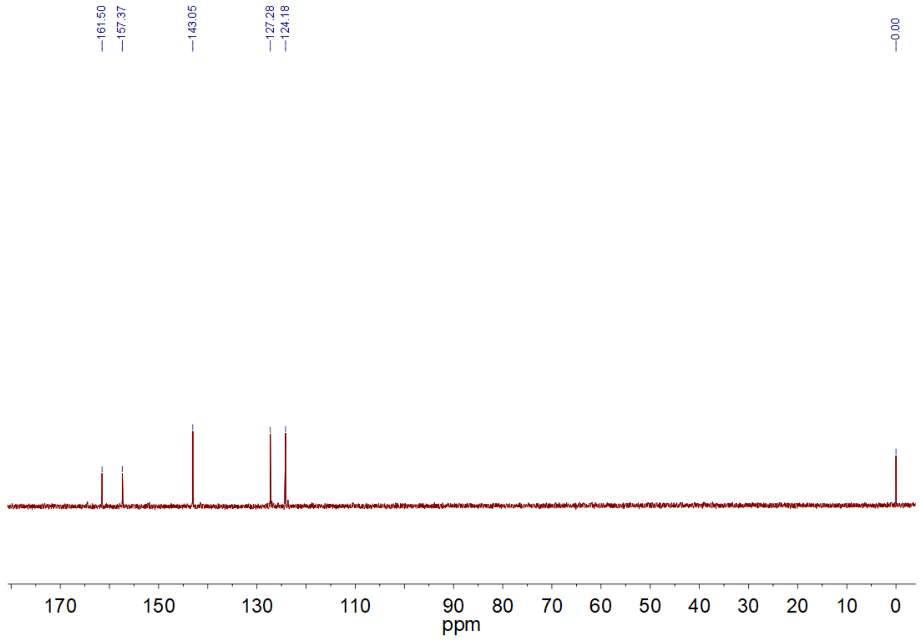

Supplementary Figure 4. ^{13}C NMR spectrum (101 MHz) of 2,2′-bipyridine-6,6′-disulfonic acid in D_2O.

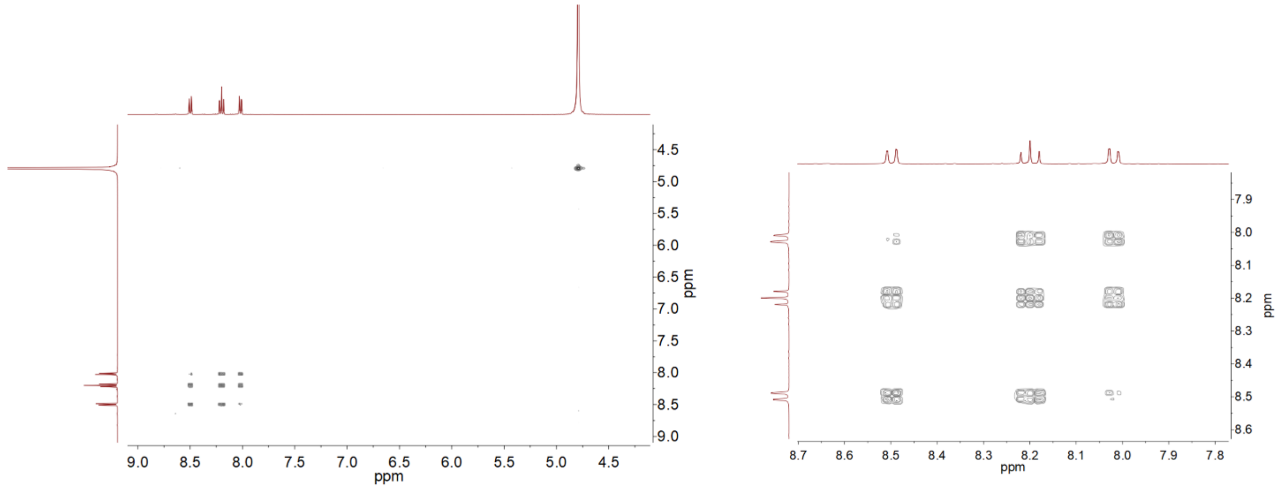

Supplementary Figure 5. ^1H–^1H COSY spectra of 2,2′-bipyridine-6,6′-disulfonic acid in D_2O (left) and its enlargement of the aromatic region (right).

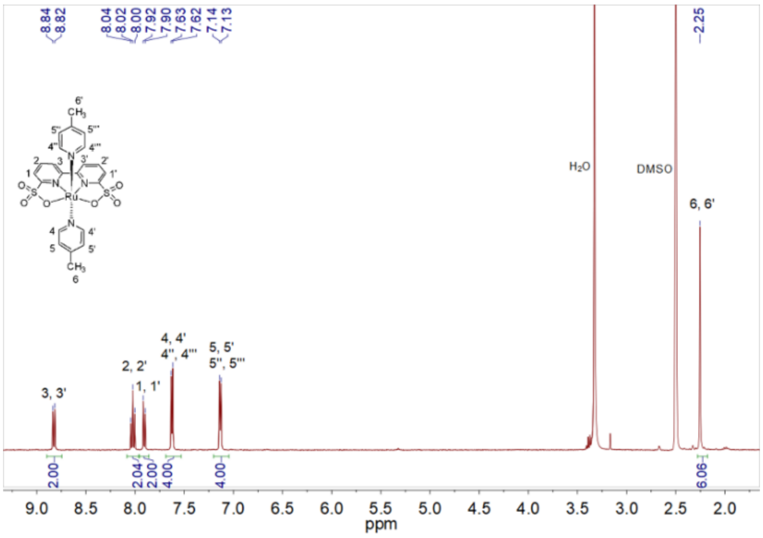

Supplementary Figure 6. ^1H NMR spectrum (400 MHz) of **Ru-bds** in d_6-DMSO.

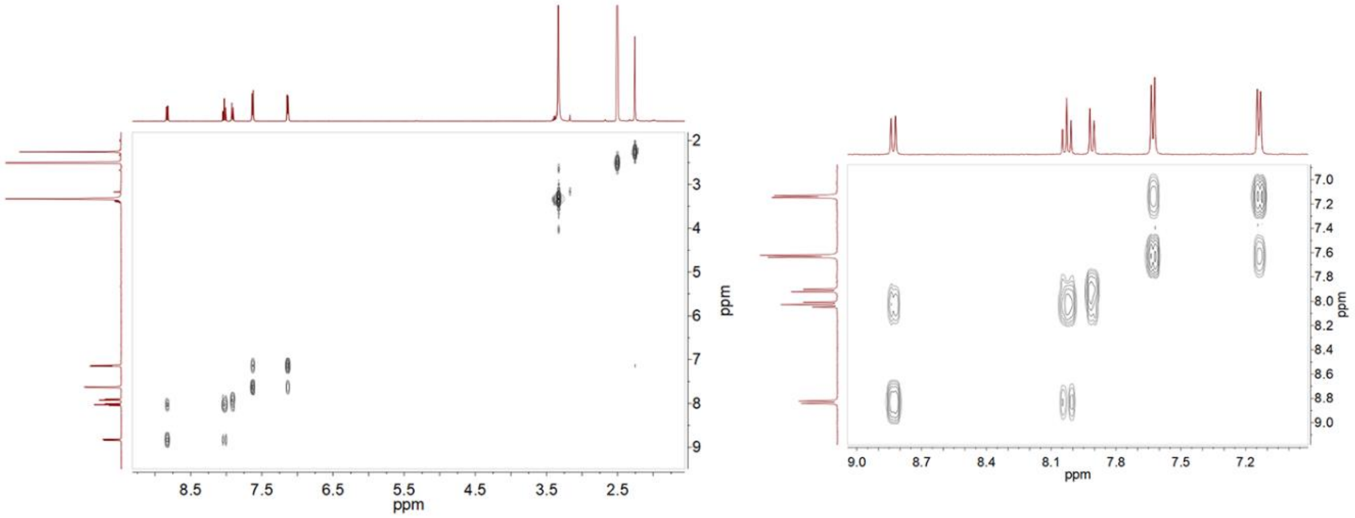

Supplementary Figure 7. ^1H–^1H COSY spectra of **Ru-bds** in d_6-DMSO (left) and its enlargement of the aromatic region (right).

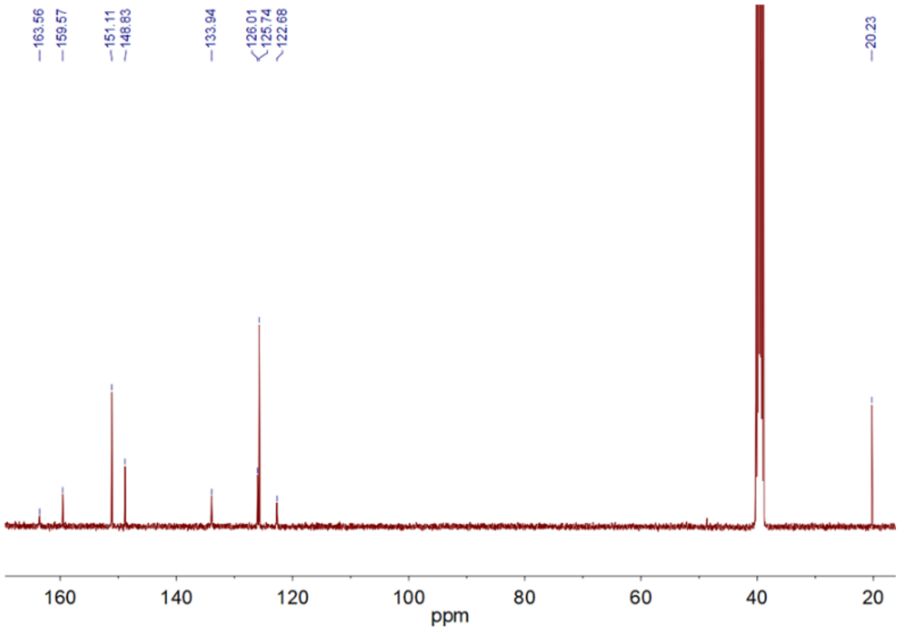

Supplementary Figure 8. ^{13}C NMR spectrum (101 MHz) of **Ru-bds** in d_6-DMSO.

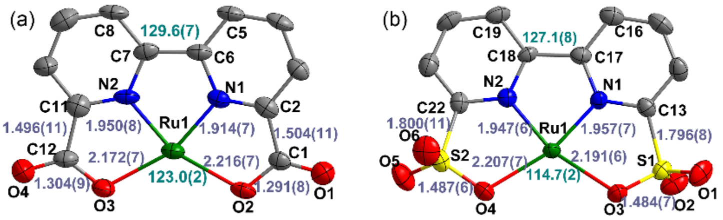

Supplementary Figure 9. The single-crystal X-ray structures of (a) **Ru-bda** and (b) **Ru-bds** ellipsoids at 50% probability, with selected bond distances and angles (axial ligands are omitted for clarity).

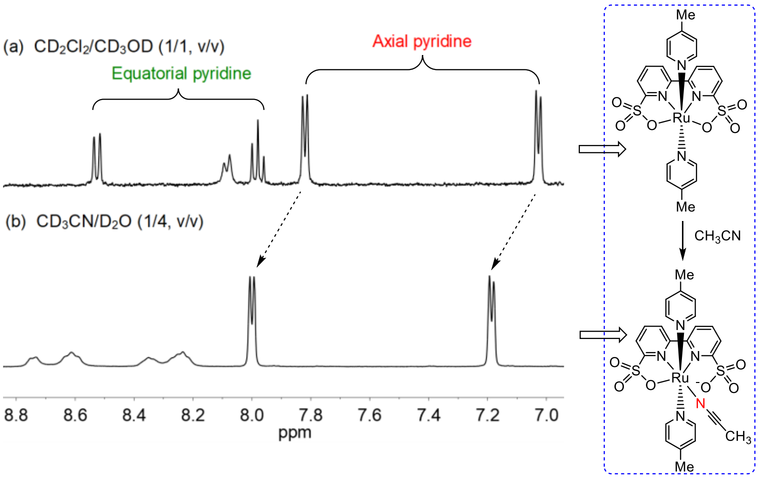

Supplementary Figure 10. Left: Aromatic region of ^{1}H NMR spectra (400 MHz) of **Ru-bds** in different solvents: a) CD_2Cl_2/CD_3OD (1/1, v/v) and b) CD_3CN/D_2O (1/4, v/v). Right: The reaction from [Ru($k_4^{O,O,N,N}$-bds)(pic)$_2$] (**Ru-bds**) to [Ru($k_3^{O,N,N}$-bds)(pic)$_2$(CH_3CN)].

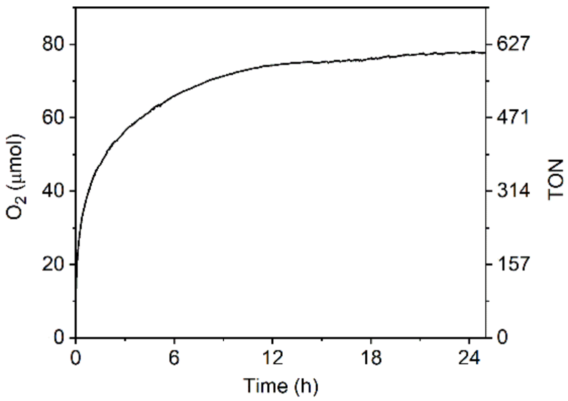

Supplementary Figure 11. Oxygen evolution curve of **Ru-bds**. [Ce^{IV}] = 0.122 M, [cat.] = 42.5 μM, and V = 3 mL, pH 1.0 triflic acid.

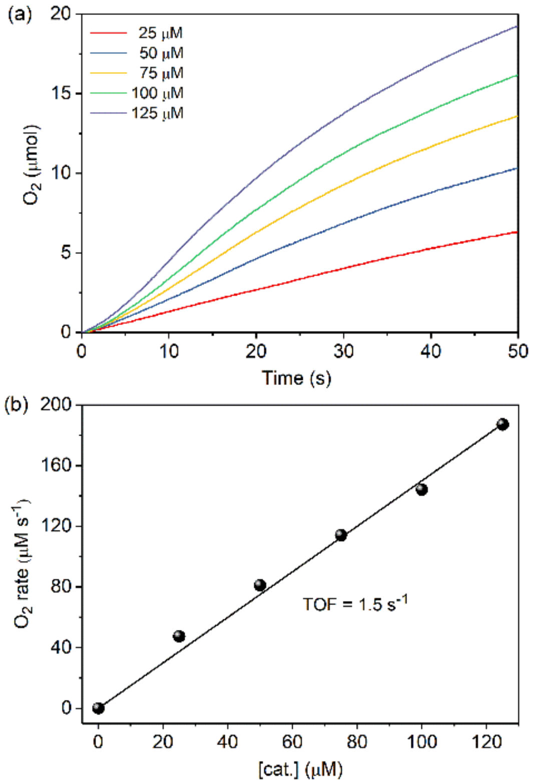

Supplementary Figure 12. (a) Initial phase of O_2 evolution versus time at various concentrations of **Ru-bds**. (b) Initial rate of O_2 evolution versus [cat.] based on the top oxygen evolution plots in the time interval of 5–15 s.

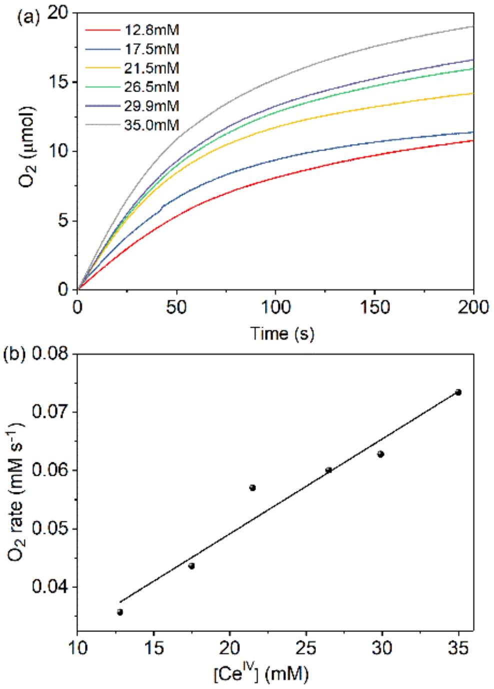

Supplementary Figure 13. (a) Initial phase of O_2 evolution by **Ru-bds** versus time at various concentrations of Ce^{IV}. (b) Plots of initial rate of O_2 evolution versus [Ce^{IV}] based on the top oxygen evolution plots in the time interval of 0–50 s.

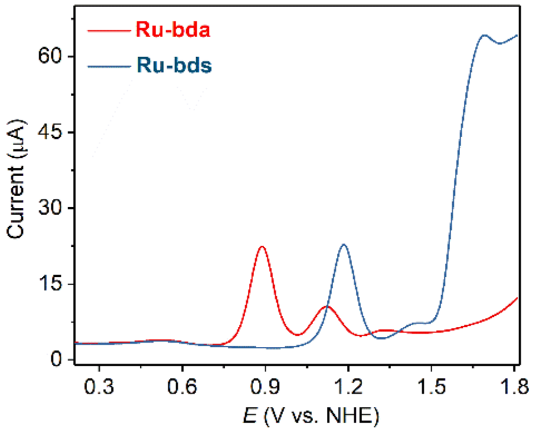

Supplementary Figure 14. DPVs of 1 mM **Ru-bda** and **Ru-bds** in pH 1.0 triflic acid aqueous solution containing 20% CH_3CN.

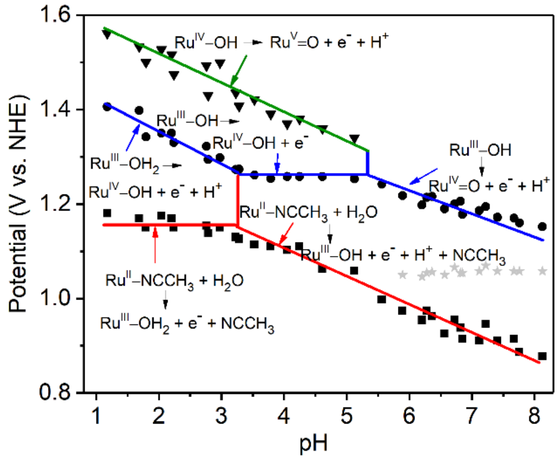

Supplementary Figure 15. Potential versus pH diagram for **Ru-bds** in 20% volume CH_3CN aqueous buffer solutions, data obtained from differential pulse voltammograms (DPVs, Supplementary Fig. 17). *The asterisks indicate an unknown process related to an unkown species.* The buffer system used in this study is phosphate buffer. Note that the potentials of $Ru^{V/IV}$ were measured at the current of 3.5×10^{-5} A from their DPVs while the potentials of $Ru^{IV/III}$ (from 5.3 to 8.1) were measured at the current of 3.0×10^{-5} A from their DPVs.

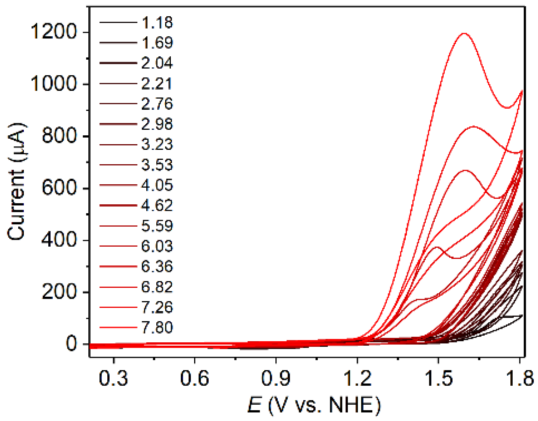

Supplementary Figure 16. Selected CVs of 1 mM **Ru-bds** under various pH conditions.

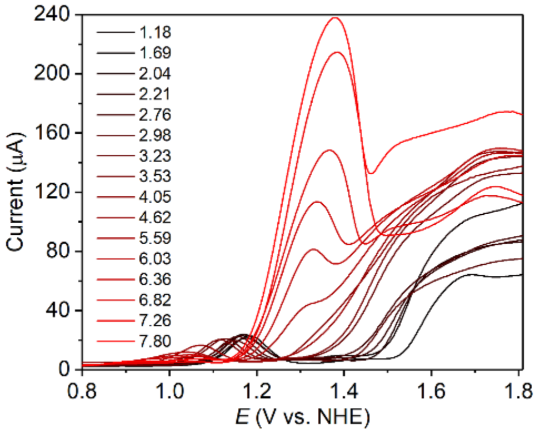

Supplementary Figure 17. Selected DPVs of 1 mM **Ru-bds** under various pH conditions.

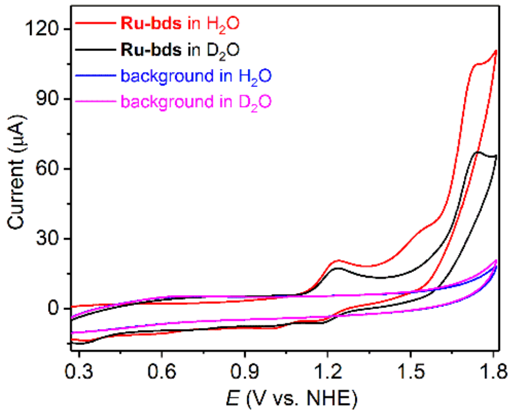

Supplementary Figure 18. CVs of 1 mM **Ru-bds** in D_2O and H_2O solutions containing pH 1.0 triflic acid aqueous solution with 20% volume CH_3CN.

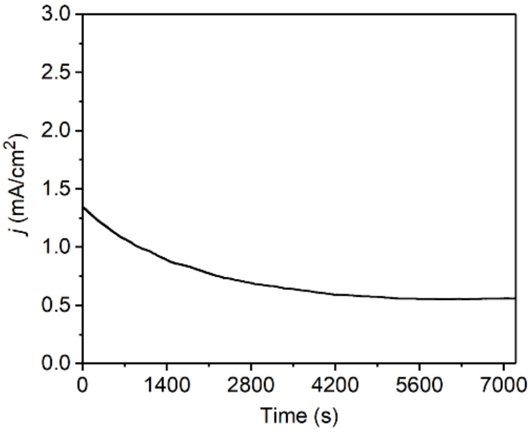

Supplementary Figure 19. Bulk electrolysis with 0.5 mM **Ru-bds** at 1.7 V (versus NHE) in CH_3CN/pH 1.0 triflic acid solutions (1/4, v/v).

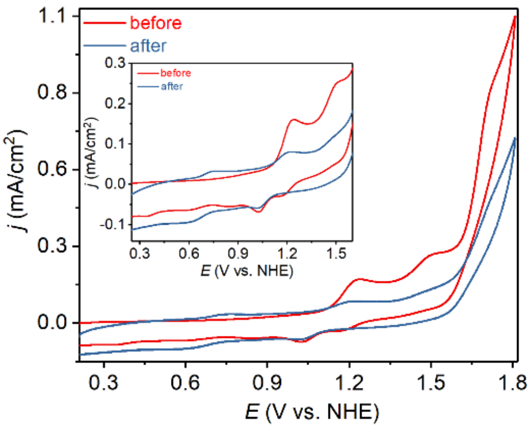

Supplementary Figure 20. CVs of 0.5 mM **Ru-bds** in CH_3CN/pH 1.0 triflic acid solutions (1/4, v/v) before and after bulk electrolysis. Inset: Enlargement of the 0.25−1.60 V zone in the CV.

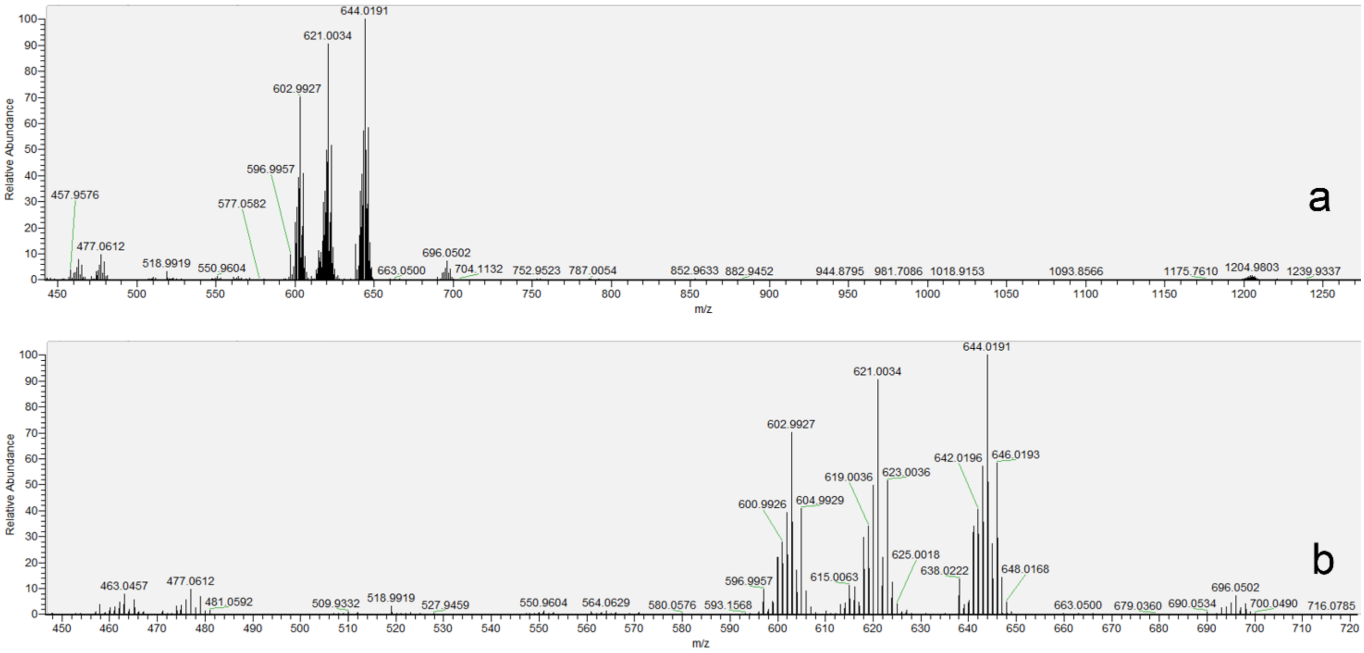

Supplementary Figure 21. HRMS spectra recorded before bulk electrolysis solution from 0.5 mM **Ru-bds** in CH_3CN/pH 1.0 triflic acid solutions (1/4, v/v) with (a) *m/z* from 450 to 1270 and (b) enlargement of *m/z* 450–720 zone.

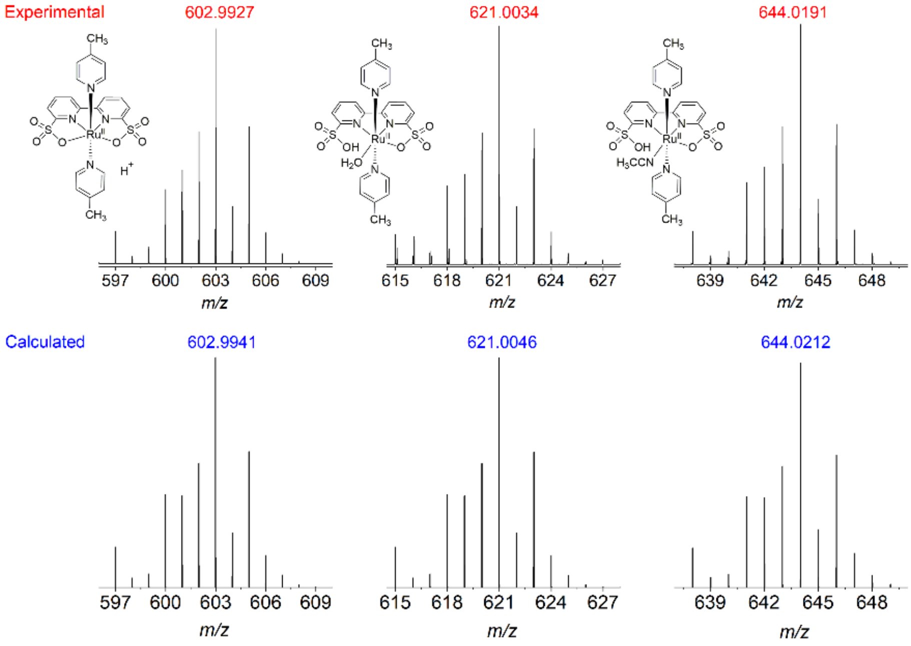

Supplementary Figure 22. HRMS spectra of $[Ru^{II}(bds^{2-})(pic)_2+H]^+$, $[Ru^{II}(bdsH^{-})(pic)_2(H_2O)]^+$ and $[Ru^{II}(bdsH^{-})(pic)_2(CH_3CN)]^+$ (top) and calculated isotopic distribution (bottom).

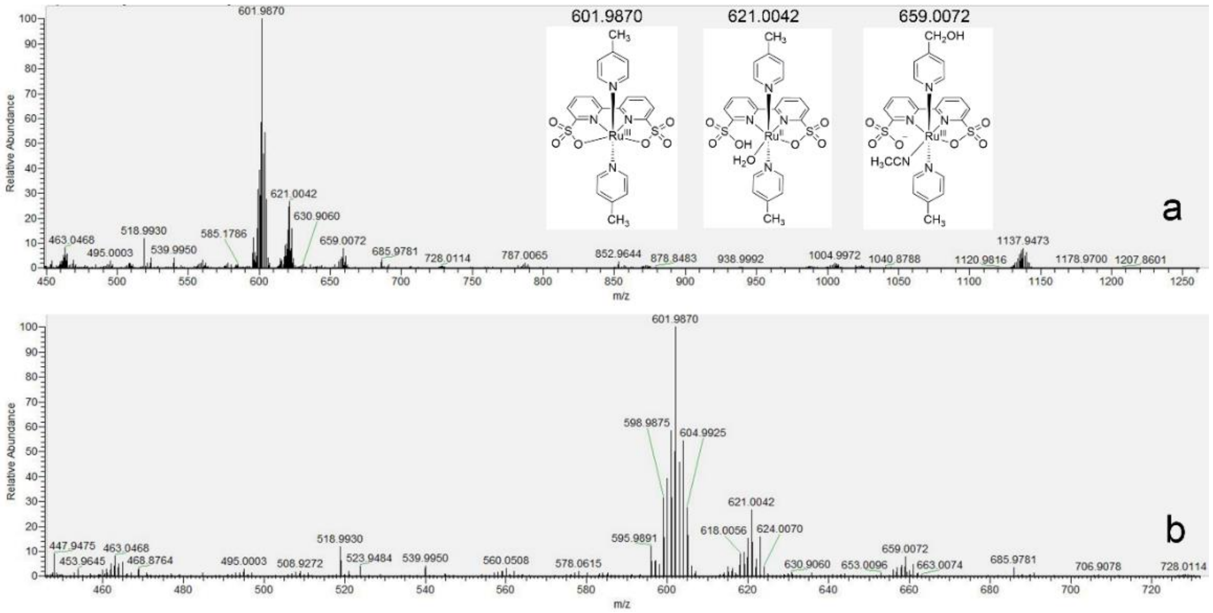

Supplementary Figure 23. HRMS spectra recorded after bulk electrolysis solution from 0.5 mM **Ru-bds** in CH_3CN/pH 1.0 triflic acid solutions (1/4, v/v) with (a) *m/z* from 450 to 1270 and (b) enlargement of *m/z* 450–720 zone.

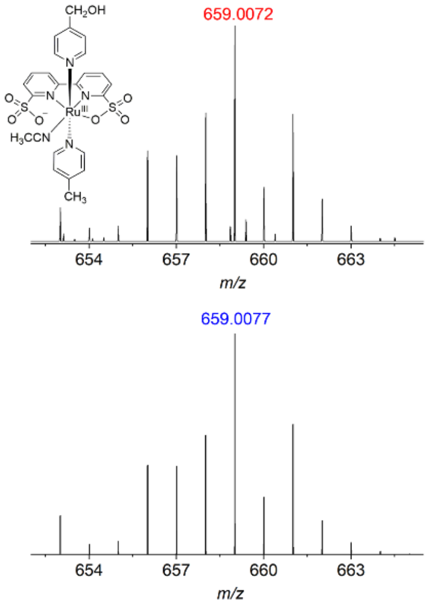

Supplementary Figure 24. HRMS spectra of $[Ru^{III}(k_3^{O,N,N}\text{-bds}^{2-})(pic)(pym)(CH_3CN)]^+$ (top) and calculated isotopic distribution (bottom).

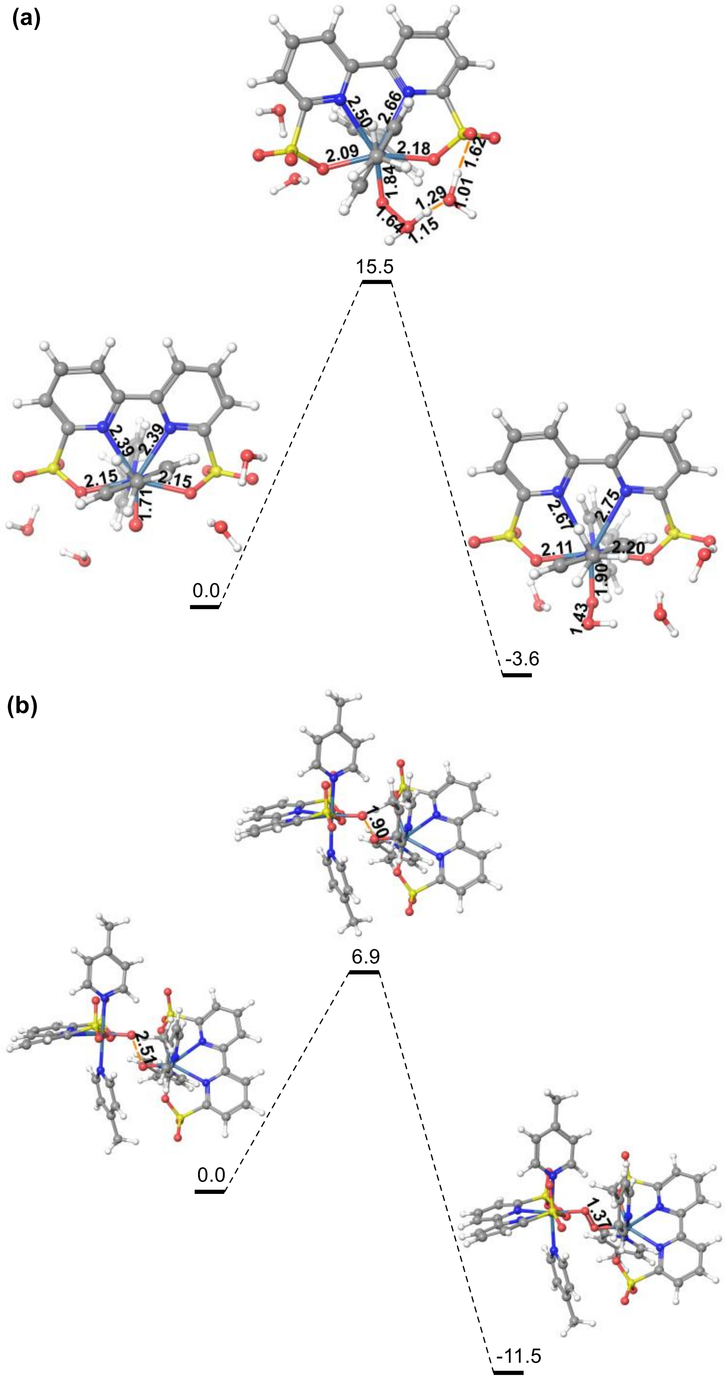

Supplementary Figure 25. The energy profile of O−O bond formation of $[Ru^{V}=O]^{+}$ with the (a) WNA and (b) I2M mechanisms at pH 1.0. The unit of energy is kcal mol^{-1}. The bond lengths are given in Å.

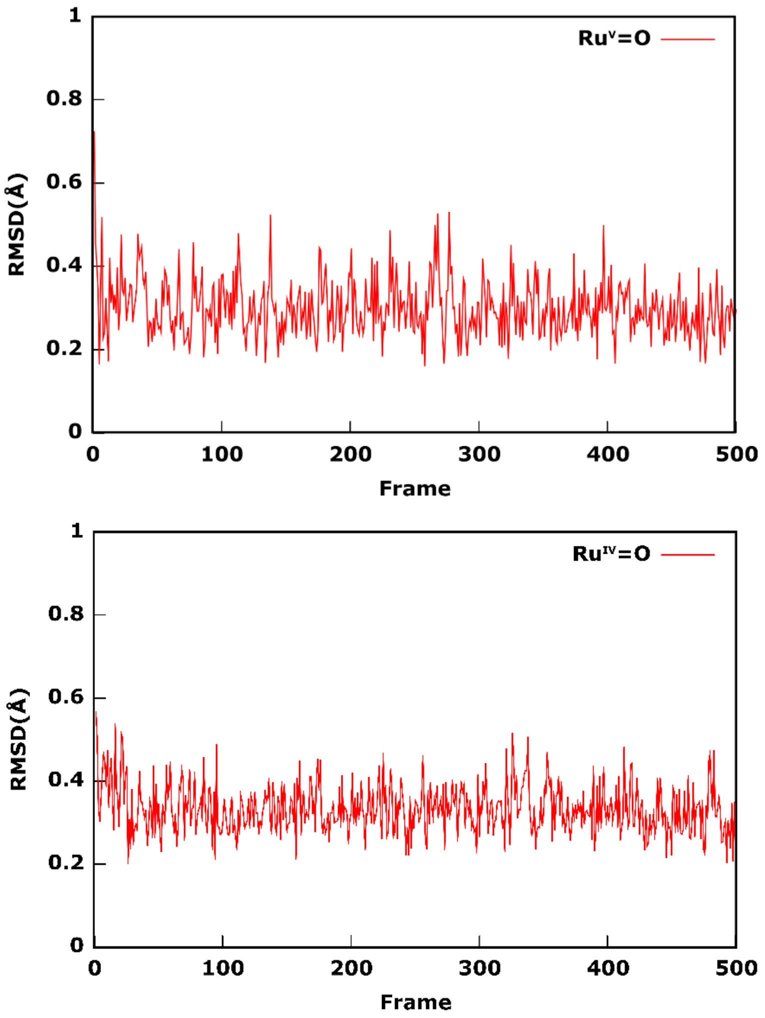

Supplementary Figure 26. The root mean square deviation (RMSD) plots of $[Ru^{V}=O]^{+}$ (top) and $Ru^{IV}=O$ (bottom) with respect to their initial structures obtained from the geometry optimization using DFT. The figure plotted during a 1 ns molecular dynamics (MD) simulation in a 20 Å radius sphere simulation. Both $[Ru^{V}=O]^{+}$ and $Ru^{IV}=O$ behaved stable in MD simulation.

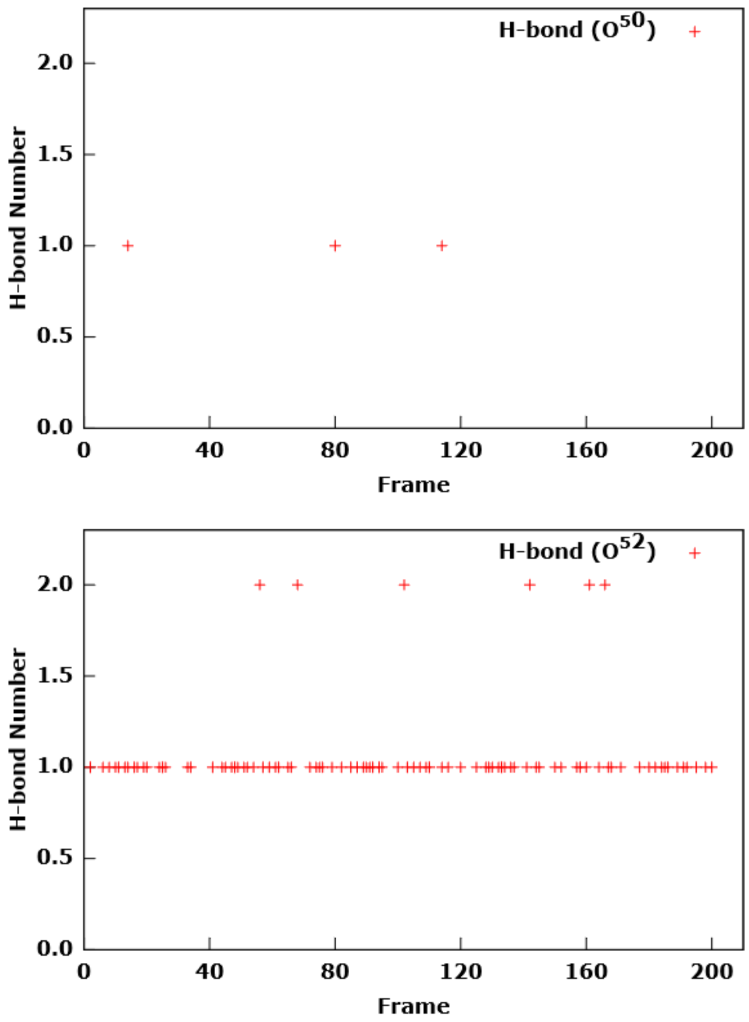

Supplementary Figure 27. Hydrogen-bonding analysis between O of $[Ru^{V}=O]^{+}$ and water. Numberings of atoms are shown in **Supplementary Figure 30.**

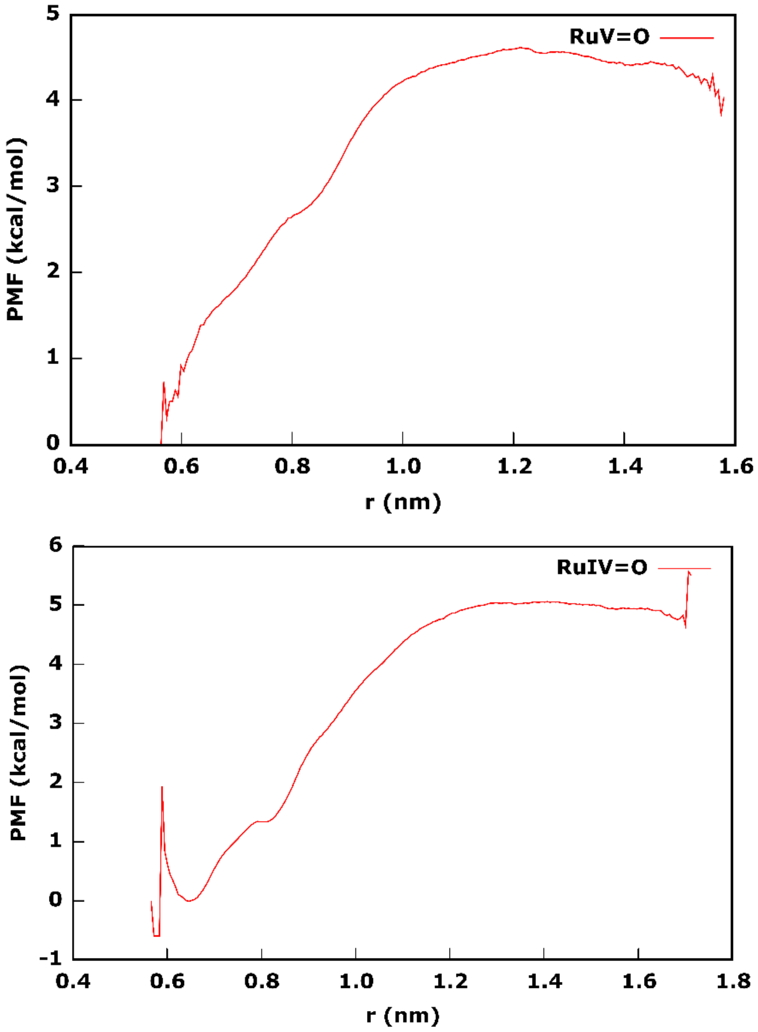

Supplementary Figure 28. The potential of mean force (PMF) profile of $[Ru^{V}=O]^{+}$ (top) and $Ru^{IV}=O$ (bottom) in the water phase. The profile describes the binding energy of two complexes as a function of the Ru--Ru coordinate.

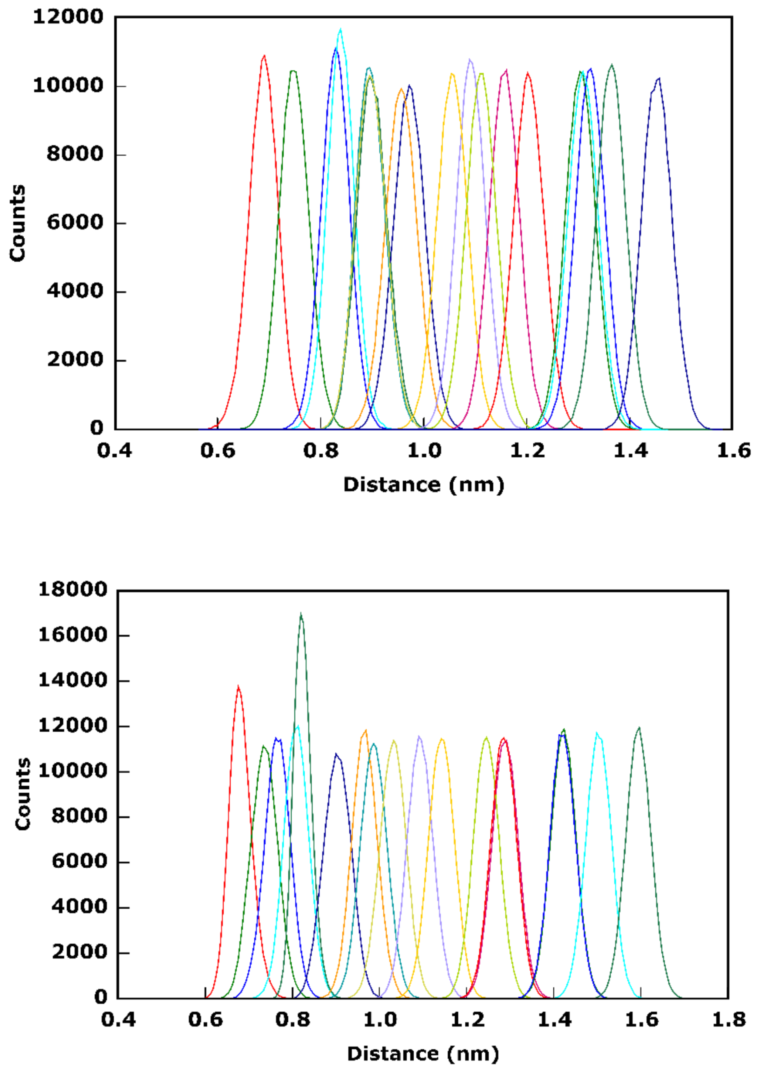

Supplementary Figure 29. The umbrella histograms of [Ru^{V}=O]$^{+}$ (top) and Ru^{IV}=O (bottom) in the water phase. The above histograms show reasonable overlap between windows from about 0.4 - 1.6 nm of COM spacing and 0.4 - 1.8 nm of COM spacing, respectively.

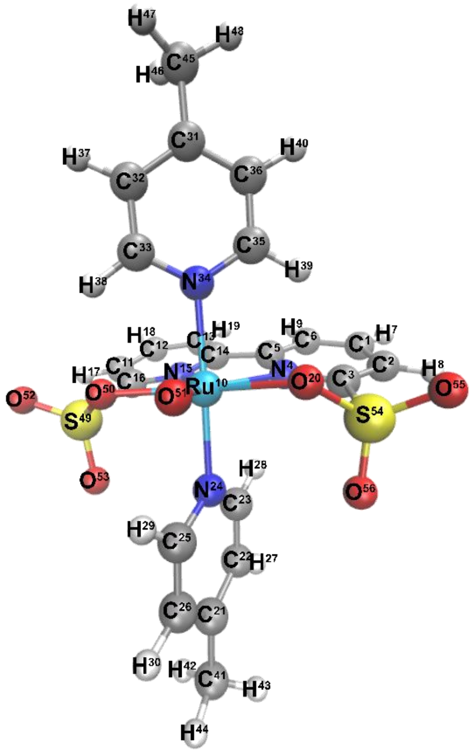

Supplementary Figure 30. Numberings of atoms constituting the complexes parameterized in this study.

Supplementary Table 1. The energy difference of $[Ru^{IV}\text{-}OH]^+$ at triplet and singlet (G_{T1}-G_{S0}) calculated by different functionals.

Functional	B3LYP-D3	PBE	M06-L
ΔG(kcal/mol)	-5.64	0.94	-0.32

The DFT calculation showed that the energy difference of $[Ru^{IV}\text{–}OH]^+$ at triplet and singlet is small, and smaller using functionals without Hartree-Fock exchange. In addition, the $[Ru^{IV}\text{–}OH]^+$ species of the **Ru-bda** catalyst has been experimentally characterized as a singlet (*J. Am. Chem. Soc.* 2009, 131, 10397-10399.). We used the singlet structure of the $[Ru^{IV}\text{–}OH]^+$ species of the **Ru-bds** complex in the paper which gives very good agreement with the experimental $Ru^{VI/III}$ potential. It cannot be excluded that the triplet is also present.

Supplementary Table 2. Activation free energies (ΔΔG) for radical coupling and dissociation energies (ΔG_{diss}) of complexes **Ru-bds** and **Ru-bda**. (All values are in kcal mol^{-1})

	ΔΔG	ΔG_{diss}
**Ru-bds**	6.6	4.0
**Ru-bda**	7.1	3.2

All values in ΔΔG are calculated by DFT with the PBF solvation model. Values in ΔG_{diss} are calculated with PMF in an explicit TIP3P water phase.

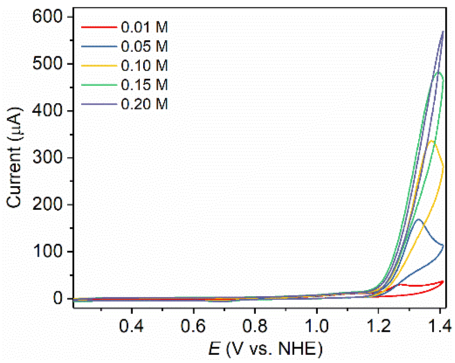

Supplementary Figure 31. CVs of 1 mM **Ru-bds** at pH 7.0 in $H_2PO_4^{-}/HPO_4^{2-}$ buffers, and buffer concentrations are 0.01 M (grey), 0.05 M (green), 0.10 M (yellow), 0.15 M (blue) and 0.20 M (red), I = 0.5 M ($NaNO_3$), scan rate is 20 mV/s.

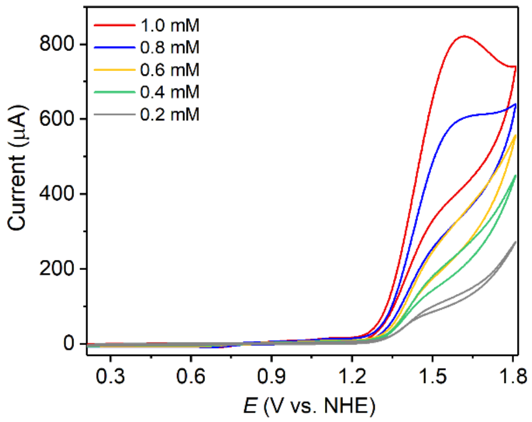

Supplementary Figure 32. CVs of **Ru-bds** at pH 7.0 in 0.2 M $H_2PO_4^{-}/HPO_4^{2-}$ buffers, and catalyst concentrations are 0.2 mM (grey), 0.4 mM (green), 0.6 mM (yellow), 0.8 mM (blue), and 1.0 mM (red), I = 0.5 M ($NaNO_3$), scan rate is 100 mV/s.

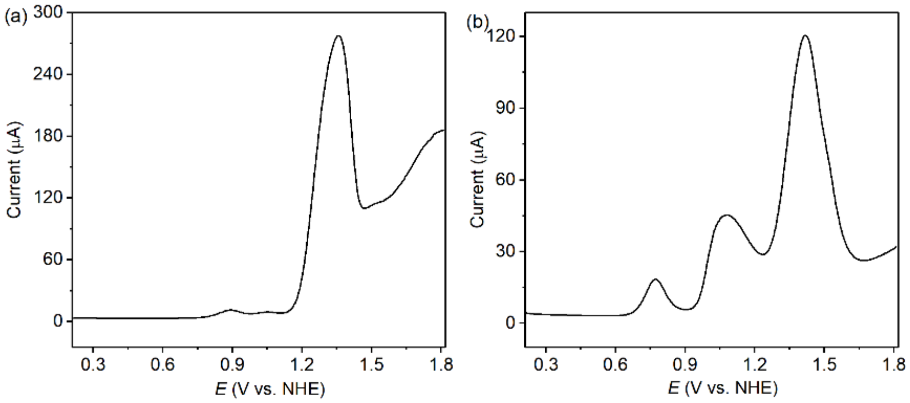

Supplementary Figure 33. DPVs of (a) 1 mM **Ru-bds** and (b) 1 mM **Ru-bda** at pH 7.0 phosphated buffer containing 20% CH_3CN.

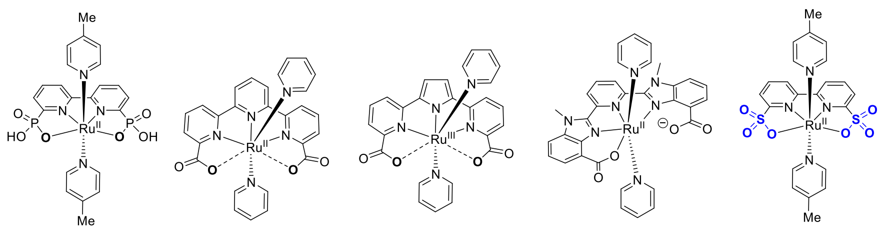

Ru WOCs	pH	onset potential (V)	peak/plateau potential (V)	catalytic current density at 1.5 V (mA cm^{-2})
**1**	6.5	1.20	1.53	1.8
**2**	7.0	1.31	1.58	3.7
**3**	7.0	1.28	1.48	1.5
**4**	7.0	1.27	1.54	4.3
**5** (**Ru-bds**)	7.0	1.20	1.63	9.0

Supplementary Table 3. Key parameters of complexes **1-5** from CV curves and their corresponding chemical structures.[1-4]

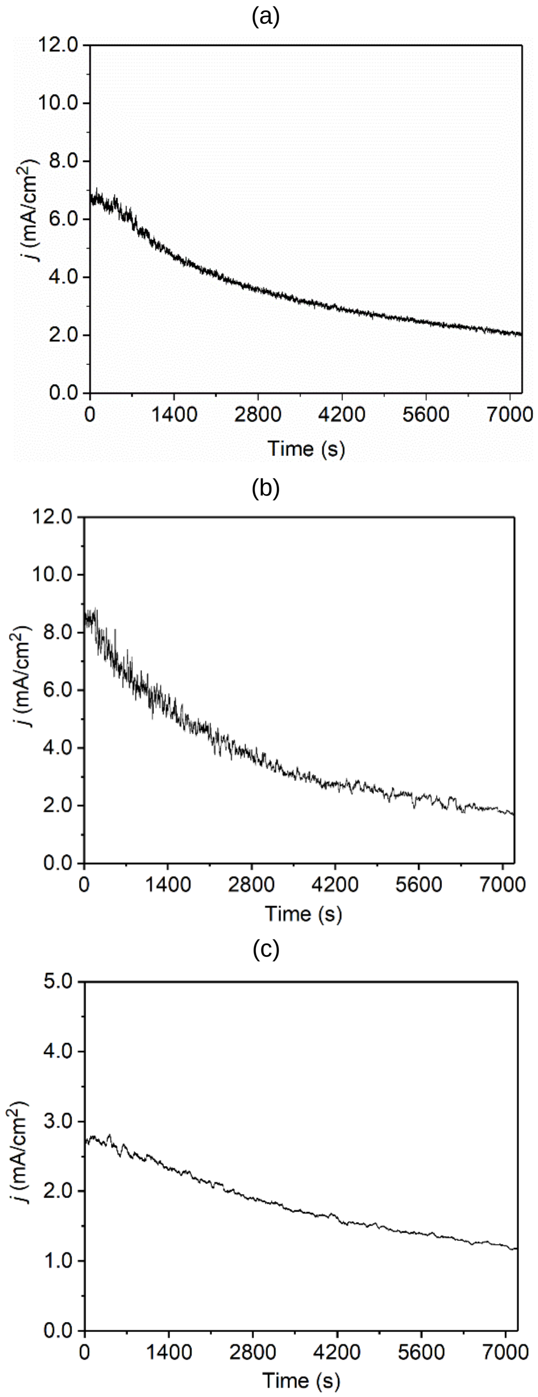

Supplementary Figure 34. (a) Bulk electrolysis with 0.5 mM **Ru-bds** at 1.5 V (versus NHE) in CH_3CN/pH 7.0 phosphate buffer (1/4, v/v). (b) Bulk electrolysis with 0.5 mM **Ru-bds** at 1.7 V (versus NHE) in CH_3CN/pH 7.0 phosphate buffer (1/4, v/v). (c) Bulk electrolysis with 0.5 mM **Ru-bds** at 1.35 V (versus NHE) in CH_3CN/pH 7.0 phosphate buffer (1/4, v/v).

The CV data (Fig. 3a in the manuscript) shows that the catalytic current density at 1.5 V was 8.8 mA/cm^2 in pH 7, which was about 6.7 times (a factor of 45 for TOF) than that at 1.7 V in pH 1 (1.3 mA/cm^2, Fig. 3a in the manuscript). And this is slightly higher than the result of bulk electrolysis experiments (5 times, a factor of 25 for TOF) as shown from Supplementary Figure 19 and Supplementary Figure 34a.

To perform at more comparable potentials, bulk electrolysis experiment of pH 7 was carried out at 1.7 V (Supplementary Figure 34b) to keep the same electrolytic potential with that in pH 1 (Supplementary Figure 19). The current density this time reached about 8.8 mA/cm^2, which are about 6.8 times (a factor of 45 for TOF) higher than that at pH 1. This is also close to the corresponding CV data (8.7 times, a factor of 76 for TOF), during which the catalytic

current density in pH 7 at 1.7 V from CV is 11.2 mA/cm^2. It's worth to note that the moderate difference of current density between bulk electrolysis and CV is reasonable due to the different operation conditions.

Additionally, the bulk electrolysis experiments were performed with the same overpotential of 530 mV. The bulk electrolysis at pH 7 were carried out at 1.35 V (Supplementary Figure 34c), and the current density was about 2.8 mA/cm^2, which are around two times (a factor of 4 for TOF) higher than that at pH 1. This is consistent with CV data. Because the catalytic current density in pH 7 at 1.35 V is 2.49 mA/cm^2, also two times (a factor of 4 for TOF) than that in pH 1 at 1.7 V of 1.28 mA/cm^2.

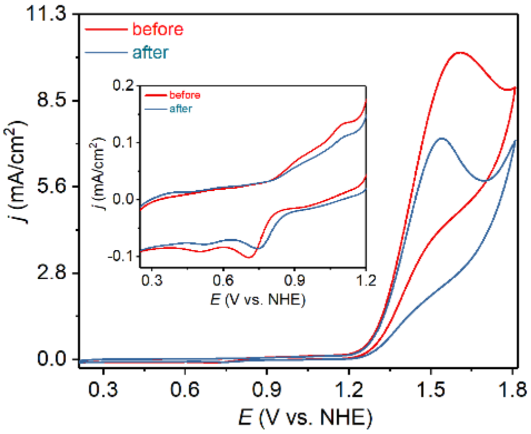

Supplementary Figure 35. CVs of 0.5 mM **Ru-bds** in CH_3CN/pH 7.0 phosphate buffer (1/4, v/v) before and after bulk electrolysis. Inset: Enlargement of the 0.25−1.2 V zone in the CV.

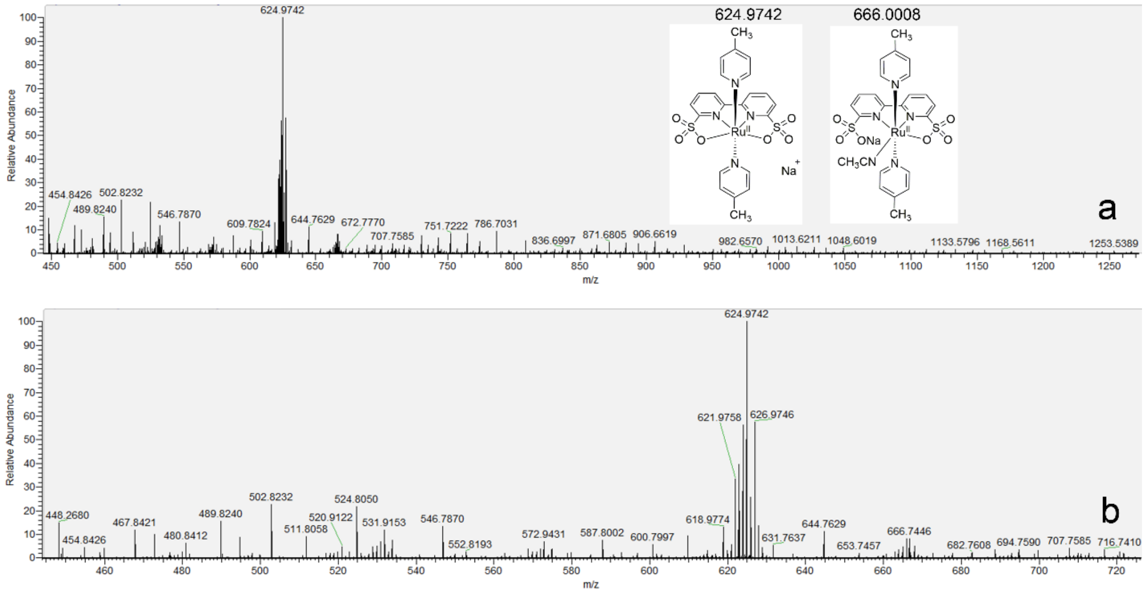

Supplementary Figure 36. HRMS spectra recorded before bulk electrolysis solution from 0.5 mM **Ru-bds** in CH_3CN/pH 7.0 phosphate buffer (1/4, v/v) with (a) *m/z* from 450 to 1270 and (b) enlargement of *m/z* 450–720 zone.

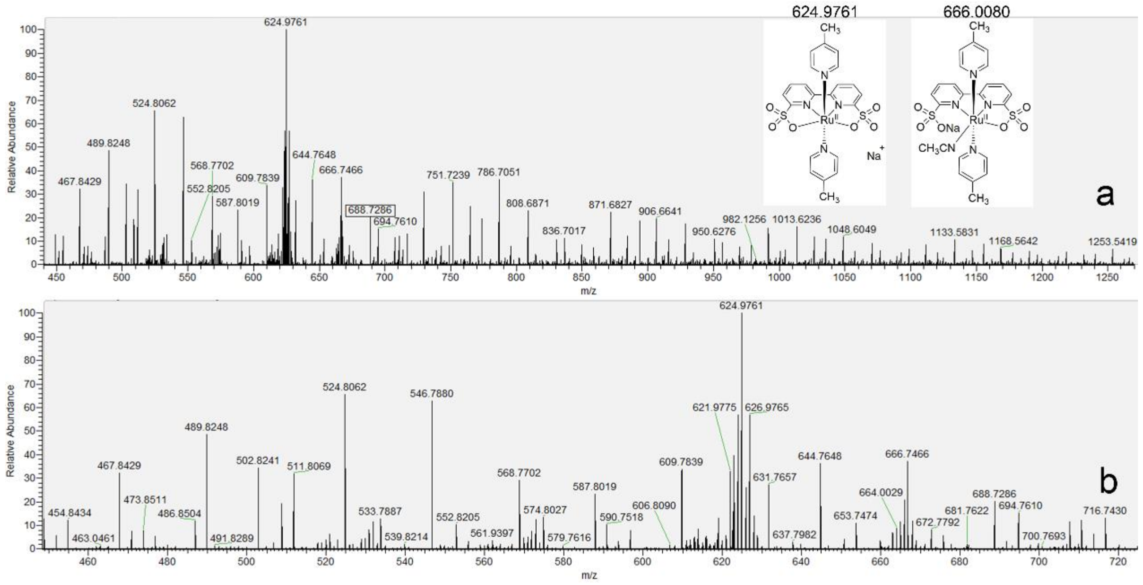

Supplementary Figure 37. HRMS spectra recorded after bulk electrolysis solution from 0.5 mM **Ru-bds** in CH_3CN/pH 7.0 phosphate buffer (1/4, v/v) with (a) *m/z* from 450 to 1270 and (b) enlargement of *m/z* 450–720 zone.

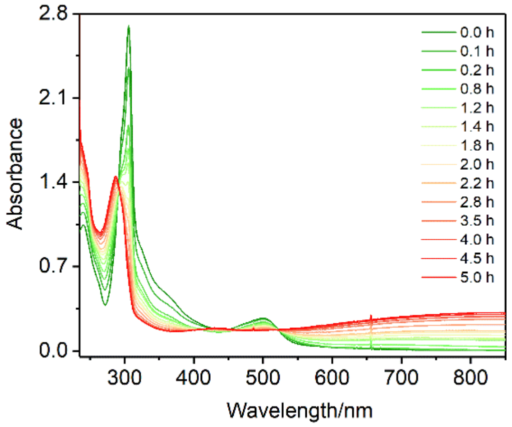

Supplementary Figure 38. UV-vis spectral changes of 0.5 mM **Ru-bds** in phosphate buffer solution (pH 7, 0.2 M, 20% CH_3CN) at a constantly applied potential of 1.21 V. The path length of the quartz cuvette is 1 mm. The quartz cuvette was filled with 1 mL 0.5 mM **Ru-bds** in 20% CH_3CN-phosphate buffer solution. Pt mesh as working electrode, a Pt wire as counter electrode and an Ag/AgCl reference electrode.

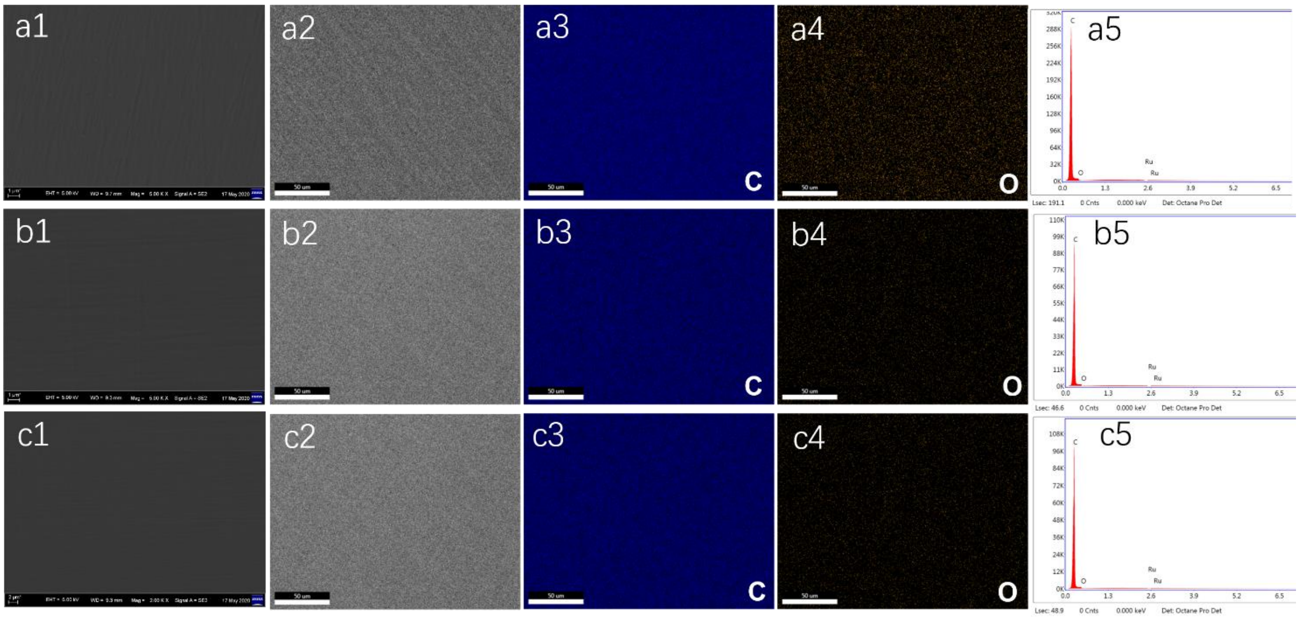

Supplementary Figure 39. SEM images of bare GC (a1 and a2), and corresponding EDX mapping (a3 and a4) and EDX spectrum (a5). SEM images of post-electrocatalyzed electrode surface at pH 1 (b1 and b2), and corresponding EDX mapping (b3 and b4) and EDX spectrum (b5). SEM images of post-electrocatalyzed electrode surface at pH 7 (c1 and c2), and corresponding EDX mapping (c3 and c4) and EDX spectrum (c5).

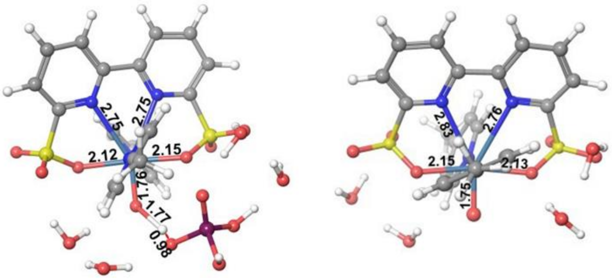

Supplementary Figure 40. DFT optimized geometries for the structure of $[Ru^{IV}{-}OH]^{+}$ with $H_2PO_4^{-}$ ion (left) and $Ru^{IV}{=}O_T$ (right) species. The bond lengths are given in Å.

Supplementary Table 4. List of atoms types and corresponding van der Waals parameters used to describe the atoms in the complexes $[Ru^{V}{=}O]^{+}$ and $Ru^{IV}{=}O$.

Van Der Waals Parameters Used for the Atoms in the Complexes					
**Atom type**	**A(kcal^{½}mol$^{-½}$Å^{6})**	**B(kcal^{½}mol$^{-½}$Å^{3})**	**A_{1-4}(kcal^{½}mol$^{-½}$Å^{6})**	**B_{1-4}(kcal^{½}mol$^{-½}$Å^{3})**	**Mass (a.u.)**
C1	1059.13	23.6736	748.9178	16.7398	12.011
C11	1059.13	23.6736	748.9178	16.7398	12.011
C12	1059.13	23.6736	748.9178	16.7398	12.011
C13	1059.13	23.6736	748.9178	16.7398	12.011
C14	1059.13	23.6736	748.9178	16.7398	12.011
C16	1059.13	23.6736	748.9178	16.7398	12.011
C2	1059.13	23.6736	748.9178	16.7398	12.011
C21	1059.13	23.6736	748.9178	16.7398	12.011
C22	1059.13	23.6736	748.9178	16.7398	12.011
C23	1059.13	23.6736	748.9178	16.7398	12.011
C25	1059.13	23.6736	748.9178	16.7398	12.011
C26	1059.13	23.6736	748.9178	16.7398	12.011
C3	1059.13	23.6736	748.9178	16.7398	12.011
C31	1059.13	23.6736	748.9178	16.7398	12.011
C32	1059.13	23.6736	748.9178	16.7398	12.011
C33	1059.13	23.6736	748.9178	16.7398	12.011
C35	1059.13	23.6736	748.9178	16.7398	12.011
C36	1059.13	23.6736	748.9178	16.7398	12.011
C41	944.518	22.0296	667.8751	15.5773	12.011
C45	944.518	22.0296	667.8751	15.5773	12.011
C5	1059.13	23.6736	748.9178	16.7398	12.011
C6	1059.13	23.6736	748.9178	16.7398	12.011
H17	69.5797	4.9095	49.2003	3.4715	1.0079
H18	69.5797	4.9095	49.2003	3.4715	1.0079
H19	69.5797	4.9095	49.2003	3.4715	1.0079
H27	69.5797	4.9095	49.2003	3.4715	1.0079

H28	69.5797	4.9095	49.2003	3.4715	1.0079
H29	69.5797	4.9095	49.2003	3.4715	1.0079
H30	69.5797	4.9095	49.2003	3.4715	1.0079
H37	69.5797	4.9095	49.2003	3.4715	1.0079
H38	69.5797	4.9095	49.2003	3.4715	1.0079
H39	69.5797	4.9095	49.2003	3.4715	1.0079
H40	69.5797	4.9095	49.2003	3.4715	1.0079
H42	84.5728	5.4127	59.802	3.8274	1.0079
H43	84.5728	5.4127	59.802	3.8274	1.0079
H44	84.5728	5.4127	59.802	3.8274	1.0079
H46	84.5728	5.4127	59.802	3.8274	1.0079
H47	84.5728	5.4127	59.802	3.8274	1.0079
H48	84.5728	5.4127	59.802	3.8274	1.0079
H7	69.5797	4.9095	49.2003	3.4715	1.0079
H8	69.5797	4.9095	49.2003	3.4715	1.0079
H9	69.5797	4.9095	49.2003	3.4715	1.0079
N15	971.7502	28.3077	687.1312	20.0166	14.007
N24	971.7502	28.3077	687.1312	20.0166	14.007
N34	971.7502	28.3077	687.1312	20.0166	14.007
N4	971.7502	28.3077	687.1312	20.0166	14.007
O20	554.6317	21.386	392.1838	15.1222	15.999
O50	554.6317	21.386	392.1838	15.1222	15.999
O51	601.1488	22.2648	425.0764	15.7436	15.999
O52	554.6317	21.386	392.1838	15.1222	15.999
O53	554.6317	21.386	392.1838	15.1222	15.999
O55	554.6317	21.386	392.1838	15.1222	15.999
O56	554.6317	21.386	392.1838	15.1222	15.999
S49	2001.567	44.7389	1415.322	31.6352	32.065
S54	2001.567	44.7389	1415.322	31.6352	32.065
Ru10	1879.87	47.83	1329.27	33.82	101.07

Supplementary Table 5. Atom types and partial charges assigned to atoms in the complexes.

$[Ru^{V}=O]^{+}$		$Ru^{IV}=O$	
**Atom type**	**Charge**	**Atom type**	**Charge**
C1	0.011278	C1	0.035
C2	-0.12717	C2	-0.21662
C3	0.023388	C3	0.178757
N4	-0.02979	N4	-0.33878
C5	0.091298	C5	0.254906
C6	-0.1872	C6	-0.26145
H7	0.146575	H7	0.107571
H8	0.186728	H8	0.162368
H9	0.172076	H9	0.138116
Ru10	0.256031	Ru10	0.854421
C11	-0.14243	C11	-0.21734
C12	0.024017	C12	0.043441
C13	-0.20487	C13	-0.25485
C14	0.146784	C14	0.158632
N15	-0.08897	N15	-0.20666
C16	0.054215	C16	0.098144
H17	0.189782	H17	0.168618
H18	0.148541	H18	0.108851
H19	0.166278	H19	0.147018
O20	-0.37857	O20	-0.49035
C21	0.416918	C21	0.396826
C22	-0.27246	C22	-0.31222
C23	0.012492	C23	0.16514
N24	-0.05096	N24	-0.26635
C25	0.095859	C25	0.139978
C26	-0.30662	C26	-0.32859
H27	0.173869	H27	0.169416

H28	0.146036	H28	0.042213
H29	0.144347	H29	0.123375
H30	0.18719	H30	0.172641
C31	0.415983	C31	0.386993
C32	-0.35433	C32	-0.31606
C33	0.115221	C33	0.139038
N34	-0.04923	N34	-0.23618
C35	0.089617	C35	0.11525
C36	-0.32908	C36	-0.29432
H37	0.21166	H37	0.163414
H38	0.090797	H38	0.121805
H39	0.100128	H39	0.076042
H40	0.196476	H40	0.162157
C41	-0.4969	C41	-0.47944
H42	0.158556	H42	0.13683
H43	0.165649	H43	0.148234
H44	0.153122	H44	0.141933
C45	-0.50582	C45	-0.47685
H46	0.160081	H46	0.137373
H47	0.160151	H47	0.144738
H48	0.17092	H48	0.138826
S49	0.94714	S49	0.965888
O50	-0.37112	O50	-0.47964
O51	-0.07946	O51	-0.31776
O52	-0.4717	O52	-0.52167
O53	-0.48423	O53	-0.5272
S54	0.964312	S54	0.964089
O55	-0.47224	O55	-0.53108
O56	-0.49037	O56	-0.53467

Supplementary Table 6. Bond parameters used to describe the atoms in the complexes $[Ru^{V}=O]^{+}$ and $Ru^{IV}=O^{a}$.

Bond Stretch Parameters in the Complexes			
Bond types		K_b (kcal mol^{-1} Å^{-2})	r_0 (Å)
C1	C2	938	1.4
C1	C6	938	1.4
C1	H7	734	1.08
C11	C12	938	1.4
C11	C16	938	1.4
C11	H17	734	1.08
C12	C13	938	1.4
C12	H18	734	1.08
C13	C14	938	1.4
C13	H19	734	1.08
C14	C5	750	1.464
C14	N15	966	1.339
C16	N15	966	1.339
C16	S49	450	1.779
C2	C3	938	1.4
C2	H8	734	1.08
C21	C22	938	1.4
C21	C26	938	1.4
C21	C41	634	1.51
C22	C23	938	1.4
C22	H27	734	1.08
C23	H28	734	1.08
C23	N24	966	1.339
C25	C26	938	1.4
C25	H29	734	1.08
C25	N24	966	1.339
C26	H30	734	1.08
C3	N4	966	1.339
C3	S54	450	1.779

C31	C32	938	1.4
C31	C36	938	1.4
C31	C45	634	1.51
C32	C33	938	1.4
C32	H37	734	1.08
C33	H38	734	1.08
C33	N34	966	1.339
C35	C36	938	1.4
C35	H39	734	1.08
C35	N34	966	1.339
C36	H40	734	1.08
C41	H42	680	1.09
C41	H43	680	1.09
C41	H44	680	1.09
C45	H46	680	1.09
C45	H47	680	1.09
C45	H48	680	1.09
C5	C6	938	1.4
C5	N4	966	1.339
C6	H9	734	1.08
O20	S54	1470	1.471
O50	S49	1470	1.471
O52	S49	1470	1.471
O53	S49	1470	1.471
O55	S54	1470	1.471
O56	S54	1470	1.471
N15	Ru10	109.748	2.389
N24	Ru10	277.4	2.123
N34	Ru10	277.4	2.142
N4	Ru10	109.748	2.388

O20	Ru10	156.076	2.154
O50	Ru10	156.076	2.15
O51	Ru10	1017.26	1.711

[a] The bond stretch potential energy was described using a harmonic potential: $V_{Harmonic} = 0.5\ k_b(r-r_0)^2$.

Supplementary Table 7. Angle parameters used to describe the atoms in the complex $[Ru^{V}=O]^{+}$ and $Ru^{IV}=O^{a}$.

Angle Bend Parameters Used in the complexes				
Angle types			k_a (kcal $mol^{-1}rad^{-2}$)	Θ_0 (deg)
C1	C2	C3	126	120
C1	C2	H8	70	120
C1	C6	C5	126	120
C1	C6	H9	70	120
C11	C12	C13	126	120
C11	C12	H18	70	120
C11	C16	N15	140	124
C11	C16	S49	130	117.067
C12	C11	C16	126	120
C12	C11	H17	70	120
C12	C13	C14	126	120
C12	C13	H19	70	120
C13	C12	H18	70	120
C13	C14	C5	120	122.76
C13	C14	N15	140	124
C14	C13	H19	70	120
C14	C5	C6	120	122.76
C14	C5	N4	140	116.692
C14	N15	C16	140	117
C16	C11	H17	70	120
C16	S49	O50	200	107.671
C16	S49	O52	200	107.671
C16	S49	O53	200	107.671

C2	C1	C6	126	120
C2	C1	H7	70	120
C2	C3	N4	140	124
C2	C3	S54	130	117.067
C21	C22	C23	126	120
C21	C22	H27	70	120
C21	C26	C25	126	120
C21	C26	H30	70	120
C21	C41	H42	70	109.5
C21	C41	H43	70	109.5
C21	C41	H44	70	109.5
C22	C21	C26	126	120
C22	C21	C41	140	120
C22	C23	H28	70	120
C22	C23	N24	140	124
C23	C22	H27	70	120
C23	N24	C25	140	117
C25	C26	H30	70	120
C26	C21	C41	140	120
C26	C25	H29	70	120
C26	C25	N24	140	124
C3	C2	H8	70	120
C3	N4	C5	140	117
C3	S54	O20	200	107.671
C3	S54	O55	200	107.671
C3	S54	O56	200	107.671
C31	C32	C33	126	120
C31	C32	H37	70	120
C31	C36	C35	126	120
C31	C36	H40	70	120

C31	C45	H46	70	109.5
C31	C45	H47	70	109.5
C31	C45	H48	70	109.5
C32	C31	C36	126	120
C32	C31	C45	140	120
C32	C33	H38	70	120
C32	C33	N34	140	124
C33	C32	H37	70	120
C33	N34	C35	140	117
C35	C36	H40	70	120
C36	C31	C45	140	120
C36	C35	H39	70	120
C36	C35	N34	140	124
C5	C14	N15	140	116.692
C5	C6	H9	70	120
C6	C1	H7	70	120
C6	C5	N4	140	124
H28	C23	N24	70	116
H29	C25	N24	70	116
H38	C33	N34	70	116
H39	C35	N34	70	116
H42	C41	H43	66	107.8
H42	C41	H44	66	107.8
H43	C41	H44	66	107.8
H46	C45	H47	66	107.8
H46	C45	H48	66	107.8
H47	C45	H48	66	107.8
N15	C16	S49	160	113.884
N4	C3	S54	160	113.884
O20	S54	O55	230	120.172

O20	S54	O56	230	120.172
O50	S49	O52	230	120.172
O50	S49	O53	230	120.172
O52	S49	O53	230	120.172
O55	S54	O56	230	120.172
C14	N15	Ru10	235.32	120.689
C16	N15	Ru10	268.88	119.674
C23	N24	Ru10	181.034	121.224
C25	N24	Ru10	181.034	119.488
C3	N4	Ru10	268.88	119.682
C33	N34	Ru10	181.966	120.012
C35	N34	Ru10	181.966	120.818
C5	N4	Ru10	235.32	120.886
N15	Ru10	N24	97.468	93.854
N15	Ru10	N34	113.59	83.912
N15	Ru10	N4	252.92	60.335
N15	Ru10	O20	170.838	134.887
N15	Ru10	O50	355.8	70.581
N15	Ru10	O51	181.838	147.64
N24	Ru10	N34	122.394	177.766
N24	Ru10	N4	84.186	91.222
N24	Ru10	O20	67.344	89.795
N24	Ru10	O50	77.206	89.012
N24	Ru10	O51	125.136	93.161
N34	Ru10	N4	84.186	86.615
N34	Ru10	O20	67.344	88.937
N34	Ru10	O50	77.206	89.23
N34	Ru10	O51	125.136	91.266
N4	Ru10	O20	355.8	69.819
N4	Ru10	O50	170.838	135.914

N4	Ru10	O51	176.552	146.503
O20	Ru10	O50	137.776	154.003
O20	Ru10	O51	188.556	76.724
O50	Ru10	O51	188.556	77.394
Ru10	O20	S54	116.868	124.415
Ru10	O50	S49	106.886	123.196

[a] Angle bend potential energy function: $V_{Angle} = k_a (\Theta-\Theta_0)^2$.

Supplementary Table 8. Torsion parameters used to describe the atoms in the complexes $[Ru^{V}=O]^{+}$ and $Ru^{IV}=O^{a}$.

Torsion Parameters Used in the Complexes						
Dihedral types				V (kcal mol^{-1})	m	Θ_0 (deg)
C1	C2	C3	N4	3.625	2	180
C1	C2	C3	S54	3.625	2	180
C1	C6	C5	C14	3.625	2	180
C1	C6	C5	N4	3.625	2	180
C11	C12	C13	C14	3.625	2	180
C11	C12	C13	H19	3.625	2	180
C11	C16	N15	C14	3.625	2	180
C11	C16	S49	O50	0.12	-2	180
C11	C16	S49	O50	0.195	1	0
C11	C16	S49	O52	0.12	-2	180
C11	C16	S49	O52	0.195	1	0
C11	C16	S49	O53	0.12	-2	180
C11	C16	S49	O53	0.195	1	0
C12	C11	C16	N15	3.625	2	180
C12	C11	C16	S49	3.625	2	180
C12	C13	C14	C5	3.625	2	180
C12	C13	C14	N15	3.625	2	180
C13	C12	C11	C16	3.625	2	180
C13	C12	C11	H17	3.625	2	180
C13	C14	C5	C6	0.0745	-3	0

C13	C14	C5	C6	0.633	-2	180
C13	C14	C5	C6	-0.1945	1	0
C13	C14	C5	N4	0.2275	-3	0
C13	C14	C5	N4	0.5885	-2	180
C13	C14	C5	N4	0.043	1	0
C13	C14	N15	C16	3.625	2	180
C14	C13	C12	H18	3.625	2	180
C14	C5	C6	H9	3.625	2	180
C14	C5	N4	C3	3.625	2	180
C14	N15	C16	S49	3.625	2	180
C16	C11	C12	H18	3.625	2	180
C16	N15	C14	C5	3.625	2	180
C2	C1	C6	C5	3.625	2	180
C2	C1	C6	H9	3.625	2	180
C2	C3	N4	C5	3.625	2	180
C2	C3	S54	O20	0.12	-2	180
C2	C3	S54	O20	0.195	1	0
C2	C3	S54	O55	0.12	-2	180
C2	C3	S54	O55	0.195	1	0
C2	C3	S54	O56	0.12	-2	180
C2	C3	S54	O56	0.195	1	0
C21	C22	C23	H28	3.625	2	180
C21	C22	C23	N24	3.625	2	180
C21	C26	C25	H29	3.625	2	180
C21	C26	C25	N24	3.625	2	180
C22	C21	C26	C25	3.625	2	180
C22	C21	C26	H30	3.625	2	180
C22	C21	C41	H42	0	1	0
C22	C21	C41	H43	0	1	0
C22	C21	C41	H44	0	1	0

C22	C23	N24	C25	3.625	2	180
C23	C22	C21	C26	3.625	2	180
C23	C22	C21	C41	3.625	2	180
C23	N24	C25	C26	3.625	2	180
C23	N24	C25	H29	3.625	2	180
C25	C26	C21	C41	3.625	2	180
C25	N24	C23	H28	3.625	2	180
C26	C21	C22	H27	3.625	2	180
C26	C21	C41	H42	0	1	0
C26	C21	C41	H43	0	1	0
C26	C21	C41	H44	0	1	0
C3	C2	C1	C6	3.625	2	180
C3	C2	C1	H7	3.625	2	180
C3	N4	C5	C6	3.625	2	180
C31	C32	C33	H38	3.625	2	180
C31	C32	C33	N34	3.625	2	180
C31	C36	C35	H39	3.625	2	180
C31	C36	C35	N34	3.625	2	180
C32	C31	C36	C35	3.625	2	180
C32	C31	C36	H40	3.625	2	180
C32	C31	C45	H46	0	1	0
C32	C31	C45	H47	0	1	0
C32	C31	C45	H48	0	1	0
C32	C33	N34	C35	3.625	2	180
C33	C32	C31	C36	3.625	2	180
C33	C32	C31	C45	3.625	2	180
C33	N34	C35	C36	3.625	2	180
C33	N34	C35	H39	3.625	2	180
C35	C36	C31	C45	3.625	2	180
C35	N34	C33	H38	3.625	2	180

C36	C31	C32	H37	3.625	2	180
C36	C31	C45	H46	0	1	0
C36	C31	C45	H47	0	1	0
C36	C31	C45	H48	0	1	0
C41	C21	C22	H27	3.625	2	180
C41	C21	C26	H30	3.625	2	180
C45	C31	C32	H37	3.625	2	180
C45	C31	C36	H40	3.625	2	180
C5	C14	C13	H19	3.625	2	180
C5	C6	C1	H7	3.625	2	180
C5	N4	C3	S54	3.625	2	180
C6	C1	C2	H8	3.625	2	180
C6	C5	C14	N15	0.2275	-3	0
C6	C5	C14	N15	0.5885	-2	180
C6	C5	C14	N15	0.043	1	0
H17	C11	C12	H18	3.625	2	180
H17	C11	C16	N15	3.625	2	180
H17	C11	C16	S49	3.625	2	180
H18	C12	C13	H19	3.625	2	180
H19	C13	C14	N15	3.625	2	180
H27	C22	C23	H28	3.625	2	180
H27	C22	C23	N24	3.625	2	180
H29	C25	C26	H30	3.625	2	180
H30	C26	C25	N24	3.625	2	180
H37	C32	C33	H38	3.625	2	180
H37	C32	C33	N34	3.625	2	180
H39	C35	C36	H40	3.625	2	180
H40	C36	C35	N34	3.625	2	180
H7	C1	C2	H8	3.625	2	180
H7	C1	C6	H9	3.625	2	180

H8	C2	C3	N4	3.625	2	180
H8	C2	C3	S54	3.625	2	180
H9	C6	C5	N4	3.625	2	180
N15	C14	C5	N4	5	2	180
N15	C16	S49	O50	0.431	-3	0
N15	C16	S49	O50	-0.011	-2	180
N15	C16	S49	O50	-0.118	1	0
N15	C16	S49	O52	0.431	-3	0
N15	C16	S49	O52	-0.011	-2	180
N15	C16	S49	O52	-0.118	1	0
N15	C16	S49	O53	0.431	-3	0
N15	C16	S49	O53	-0.011	-2	180
N15	C16	S49	O53	-0.118	1	0
N4	C3	S54	O20	0.431	-3	0
N4	C3	S54	O20	-0.011	-2	180
N4	C3	S54	O20	-0.118	1	0
N4	C3	S54	O55	0.431	-3	0
N4	C3	S54	O55	-0.011	-2	180
N4	C3	S54	O55	-0.118	1	0
N4	C3	S54	O56	0.431	-3	0
N4	C3	S54	O56	-0.011	-2	180
N4	C3	S54	O56	-0.118	1	0
C11	C16	N15	Ru10	0	2	166.615
C13	C14	N15	Ru10	0	2	-165.766
C14	C5	N4	Ru10	0	2	-15.448
C14	N15	Ru10	N24	0	2	-109.539
C14	N15	Ru10	N34	0	2	70.477
C14	N15	Ru10	N4	0	2	-18.586
C14	N15	Ru10	O20	0	2	-11.905
C14	N15	Ru10	O50	0	2	161.81

C14	N15	Ru10	O51	0	2	153.191
C16	N15	Ru10	N24	0	2	81.395
C16	N15	Ru10	N34	0	2	-98.59
C16	N15	Ru10	N4	0	2	172.348
C16	N15	Ru10	O20	0	2	179.028
C16	N15	Ru10	O50	0	2	-7.256
C16	N15	Ru10	O51	0	2	-15.876
C16	S49	O50	Ru10	0	2	-47.321
C2	C3	N4	Ru10	0	2	-169.88
C22	C23	N24	Ru10	0	2	-177.428
C23	N24	Ru10	N15	0.575	2	44.964
C23	N24	Ru10	N34	0.575	2	45.133
C23	N24	Ru10	N4	0.575	2	-20.382
C23	N24	Ru10	O20	0.575	2	-90.231
C23	N24	Ru10	O50	0.575	2	115.524
C23	N24	Ru10	O51	0.575	2	-167.15
C25	N24	Ru10	N15	0.575	2	-131.814
C25	N24	Ru10	N34	0.575	2	-131.645
C25	N24	Ru10	N4	0.575	2	162.84
C25	N24	Ru10	O20	0.575	2	92.991
C25	N24	Ru10	O50	0.575	2	-61.254
C25	N24	Ru10	O51	0.575	2	16.062
C26	C25	N24	Ru10	0	2	178.121
C3	N4	Ru10	N15	0	2	-171.624
C3	N4	Ru10	N24	0	2	-81.245
C3	N4	Ru10	N34	0	2	103.526
C3	N4	Ru10	O20	0	2	13.414
C3	N4	Ru10	O50	0	2	-171.087
C3	N4	Ru10	O51	0	2	16.35
C3	S54	O20	Ru10	0	2	51.809

C32	C33	N34	Ru10	0	2	178.349
C33	N34	Ru10	N15	0.575	2	80.92
C33	N34	Ru10	N24	0.575	2	80.751
C33	N34	Ru10	N4	0.575	2	146.457
C33	N34	Ru10	O20	0.575	2	-143.697
C33	N34	Ru10	O50	0.575	2	10.369
C33	N34	Ru10	O51	0.575	2	-67.003
C35	N34	Ru10	N15	0.575	2	80.92
C35	N34	Ru10	N24	0.575	2	-101.432
C35	N34	Ru10	N4	0.575	2	-35.725
C35	N34	Ru10	O20	0.575	2	34.12
C35	N34	Ru10	O50	0.575	2	-171.813
C35	N34	Ru10	O51	0.575	2	110.814
C36	C35	N34	Ru10	0	2	-178.537
C5	C14	N15	Ru10	0	2	17.932
C5	N4	Ru10	N15	0	2	17.653
C5	N4	Ru10	N24	0	2	108.031
C5	N4	Ru10	N34	0	2	-67.198
C5	N4	Ru10	O20	0	2	-157.309
C5	N4	Ru10	O50	0	2	18.19
C5	N4	Ru10	O51	0	2	-154.373
C6	C5	N4	Ru10	0	2	168.496
H28	C23	N24	Ru10	0	2	2.878
H29	C25	N24	Ru10	0	2	1.608
H38	C33	N34	Ru10	0	2	-1.364
H39	C35	N34	Ru10	0	2	-0.546
N15	Ru10	O20	S54	0	2	-47.14
N15	Ru10	O50	S49	0	2	35.007
N24	Ru10	O20	S54	0	2	48.867
N24	Ru10	O50	S49	0	2	-56.247

N34	Ru10	O20	S54	0	2	-127.457
N34	Ru10	O50	S49	0	2	118.823
N4	Ru10	O20	S54	0	2	-40.673
N4	Ru10	O50	S49	0	2	34.49
O20	Ru10	O50	S49	0	2	-155.185
O50	Ru10	O20	S54	0	2	146.483
O51	Ru10	O20	S54	0	2	140.991
O51	Ru10	O50	S49	0	2	-149.708
O52	S49	O50	Ru10	0	2	-160.72
O53	S49	O50	Ru10	0	2	63.012
O55	S54	O20	Ru10	0	2	163.774
O56	S54	O20	Ru10	0	2	-59.15
Ru10	N15	C16	S49	0	2	-14.818
Ru10	N4	C3	S54	0	2	9.615

[a] Torsion potential energy function: $V_{Torsion} = \Sigma_i Vi \bullet (1+\cos(m\Phi - \Phi oi))$

Supplementary Table 9. Improper torsion parameters used to describe the atoms in the complexes $[Ru^{V}=O]^{+}$ and $Ru^{IV}=O^{a}$.

Improper Torsion Parameters Used in the Complexes					
Improper torsion type				k_ξ (kcal mol^{-1}rad $^{-2}$)	ξ_0 (deg)
C1	C2	C3	H8	1.1	180
C1	C6	C5	H9	1.1	180
C11	C12	C13	H18	1.1	180
C11	C16	N15	S49	4	180
C12	C11	C16	H17	1.1	180
C12	C13	C14	H19	1.1	180
C13	C14	C5	N15	4	180
C14	C5	C6	N4	4	180
C2	C1	C6	H7	1.1	180
C2	C3	N4	S54	4	180
C21	C22	C23	H27	1.1	180
C21	C26	C25	H30	1.1	180

C22	C21	C26	C41	4	180
C22	C23	H28	N24	1.1	180
C26	C25	H29	N24	1.1	180
C31	C32	C33	H37	1.1	180
C31	C36	C35	H40	1.1	180
C32	C31	C36	C45	4	180
C32	C33	H38	N34	1.1	180
C36	C35	H39	N34	1.1	180
C14	N15	C16	Ru10	2.5	180
C23	N24	C25	Ru10	2.5	180
C3	N4	C5	Ru10	2.5	180
C33	N34	C35	Ru10	2.5	180

[a] Improper torsion potential energy function: $V_{Improper} = k_{\xi} (\xi-\xi_0)^2$.

Cartesian Coordinates in Å and Energies of the Calculated Geometries.
[Ru^{II}]

E (B3LYP-D3/LACVP**++ 2f(Ru))(a.u.) = -2793.405043
ZPE (kcal mol^{-1}) = 334.123
G_{solv} (kcal mol^{-1}) = -43.876
ΔH_{298} (kcal mol^{-1}) = 28.935
ΔS_{298} (cal K^{-1} mol^{-1}) = 265.623
Cartesian coordinates

Atom	x	y	z
C1	2.4994071400	2.7990328090	2.3042638200
C2	1.3880528939	3.2912847086	1.6091509973
C3	0.1435352019	3.1824268396	2.2135482887
N4	-0.0085028523	2.5935033155	3.4064554636
C5	1.0640559748	2.1305381263	4.1224078753
C6	2.3427542781	2.2295987506	3.5711214669
H7	3.4886958146	2.8677236537	1.8635738512
H8	1.4698320397	3.7478913462	0.6292762368
H9	3.2041059530	1.8673391576	4.1216852705
Ru10	-1.7858198243	2.3345062514	4.2364954994
C11	-0.3629962452	0.7210018649	7.8720070205
C12	1.0122783826	0.6330062460	7.6354486066
C13	1.5477536380	1.0804437770	6.4241823776
C14	0.6953938583	1.5989014780	5.4497780350
N15	-0.6500261558	1.6590670484	5.7072649835
C16	-1.1660016007	1.2563101981	6.8729519574
H17	-0.8127051804	0.3841931808	8.7992099113
H18	1.6691667612	0.2212457300	8.3948830549
H19	2.6151640046	1.0238740751	6.2414753171
O20	-2.4757001281	3.0482405228	2.2439282253
C21	-2.0595415616	6.8685910601	6.0940514962
C22	-0.8157214719	6.2402434993	5.9583868428
C23	-0.7450247267	4.9566966718	5.4414935968
N24	-1.8387919030	4.2688697637	5.0633713504
C25	-3.0478923905	4.8516172004	5.2023748628

C26	-3.1849228757	6.1396549684	5.6988363774
H27	0.0990981286	6.7522427482	6.2415029589
H28	0.2102135616	4.4601124406	5.3129066033
H29	-3.9035302263	4.2750008062	4.8679642094
H30	-4.1774990189	6.5728774485	5.7560041279
C31	-2.9323492066	-2.1645823489	2.6314307810
C32	-1.7074560066	-1.9675871523	3.2913853925
C33	-1.3205175387	-0.6895593035	3.6688774067
N34	-2.0803527313	0.3984496348	3.4218270051
C35	-3.2396472882	0.2259791471	2.7551453828
C36	-3.6871538803	-1.0242370786	2.3501277354
H37	-1.0662484061	-2.8107093108	3.5309735045
H38	-0.3880706214	-0.5235979530	4.1975211419
H39	-3.8604075436	1.0874600769	2.5486433666
H40	-4.6473952209	-1.0687964127	1.8474493507
C41	-2.1838905378	8.2603499465	6.6551425934
H42	-2.4804555338	8.2205333604	7.7101105897
H43	-1.2378147738	8.8055215331	6.5948311117
H44	-2.9489676016	8.8325948550	6.1220965114
C45	-3.4379987157	-3.5448234789	2.3127682337
H46	-4.0797417243	-3.5385924806	1.4281315673
H47	-2.6186214473	-4.2516152337	2.1517386980
H48	-4.0387660088	-3.9130078063	3.1537280686
S49	-2.9685362926	1.5560802918	7.0492253517
O50	-3.4254694156	1.7741228188	5.6068109227
O51	-5.9613360143	1.2690850919	1.9186459225
H52	-5.9584256432	2.2322633066	2.0470466783
H53	-6.0991790970	0.9467742080	2.8351588481
O54	-3.4990483356	0.2954283549	7.6094571197
O55	-3.0657781496	2.7566340541	7.8837281051
S56	-1.4071040126	3.8942082967	1.5494670763
O57	-1.3635339651	3.7151444916	0.0986866943
O58	-1.4041731499	5.2850799225	2.0644635450
O59	-4.3428742597	-1.5131324624	5.6195669532
H60	-3.5234430194	-1.4262494476	5.1178680923
H61	-4.1618253582	-1.0041259569	6.4328586738
O62	-5.8986811785	0.6075495102	4.5953783910
H63	-5.4503619871	-0.2122080387	4.8833844397
H64	-5.3120040332	1.3076740568	4.9158209874
O65	-5.0911157597	3.7733880968	2.9973740617
H66	-4.9909436839	4.7371161595	2.8695315444
H67	-4.2633420464	3.4472660481	2.6079228699
O68	-4.0113827797	6.3315528452	2.5612591237
H69	-3.0871356701	6.0213779468	2.4926689243
H70	-4.2183830078	6.6367398108	1.6689579075

[Ru^{III}(k_3^{O,N,N}-bds)(pic)$_2$(OH$_2$)]$^+$ 6-coordination
E (B3LYP-D3/LACVP**++ 2f(Ru))(a.u.) = -2793.17931
ZPE (kcal mol^{-1}) = 335.139
G_{solv} (kcal mol^{-1}) = -65.111
ΔH_{298} (kcal mol^{-1}) = 27.59
ΔS_{298} (cal K^{-1} mol^{-1}) = 253.002
Cartesian coordinates

Atom	x	y	z
C1	2.2682300602	3.2167494743	2.1266066483
C2	1.1167704102	3.7735543531	1.5611684028
C3	-0.0904664556	3.5109337764	2.1873365274
N4	-0.1780211426	2.7604993299	3.2920901700
C5	0.9140473480	2.1787339621	3.8331382359
C6	2.1691073132	2.4100419840	3.2590553133
H7	3.2384247308	3.3983053664	1.6756323580
H8	1.1420484559	4.3718031799	0.6573436595
H9	3.0594165985	1.9648205616	3.6851602167
Ru10	-2.0433469553	2.3170845543	4.0422121040

C11	-0.0087006403	0.0515943546	7.3547285085
C12	1.2870880633	-0.0459535485	6.8639028574
C13	1.6159185145	0.6442442168	5.6997963824
C14	0.6306748473	1.3634490434	5.0263223007
N15	-0.6648347155	1.3862485017	5.4695400556
C16	-0.9537767045	0.7837216236	6.6351519084
H17	-0.3140734460	-0.3934958248	8.2948168735
H18	2.0408216996	-0.6190953973	7.3940132242
H19	2.6312538801	0.6267641782	5.3236280352
O20	-2.6090222671	2.9661875692	2.1965985670
C21	-2.4101771723	6.6779321974	6.2454421829
C22	-1.2543188719	5.8934045549	6.3756224438
C23	-1.1634522075	4.6859891875	5.7090352638
N24	-2.1511259363	4.2271398533	4.9131614716
C25	-3.2655753894	4.9714966244	4.7615839599
C26	-3.4201891756	6.1870275610	5.4121143338
H27	-0.4332466079	6.2161021537	7.0079609060
H28	-0.2930879225	4.0505471967	5.8171009609
H29	-4.0393671303	4.5748738147	4.1141467611
H30	-4.3398726623	6.7441364388	5.2686277436
C31	-2.1175591723	-2.1669982032	1.9523259597
C32	-2.6878895172	-1.9228704315	3.2078663912
C33	-2.6426230988	-0.6531302174	3.7687224174
N34	-2.0456466266	0.3778520966	3.1341358324
C35	-1.5073993401	0.1680457181	1.9154801972
C36	-1.5228871260	-1.0755850884	1.3044770507
H37	-3.1800999934	-2.7195513637	3.7567773123
H38	-3.0903166060	-0.4327437045	4.7326145793
H39	-1.0806145106	1.0267809801	1.4129876286
H40	-1.0810736317	-1.1873838718	0.3193385168
C41	-2.5581187297	7.9695189130	7.0009519744
H42	-2.8583927201	7.7627980165	8.0352739795
H43	-1.6122487244	8.5178343058	7.0405866464
H44	-3.3189346506	8.6144405966	6.5555030693
C45	-2.1269070853	-3.5385280523	1.3338502479
H46	-1.2720123070	-4.1221177853	1.6974605461
H47	-3.0339512989	-4.0887671351	1.5981062752
H48	-2.0529775753	-3.4884477244	0.2444351897
S49	-2.5910194662	1.0691476154	7.4069334564
O50	-2.7859463400	2.5126206746	7.0782074561
O51	-4.0589928162	1.9252292099	4.3727833576
H52	-4.5672921173	2.3121391203	3.6000990297
H53	-4.5099071434	2.3937341924	5.1772079848
O54	-3.5180738184	0.1516860272	6.6861229893
O55	-2.3813334893	0.7605247467	8.8252306741
S56	-1.7009825326	4.0657591714	1.5479932226
O57	-1.6665900788	3.8837393819	0.0965106118
O58	-1.9674409394	5.4043148497	2.0812647180
O59	-6.1699036864	0.8300502768	7.2089177312
H60	-6.3319590677	0.7083879774	8.1526163292
H61	-5.3107073381	0.3959290438	7.0358135419
O62	-5.2794109983	3.1413332170	6.2374600232
H63	-5.7987004623	2.3986219584	6.6426243245
H64	-4.5092398505	3.2175714570	6.8304588185
O65	-5.4146720265	3.3446427784	2.6779699889
H66	-6.3647391879	3.4873175661	2.7500000259
H67	-5.1003663191	3.8074497153	1.8670780939
O68	-4.5979014406	4.8858706551	0.6016592686
H69	-4.0377158090	5.5837838572	0.9719475985
H70	-4.0587892698	4.5012988672	-0.1043932761

[Ru^{III}(bds)(pic)$_2$(OH$_2$)]$^+$ 7-coordination
E (B3LYP-D3/LACVP**++ 2f(Ru))(a.u.) = -2793.173227
ZPE (kcal mol^{-1}) = 334.721

G_{solv} (kcal mol^{-1}) = -59.722
ΔH_{298} (kcal mol^{-1}) = 28.43
ΔS_{298} (cal K^{-1} mol^{-1}) = 262.694
Cartesian coordinates

Atom	x	y	z
C1	2.9570960617	2.3550766933	2.0240972938
C2	1.8738185304	3.0122997509	1.4383519261
C3	0.6994550497	3.0696990689	2.1810150910
N4	0.5602863059	2.5335250546	3.3834551067
C5	1.6084391852	1.9473569059	3.9755956941
C6	2.8354534605	1.8298899896	3.3120140573
H7	3.8969894728	2.2643639663	1.4886755382
H8	1.9218146123	3.4605396142	0.4524917123
H9	3.6797012514	1.3375636449	3.7807612328
Ru10	-1.8122770980	2.1993176047	4.3213618162
C11	0.6136924373	0.7941937515	7.9277828212
C12	1.9490961125	0.6949873213	7.5428029390
C13	2.3084982753	1.0533764297	6.2451834826
C14	1.3287580610	1.5113313148	5.3585419872
N15	0.0358989932	1.6198400499	5.7387541391
C16	-0.2902540452	1.2615250616	6.9813037002
H17	0.2641865275	0.5044948498	8.9122201530
H18	2.6998337041	0.3371238906	8.2402966222
H19	3.3420965215	0.9835398900	5.9271625150
O20	-1.8457785739	3.1799410098	2.4172805277
C21	-2.0004895536	6.6760536929	6.3695267956
C22	-0.8226273808	6.1610843205	5.8139640042
C23	-0.8149293284	4.8899805967	5.2665865957
N24	-1.9095204642	4.1011810210	5.2486874225
C25	-3.0493712873	4.5760820313	5.7905192587
C26	-3.1251495308	5.8457204631	6.3441674219
H27	0.0873801364	6.7522069291	5.7913321450
H28	0.0779414815	4.4864181130	4.8105443142
H29	-3.9055397642	3.9156843169	5.7964272939
H30	-4.0693453281	6.1793383901	6.7617289666
C31	-1.4320904289	-2.1132969296	1.9365809616
C32	-1.1687359504	-2.0168628050	3.3101394575
C33	-1.3120876764	-0.8007877145	3.9612115175
N34	-1.6964649226	0.3204691335	3.3188070687
C35	-1.9812220840	0.2417734560	2.0033976344
C36	-1.8572372903	-0.9451406828	1.2928695754
H37	-0.8551035079	-2.8886712873	3.8761874318
H38	-1.1333049863	-0.7062046642	5.0248245779
H39	-2.3016688948	1.1619955754	1.5310333078
H40	-2.0947745140	-0.9518654558	0.2336464459
C41	-2.0374458247	8.0467597498	6.9890093818
H42	-1.7298071134	7.9923778869	8.0402921554
H43	-1.3523106497	8.7323027346	6.4821306292
H44	-3.0431257360	8.4737950704	6.9612518325
C45	-1.2443252677	-3.4061387703	1.1895845168
H46	-0.2063918047	-3.4951423195	0.8452153654
H47	-1.4527700224	-4.2703525185	1.8262775456
H48	-1.8884640269	-3.4576983961	0.3080932188
S49	-2.0532922420	1.3735812591	7.3533754266
O50	-2.5862938951	1.1380051679	5.9037949486
O51	-3.7835449321	2.2489441271	3.7542503736
H52	-4.0229982826	3.1741436607	3.3854823260
H53	-4.4071276818	2.1090110699	4.5433873962
O54	-2.3865160090	0.2013317482	8.1742068547
O55	-2.3415898674	2.7056787559	7.8747823418
S56	-0.7668559139	4.0001742003	1.7006474452
O57	-0.9377089102	3.9261768570	0.2452104763
O58	-0.5767565777	5.3402220450	2.2913138407
O59	-4.4878912150	4.5895949670	3.0301675814

H60	-5.4060600981	4.7061754524	2.7581165947
H61	-3.9461509230	5.0584280602	2.3398928207
O62	-3.1555413099	5.9868250623	1.1882110202
H63	-2.3109432613	6.2376675766	1.6001701264
H64	-2.8776173242	5.5009554839	0.3974661290
O65	-5.4082189397	2.1456778572	5.7514719399
H66	-6.3490997303	2.1829034738	5.5396955312
H67	-5.2832919755	1.3334302457	6.3123218896
O68	-4.9971781399	-0.1617258118	7.0088625711
H69	-4.2591667674	-0.4556590767	6.4560601379
H70	-4.5839858210	-0.1005667446	7.8840123800

[Ru^{IV}(*k$_3$*^{O,N,N}-bds)(pic)$_2$(OH)]$^+$ 6-coordinate

E (B3LYP-D3/LACVP**++ 2f(Ru))(a.u.) = -2792.48665

ZPE (kcal mol^{-1}) = 327.726

G_{solv} (kcal mol^{-1}) = -67.475

ΔH_{298} (kcal mol^{-1}) = 27.537

ΔS_{298} (cal K^{-1} mol^{-1}) = 253.032

Cartesian coordinates

Atom	x	y	z
C1	2.2083653578	2.9026810188	2.0291203744
C2	1.0852364943	3.4804279512	1.4325161846
C3	-0.1343045341	3.3076767416	2.0712725991
N4	-0.2456175327	2.6105796465	3.2043048952
C5	0.8247245142	2.0574069192	3.8146152568
C6	2.0856220581	2.1966364738	3.2281115476
H7	3.1840587117	3.0060673914	1.5647063859
H8	1.1385509140	4.0349303052	0.5024803230
H9	2.9651186800	1.7706304085	3.6944546063
Ru10	-2.1151773051	2.3038640948	4.0524226036
C11	-0.0985159214	0.4606797249	7.6155300827
C12	1.1923511871	0.2952255773	7.1377360325
C13	1.5091182437	0.7897690529	5.8723692370
C14	0.5255190315	1.4132009468	5.1113854106
N15	-0.7663962294	1.5239094274	5.5676018107
C16	-1.0535566227	1.0766166731	6.7996254410
H17	-0.4037353592	0.1397277269	8.6048064086
H18	1.9507818479	-0.1925323977	7.7418325496
H19	2.5181423733	0.6972083605	5.4891778094
O20	-2.7181511046	3.0793175325	2.1755621395
C21	-2.1445431927	6.7600886950	6.1144765093
C22	-0.9961894140	5.9566166351	6.1568958125
C23	-0.9987547819	4.7124472265	5.5522193363
N24	-2.0774456652	4.2281266131	4.9041159614
C25	-3.1893649332	4.9860876494	4.8502846354
C26	-3.2514910053	6.2418800187	5.4338485788
H27	-0.0999935645	6.2973794581	6.6657049917
H28	-0.1204506400	4.0805731661	5.5856464108
H29	-4.0383569907	4.5678613396	4.3335246398
H30	-4.1714768259	6.8098413474	5.3469064612
C31	-1.9031413086	-2.2320787358	2.0416955260
C32	-2.2739821582	-2.0209249071	3.3764816639
C33	-2.3218919319	-0.7358658334	3.8973310099
N34	-2.0032571210	0.3428032340	3.1504809345
C35	-1.6782211610	0.1655765806	1.8548744733
C36	-1.6139318533	-1.0939343451	1.2778674110
H37	-2.5411462224	-2.8563114922	4.0162135630
H38	-2.6444770022	-0.5435546122	4.9156437859
H39	-1.4977392889	1.0554152190	1.2659610737
H40	-1.3516187501	-1.1808821481	0.2282503318
C41	-2.1847358644	8.0992581134	6.7967272034
H42	-2.4789043649	7.9731803600	7.8460914126
H43	-1.2038370394	8.5827289514	6.7871955558
H44	-2.9107327223	8.7679117538	6.3277897311

C45	-1.8144204735	-3.6173258002	1.4620190472
H46	-0.8649112763	-4.0861202987	1.7485461670
H47	-2.6184857349	-4.2562167218	1.8381787926
H48	-1.8620219892	-3.6018297953	0.3705253234
S49	-2.7534226253	1.2312771747	7.4721262027
O50	-3.1587927593	2.6075222267	7.0629726790
O51	-3.9060126615	1.8384201352	4.2136374454
H52	-4.5454511168	2.4590752009	3.6499007121
O53	-3.4636096598	0.1443048262	6.7368390743
O54	-2.5882844642	1.0209122498	8.9125052629
S55	-1.7082283676	3.9907214893	1.4507574518
O56	-1.7123642362	3.7658546837	0.0096098160
O57	-1.7710467769	5.3779155133	1.9501452584
O58	-6.1142471285	0.2106353263	6.0776848291
H59	-6.6618723493	0.0491650200	6.8564331524
H60	-5.1983335893	-0.0028554308	6.3513368908
O61	-5.4214594541	2.7059551253	5.6163359120
H62	-5.7987962673	1.7909940439	5.7670661169
H63	-4.6727143612	2.7686813971	6.2531066663
O64	-5.5052483300	3.2637474929	2.9000630700
H65	-6.1193161249	3.5131775600	3.6058783837
H66	-5.1540812283	4.1055978431	2.5201275726
O67	-4.5973462240	5.7270705586	2.2029916850
H68	-3.6279496690	5.7869647133	2.1124075292
H69	-4.9579103770	6.1166721709	1.3963708312

$[Ru^{IV}(bds)(pic)_2(OH)]^+$ 7-coordinate S_0
E (B3LYP-D3/LACVP**++ 2f(Ru))(a.u.) = -2792.514287
ZPE (kcal mol^{-1}) = 328.112
G_{solv} (kcal mol^{-1}) = -65.201
ΔH_{298} (kcal mol^{-1}) = 27.431
ΔS_{298} (cal K^{-1} mol^{-1}) = 248.014
Cartesian coordinates

Atom	x	y	z
C1	2.7874329975	4.2094295006	2.7422503524
C2	1.6053988669	4.4174403104	2.0357046104
C3	0.4608142037	3.7640273453	2.4781648352
N4	0.4368557828	2.9695381887	3.5489200614
C5	1.5972251816	2.6979231797	4.1907852523
C6	2.7897521805	3.3180944713	3.8146837910
H7	3.7046705944	4.7055069024	2.4419062098
H8	1.5588773034	5.0375895482	1.1474825827
H9	3.7087210729	3.1033816408	4.3471076908
Ru10	-1.5604634407	2.1583381420	4.2730318256
C11	0.8964290011	-0.0405361922	7.3184872660
C12	2.2253537088	0.2542427645	7.0195027755
C13	2.5077353474	1.1562521933	5.9950851086
C14	1.4571699437	1.7053162259	5.2566554596
N15	0.1740702593	1.3543638850	5.5049521272
C16	-0.0888084569	0.5476666924	6.5358535666
H17	0.6119612827	-0.6741854089	8.1511545674
H18	3.0312326350	-0.1843512046	7.5990385479
H19	3.5316747629	1.4359977808	5.7777419068
O20	-1.6792001983	2.4789255957	2.1720841055
C21	-2.0415467105	6.5865818728	6.4021989559
C22	-1.0231041150	5.6921412339	6.7704869943
C23	-0.8852537901	4.4905531060	6.1029577832
N24	-1.6848327853	4.1438795756	5.0699450676
C25	-2.6510599810	4.9987154571	4.6915459479
C26	-2.8565031505	6.2126288331	5.3322084419
H27	-0.3574522095	5.9206942311	7.5969059073
H28	-0.1355247401	3.7681043888	6.4006034875
H29	-3.2634495537	4.6994901341	3.8582935003
H30	-3.6822519835	6.8293254040	4.9955113375

C31	-1.4841147145	-2.2808084003	2.1118238738
C32	-2.6251652655	-1.8232237266	2.7781825052
C33	-2.6118691348	-0.6009476976	3.4374930907
N34	-1.5148091767	0.1764268527	3.4604800329
C35	-0.4082661828	-0.2404785242	2.8136436478
C36	-0.3544224590	-1.4494096680	2.1430944844
H37	-3.5360372327	-2.4126881188	2.7864450627
H38	-3.4800564794	-0.2174560185	3.9542926339
H39	0.4432028391	0.4288764076	2.8251025063
H40	0.5598689477	-1.7351780837	1.6325750753
C41	-2.2629001600	7.8632642215	7.1639527016
H42	-2.8439050863	7.6526157224	8.0702101495
H43	-1.3160606942	8.3112668646	7.4799494185
H44	-2.8186546591	8.5950257420	6.5733508807
C45	-1.4555398871	-3.6090124324	1.4065629563
H46	-1.1045124144	-4.3908813744	2.0913624634
H47	-2.4498351403	-3.8985445733	1.0575810954
H48	-0.7753754807	-3.5916843599	0.5504433891
S49	-1.8478509961	0.4538372217	6.9407636820
O50	-2.2210676676	1.8017168657	6.2549227999
O51	-3.3931252259	2.1031431760	4.0100545909
H52	-3.9788626023	2.6094387650	4.7372459384
O53	-2.4230848633	-0.7190024279	6.2914097699
O54	-1.9542159657	0.6144149862	8.3897915926
S55	-1.0745316939	3.7450267911	1.5265736918
O56	-0.6902154073	3.5057411817	0.1404796845
O57	-1.8372410771	4.9621417630	1.8465652511
O58	-3.5096253269	3.7274254002	7.8860364677
H59	-3.9335306090	3.3713101184	8.6783439546
H60	-2.8925721791	3.0321399928	7.6031907773
O61	-4.9323917438	3.2177865081	5.6192778416
H62	-5.3183940195	4.0251857635	5.1986478903
H63	-4.5260125741	3.4874426652	6.4741242464
O64	-4.5639557859	3.9439932657	2.0947222370
H65	-3.8146903072	4.4253733504	1.7085295707
H66	-4.1760753552	3.1134719283	2.4215532823
O67	-5.6952035907	5.3244083537	4.1350157235
H68	-5.4148021448	4.8846573680	3.2968288046
H69	-6.6356124378	5.5173784261	4.0372785790

[Ru^{IV}(bds)(pic)$_2$(OH)]$^+$ 7-coordinate T_1
E (B3LYP-D3/LACVP**++ 2f(Ru))(a.u.) = -2792.525894
ZPE (kcal mol^{-1}) = 327.406
G_{solv} (kcal mol^{-1}) = -61.086
ΔH_{298} (kcal mol^{-1}) = 27.421
ΔS_{298} (cal K^{-1} mol^{-1}) = 253.888
Cartesian coordinates

Atom	x	y	z
C1	2.9334872525	4.1835533448	2.7710478765
C2	1.7607446457	4.4237845940	2.0581838471
C3	0.6149792878	3.7527269286	2.4794527192
N4	0.5814504915	2.9144766443	3.5073217173
C5	1.7175475782	2.6538505268	4.1803471564
C6	2.9185539849	3.2826552706	3.8376825899
H7	3.8566897262	4.6822610154	2.4929878307
H8	1.7271986074	5.0838414837	1.1989129835
H9	3.8276915843	3.0827418766	4.3924690831
Ru10	-1.8481432732	2.1633256648	4.1653165242
C11	1.0040379328	0.0006522114	7.4163203893
C12	2.3316612269	0.2581389865	7.0763329333
C13	2.6206116858	1.1137002334	6.0106633459
C14	1.5642354798	1.6819179060	5.2911016942
N15	0.2870147854	1.3939993073	5.5961197791
C16	0.0262567859	0.6057268710	6.6286990680

H17	0.7297368686	-0.6241034148	8.2586177463
H18	3.1394981790	-0.1904974379	7.6459997948
H19	3.6504668961	1.3296453726	5.7502593411
O20	-1.6880935244	2.6430440032	2.1246374442
C21	-1.8405864020	6.5750277524	6.4337923544
C22	-0.9425254168	5.5542243547	6.7788631303
C23	-0.9615282560	4.3520169114	6.0951390843
N24	-1.8049072519	4.1208036012	5.0647766395
C25	-2.6628155176	5.0948703155	4.7127449701
C26	-2.7135941079	6.3133984278	5.3742285802
H27	-0.2378055203	5.6880452338	7.5933143607
H28	-0.3016525475	3.5401860199	6.3665551496
H29	-3.3037121157	4.8837930714	3.8749703401
H30	-3.4419173260	7.0496160878	5.0516423000
C31	-1.4174840307	-2.3281993635	2.2252562965
C32	-2.5078908490	-1.9974656545	3.0377399401
C33	-2.6047734889	-0.7312021467	3.5968633024
N34	-1.6710021163	0.2124344369	3.3741945901
C35	-0.6221018901	-0.0771484111	2.5752690632
C36	-0.4626192757	-1.3256562384	2.0008370434
H37	-3.2867688567	-2.7236966823	3.2445657591
H38	-3.4269795705	-0.4513467257	4.2403892438
H39	0.0787997443	0.7261691096	2.3929315445
H40	0.3975640250	-1.5104326409	1.3651947436
C41	-1.8732520617	7.8709123392	7.1958965473
H42	-2.4370804802	7.7423288131	8.1279039609
H43	-0.8651152205	8.1965932327	7.4690673369
H44	-2.3538929748	8.6661301921	6.6215366799
C45	-1.2571418564	-3.7000944949	1.6298336785
H46	-0.6259589997	-4.3190818973	2.2794447794
H47	-2.2187634900	-4.2074787376	1.5219451503
H48	-0.7728965116	-3.6542010880	0.6502516899
S49	-1.7417091725	0.4120189799	6.9716941570
O50	-2.3038946306	1.6256124811	6.1650815309
O51	-3.6027379918	2.1937822957	3.9088960699
H52	-4.6828249807	2.5234693698	5.0425909536
O53	-2.1665539371	-0.8741710413	6.4187865236
O54	-1.9473891986	0.6879800723	8.3965004699
S55	-0.9501502947	3.8859238729	1.5724664978
O56	-0.6253181632	3.7135622430	0.1602387360
O57	-1.6252789728	5.1335881500	1.9858752394
O58	-3.7654622796	3.3896269849	7.6167007432
H59	-4.0342941966	3.0167056530	8.4680309309
H60	-3.0669305931	2.7842618454	7.2827864725
O61	-5.3009317843	3.0458043155	5.6421986208
H62	-5.5496947688	3.8997719508	5.0828221785
H63	-4.7628652060	3.2536256023	6.5026899519
O64	-4.4173506884	4.2272539189	2.0125881984
H65	-3.6469740336	4.7558593243	1.7385652138
H66	-4.0383162599	3.3600664494	2.2408951594
O67	-5.7696387406	4.9888652260	4.1414570256
H68	-5.3331485691	4.7590487643	3.2686631733
H69	-6.6932874900	5.2001611310	3.9544622422

[Ru^{IV}(bds)(pic)$_2$(OH)]$^+$ S_0

E (PBE/LACVP**++ 2f(Ru))(a.u.) = -2789.92884

ZPE (kcal mol^{-1}) = 316.637

G_{solv} (kcal mol^{-1}) = -61.143

ΔH_{298} (kcal mol^{-1}) = 28.235

ΔS_{298} (cal K^{-1} mol^{-1}) = 257.3

Cartesian coordinates

Atom	x	y	z
C1	2.8300484090	4.1694063786	2.6642811080
C2	1.6302091241	4.4022349775	1.9796941468

C3	0.4722280596	3.7843620300	2.4515687845
N4	0.4423562111	2.9939313619	3.5415479988
C5	1.6223292178	2.6999146927	4.1637350103
C6	2.8290606828	3.2868491480	3.7478550309
H7	3.7604461531	4.6390017236	2.3341664717
H8	1.5802868028	5.0147516035	1.0759619674
H9	3.7599821666	3.0455326710	4.2651309331
Ru10	-1.5144532146	2.1939147947	4.2702653017
C11	0.9103693219	0.0129387386	7.3419470342
C12	2.2484275778	0.3079743842	7.0471165065
C13	2.5315548175	1.1974356776	6.0070385671
C14	1.4792517932	1.7331031802	5.2454556818
N15	0.1810015020	1.3835451249	5.4893940016
C16	-0.0790136952	0.5854816020	6.5438224497
H17	0.6207076078	-0.6135778438	8.1892908049
H18	3.0578013272	-0.1219914423	7.6431358567
H19	3.5625614430	1.4844884988	5.7889882854
O20	-1.6423437305	2.4666504990	2.1333012105
C21	-2.0042384742	6.6243801705	6.4780807684
C22	-0.9716339219	5.7266989421	6.8174406296
C23	-0.8361350916	4.5243250448	6.1312848423
N24	-1.6539805385	4.1768267095	5.1053836087
C25	-2.6431184350	5.0322289107	4.7581651251
C26	-2.8452241548	6.2435910232	5.4193544966
H27	-0.2779166232	5.9558171872	7.6317159933
H28	-0.0628823318	3.8039247754	6.4046880441
H29	-3.2722640247	4.7327478970	3.9221600559
H30	-3.6831496718	6.8688410578	5.0996912658
C31	-1.5752643198	-2.2942525495	2.1559117281
C32	-2.7186102496	-1.7563592276	2.7726375168
C33	-2.6652178608	-0.5156772364	3.4070931522
N34	-1.5253703104	0.2105346474	3.4528419259
C35	-0.4166591690	-0.2791420468	2.8492093946
C36	-0.4042367411	-1.5126546987	2.2052127559
H37	-3.6663117122	-2.3017389377	2.7630490961
H38	-3.5366291058	-0.0624127436	3.8805457320
H39	0.4731805451	0.3532066730	2.8753128805
H40	0.5186865029	-1.8571280313	1.7298883194
C41	-2.2090862697	7.9058085140	7.2379543467
H42	-2.8646770330	7.7259761925	8.1089115003
H43	-1.2582716492	8.3064504059	7.6224327187
H44	-2.6919602574	8.6739372955	6.6156779734
C45	-1.5920631565	-3.6427727115	1.4895042809
H46	-1.3190244960	-4.4300360008	2.2150919726
H47	-2.5914049241	-3.8889813798	1.1001462152
H48	-0.8678962876	-3.6942964305	0.6619589156
S49	-1.8400024004	0.4768803564	6.9449015979
O50	-2.2167857029	1.8474258231	6.2635925677
O51	-3.3524398125	2.1806826161	3.9914047558
H52	-4.0578952186	2.6452095891	4.7878033740
O53	-2.4181994691	-0.7096404788	6.2902858238
O54	-1.9656699996	0.6432927504	8.4084457330
S55	-1.0767944606	3.7842368794	1.5149614010
O56	-0.7147383280	3.5908313997	0.1003684054
O57	-1.8642152540	4.9900660737	1.8875211841
O58	-3.8871815502	3.4745363956	7.9086517088
H59	-4.3596436479	3.0226066352	8.6314997350
H60	-3.1305909608	2.8824189314	7.7096095516
O61	-5.0217575346	3.0446030477	5.5422853680
H62	-5.4166432638	3.8826712426	5.1242348636
H63	-4.6744148602	3.2696603220	6.4603686723
O64	-4.5718825608	3.9592596128	2.1224245380
H65	-3.7998482293	4.4630341931	1.7844897787
H66	-4.1647444395	3.1387190488	2.4858078479

O67	-5.8052735176	5.1424010922	4.1762289506
H68	-5.4489688796	4.7742648300	3.3110862916
H69	-6.7644243669	5.2421160869	4.0451705347

[Ru^{IV}(bds)(pic)$_2$(OH)]$^+$ T_1
E (PBE/LACVP**++ 2f(Ru))(a.u.) = -2789.930662
ZPE (kcal mol^{-1}) = 317.286
G_{solv} (kcal mol^{-1}) = -59.14
ΔH_{298} (kcal mol^{-1}) = 28.091
ΔS_{298} (cal K^{-1} mol^{-1}) = 258.712
Cartesian coordinates

Atom	x	y	z
C1	2.9161014735	4.2242406127	2.7328622786
C2	1.7287099699	4.4495273816	2.0263824121
C3	0.5855250134	3.7707683155	2.4651935134
N4	0.5680067403	2.9399785367	3.5098419081
C5	1.7207397251	2.6939864030	4.1766937287
C6	2.9201871074	3.3292624434	3.8117272941
H7	3.8394046063	4.7306222072	2.4374188278
H8	1.6838091397	5.1052335977	1.1536623423
H9	3.8446208767	3.1370829993	4.3609333872
Ru10	-1.8043341199	2.1783523386	4.2278192141
C11	1.0485968643	0.0496315886	7.4455471466
C12	2.3776077170	0.3230998790	7.0981289542
C13	2.6522827986	1.1783265016	6.0215633400
C14	1.5816084196	1.7296603425	5.2960116073
N15	0.2988714498	1.4264124244	5.6067697937
C16	0.0536091077	0.6376014147	6.6545799930
H17	0.7832355526	-0.5747920125	8.3016836116
H18	3.1990574306	-0.1134231489	7.6733623685
H19	3.6861581960	1.4099033673	5.7546211010
O20	-1.6845122630	2.5758023028	2.1446090372
C21	-1.7717999820	6.6525244719	6.4396009785
C22	-0.8862580657	5.6192602663	6.8037383915
C23	-0.9205573779	4.3979472020	6.1384655997
N24	-1.7693647114	4.1529996482	5.1063334836
C25	-2.6239711003	5.1375238274	4.7403408762
C26	-2.6544478079	6.3754398230	5.3816991630
H27	-0.1721394871	5.7576848048	7.6204560148
H28	-0.2643147677	3.5766897590	6.4265367268
H29	-3.2844651670	4.9138357324	3.9055475574
H30	-3.3787587521	7.1213891752	5.0435311555
C31	-1.5234642246	-2.3129974766	2.1984552539
C32	-2.6390329330	-1.9316198965	2.9666357360
C33	-2.6861194994	-0.6763674850	3.5701059582
N34	-1.6787772165	0.2181167644	3.4325494435
C35	-0.6032167061	-0.1198029048	2.6791747595
C36	-0.4912467901	-1.3643790750	2.0670820680
H37	-3.4833625737	-2.6130870885	3.1013597012
H38	-3.5343529837	-0.3558586970	4.1750473416
H39	0.1625096985	0.6481462603	2.5616428371
H40	0.3978011359	-1.5842579926	1.4691903705
C41	-1.7845784531	7.9671271242	7.1701271792
H42	-2.3869655368	7.8839207217	8.0924560958
H43	-0.7698817476	8.2695707821	7.4734360208
H44	-2.2238084735	8.7690797289	6.5586880171
C45	-1.4262373798	-3.6708080061	1.5585925147
H46	-0.9448074569	-4.3848528010	2.2507982597
H47	-2.4195475992	-4.0743170221	1.3107765866
H48	-0.8176634565	-3.6421984035	0.6417411060
S49	-1.7166986386	0.4272538701	7.0113034779
O50	-2.2725940949	1.6997264666	6.2528731272
O51	-3.5800642660	2.2078868032	3.9602465723
H52	-4.7376915740	2.4469853562	5.0114946312

O53	-2.1557335433	-0.8444301325	6.4023376416
O54	-1.9102419162	0.6490880703	8.4607617921
S55	-0.9928532034	3.8492264198	1.5576358827
O56	-0.6641141168	3.6486987884	0.1352517942
O57	-1.7103159406	5.0991731620	1.9461615095
O58	-4.0415892060	3.2420751571	7.6651812441
H59	-4.3530014370	2.7873709143	8.4697059515
H60	-3.2678052983	2.7019106190	7.3555668308
O61	-5.4516152620	2.8991496761	5.5843163601
H62	-5.6908205009	3.7952037059	5.0232195554
H63	-4.9678640780	3.1023277253	6.4933824757
O64	-4.4674141056	4.1265553099	2.0893113950
H65	-3.6848075788	4.6545446739	1.8100087916
H66	-4.0664692588	3.2887726328	2.4254083560
O67	-5.8707953883	4.8868291666	4.1447370766
H68	-5.3740112201	4.6642480922	3.2769875375
H69	-6.8030245973	5.0313090743	3.9028221087

$[Ru^{IV}(bds)(pic)_2(OH)]^+$ S_0

E (M06-L/LACVP**++ 2f(Ru))(a.u.) = -2792.259634

ZPE (kcal mol^{-1}) = 327.581

G_{solv} (kcal mol^{-1}) = -63.501

ΔH_{298} (kcal mol^{-1}) = 27.579

ΔS_{298} (cal K^{-1} mol^{-1}) = 251.931

Cartesian coordinates

Atom	x	y	z
C1	2.8031774287	4.1108102792	2.6824354638
C2	1.6234076762	4.3433913346	1.9847911933
C3	0.4604648029	3.7423869494	2.4432398422
N4	0.4176569772	2.9684418197	3.5307980115
C5	1.5762024517	2.6796185207	4.1725245058
C6	2.7832971359	3.2479935171	3.7709533667
H7	3.7341224793	4.5665488384	2.3613569182
H8	1.5883857989	4.9462201695	1.0834949091
H9	3.6975797812	3.0070451666	4.3025587105
Ru10	-1.5585329601	2.1759570869	4.2214348758
C11	0.8011157261	0.0820611706	7.3746525840
C12	2.1345924529	0.3733673107	7.1067206472
C13	2.4398693414	1.2292781538	6.0564942972
C14	1.4122556889	1.7348992660	5.2616183856
N15	0.1208943862	1.3883831758	5.4837376006
C16	-0.1663822899	0.6234470163	6.5426030572
H17	0.4955897071	-0.5194499134	8.2241901195
H18	2.9245196669	-0.0309114162	7.7313462011
H19	3.4667649760	1.5171813013	5.8588272548
O20	-1.6438930765	2.4598556187	2.0920885521
C21	-1.9841088118	6.5927581388	6.4162764473
C22	-0.9391150917	5.7072613568	6.7101020478
C23	-0.8339252396	4.5158908223	6.0238325985
N24	-1.6891276377	4.1701565893	5.0412748313
C25	-2.6938536195	5.0084101691	4.7436187501
C26	-2.8677811506	6.2127817310	5.4069788149
H27	-0.2187322040	5.9384495000	7.4892233315
H28	-0.0526563887	3.7992232212	6.2594606709
H29	-3.3564893661	4.7027898070	3.9434557809
H30	-3.7177317367	6.8275224765	5.1283860355
C31	-1.3952732563	-2.2942589332	2.1138210855
C32	-2.5763536063	-1.8119075166	2.6812330625
C33	-2.5992207510	-0.5825087404	3.3196546844
N34	-1.4991595848	0.1792246787	3.4157530115
C35	-0.3548334447	-0.2592005241	2.8622690445
C36	-0.2641405935	-1.4760932568	2.2173038677
H37	-3.4919443513	-2.3922662171	2.6271961321
H38	-3.4978935949	-0.1741676526	3.7654338383

H39	0.5014403880	0.4058409167	2.9336950452
H40	0.6835116968	-1.7809987881	1.7834112435
C41	-2.1533067583	7.8633867104	7.1795218318
H42	-2.6715087870	7.6677607000	8.1248724742
H43	-1.1906598682	8.3141122035	7.4338692667
H44	-2.7462533874	8.5945819815	6.6276984557
C45	-1.3262252156	-3.6231965258	1.4391857915
H46	-0.9640368835	-4.3866808277	2.1366616495
H47	-2.3043184258	-3.9490769759	1.0813715898
H48	-0.6330683154	-3.6094236681	0.5946600351
S49	-1.9270736132	0.5268212995	6.8755261094
O50	-2.2734612922	1.8652246669	6.1949992276
O51	-3.4105603035	2.1170646306	3.9221826922
H52	-3.9422042015	2.5821992622	4.6749380562
O53	-2.4543385334	-0.6511907503	6.2088905886
O54	-2.0989824881	0.6906666780	8.3154136716
S55	-1.0773735137	3.7686060142	1.5194476130
O56	-0.7172704564	3.6226505607	0.1206942431
O57	-1.8347661559	4.9489458657	1.9388452555
O58	-3.5063665228	3.6239659908	8.1167541645
H59	-4.0990517358	3.6280565320	8.8740670762
H60	-3.0345608498	2.7833461840	8.1897984989
O61	-4.8914289656	3.1856489630	5.7246142269
H62	-5.2887237564	4.0168697271	5.3897595148
H63	-4.4304728466	3.3966341586	6.5616160125
O64	-4.6246235576	4.0583902732	2.1677315412
H65	-3.8617831281	4.5248843836	1.7994123319
H66	-4.2658458653	3.1979246624	2.4323294562
O67	-5.7162333747	5.3957533355	4.3230931585
H68	-5.4758400112	4.9780369518	3.4696933921
H69	-6.6511483999	5.6031468070	4.2510591700

[Ru^{IV}(bds)(pic)$_2$(OH)]$^+$ T_1

E (M06-L/LACVP**++ 2f(Ru))(a.u.) = -2792.262579

ZPE (kcal mol^{-1}) = 327.372

G_{solv} (kcal mol^{-1}) = -59.832

ΔH_{298} (kcal mol^{-1}) = 27.437

ΔS_{298} (cal K^{-1} mol^{-1}) = 257.927

Cartesian coordinates

Atom	x	y	z
C1	2.9790456731	4.0183848627	2.7391508284
C2	1.8245837583	4.3038917314	2.0205948764
C3	0.6424415069	3.7083827966	2.4473027082
N4	0.5590288450	2.8955357478	3.4947946666
C5	1.6798989478	2.6000231586	4.1799093839
C6	2.9111688403	3.1511372020	3.8248026973
H7	3.9290679762	4.4573303034	2.4510229368
H8	1.8275723198	4.9450919195	1.1457938325
H9	3.8058238188	2.9129576762	4.3898004221
Ru10	-1.8310018092	2.2132973407	4.0931706665
C11	0.8433102721	0.1207686093	7.4958536653
C12	2.1792122180	0.3435581371	7.1835216571
C13	2.5057050471	1.1411529512	6.0904802827
C14	1.4794709082	1.6844525832	5.3164864905
N15	0.1892615321	1.4336343971	5.5983525423
C16	-0.1067482793	0.6992325600	6.6609315529
H17	0.5382077994	-0.4629043906	8.3577502329
H18	2.9646458608	-0.0863955739	7.7971407990
H19	3.5451163485	1.3365957419	5.8499817317
O20	-1.6592081655	2.6730784540	2.0324024119
C21	-1.8671295779	6.5966801047	6.4497049382
C22	-1.0031328182	5.5576881872	6.8129884599
C23	-1.0106610618	4.3703161153	6.1082178368
N24	-1.8074562848	4.1684461748	5.0395137011

C25	-2.6422771847	5.1561568282	4.6785179168
C26	-2.7042212003	6.3607362532	5.3585949406
H27	-0.3299376958	5.6674047908	7.6575904578
H28	-0.3737881111	3.5411193072	6.3928575230
H29	-3.2605277330	4.9579572889	3.8134844622
H30	-3.4108850017	7.1137007627	5.0232910474
C31	-1.4151317462	-2.3127575826	2.2145224232
C32	-2.4777910646	-1.9768373797	3.0571964940
C33	-2.5731660125	-0.7013321815	3.5864068388
N34	-1.6630107938	0.2476675839	3.3085721211
C35	-0.6413161741	-0.0478466364	2.4821079685
C36	-0.4856463238	-1.3051428532	1.9329084719
H37	-3.2388484290	-2.7089198104	3.3077498441
H38	-3.3785509825	-0.4111402200	4.2506416239
H39	0.0385504608	0.7666617622	2.2568715414
H40	0.3535439415	-1.4947862198	1.2705392368
C41	-1.9040144358	7.8781365325	7.2132551127
H42	-2.5372663417	7.7751416261	8.1010171073
H43	-0.9105827396	8.1658970292	7.5656812162
H44	-2.3114566944	8.6960547140	6.6163936488
C45	-1.2613244076	-3.6842607452	1.6476967560
H46	-0.5962948092	-4.2839085499	2.2787395263
H47	-2.2151846915	-4.2116227239	1.5912065231
H48	-0.8162751039	-3.6595867599	0.6502970096
S49	-1.8702382475	0.5413042140	6.9634497180
O50	-2.4194381301	1.6708187050	6.0890047556
O51	-3.5975444689	2.2482305782	3.8332535270
H52	-4.4489007421	2.3758363104	5.2390528132
O53	-2.2589384863	-0.7970570520	6.5379003323
O54	-2.1016550298	0.9250780264	8.3650814893
S55	-0.8994733936	3.9182103728	1.5390454759
O56	-0.5646293569	3.7901121455	0.1307807006
O57	-1.5332658600	5.1579846006	2.0029679910
O58	-3.8018306484	3.2306277039	8.0862840428
H59	-4.3627130107	3.2296943622	8.8680748000
H60	-3.1749604306	2.4926123041	8.2230694578
O61	-5.0644822714	2.8313810602	5.8858519604
H62	-5.3746679586	3.6705276986	5.3780607057
H63	-4.5549520817	3.0221858929	6.7563189963
O64	-4.4387681050	4.2922128204	2.0554520457
H65	-3.7075049280	4.8410363719	1.7381627789
H66	-4.0234008748	3.4321406280	2.2308153750
O67	-5.7269598526	4.7940480262	4.3938566783
H68	-5.3212188260	4.6561461409	3.5007905276
H69	-6.6653902717	4.9317433334	4.2371734592

$[Ru^{V}=O]^{+}$

E (B3LYP-D3/LACVP**++ 2f(Ru))(a.u.) = -2791.835435

ZPE (kcal mol^{-1}) = 319.645

G_{solv} (kcal mol^{-1}) = -67.348

ΔH_{298} (kcal mol^{-1}) = 28.599

ΔS_{298} (cal K^{-1} mol^{-1}) = 265.147

Cartesian coordinates

Atom	x	y	z
C1	2.3535031568	3.3210346087	1.7930726939
C2	1.1951594857	4.0461138725	1.5373540183
C3	0.0887456095	3.7933606894	2.3373352977
N4	0.0807483734	2.9160113225	3.3409946422
C5	1.2176651248	2.2371806320	3.6227217063
C6	2.3666966216	2.4114843679	2.8505738677
H7	3.2386142039	3.4594858240	1.1812120115
H8	1.1182810927	4.7668516849	0.7318520811
H9	3.2663967860	1.8519342767	3.0754042967
Ru10	-1.9860919053	2.3322158777	4.3847747993

C11	0.8412853001	0.1273810035	7.2611588634
C12	2.0422956774	-0.0025997829	6.5668722775
C13	2.1981513735	0.6544591669	5.3479287772
C14	1.1409037873	1.4082800660	4.8306145221
N15	-0.0403316722	1.4842997883	5.4822127060
C16	-0.1597070937	0.8821618184	6.6653803346
H17	0.6590508034	-0.3439618776	8.2201948244
H18	2.8533947260	-0.5976713844	6.9736569753
H19	3.1346386706	0.5848499519	4.8083522950
O20	-2.3554852092	3.3793247122	2.5387622210
C21	-1.8721664746	6.3440189523	7.2341176209
C22	-0.7118943468	5.8633522423	6.6091689910
C23	-0.7765958380	4.7501547669	5.7949759417
N24	-1.9322581147	4.0876778038	5.5767899612
C25	-3.0593843427	4.5146667143	6.1804415068
C26	-3.0532848107	5.6368534669	6.9987743539
H27	0.2422595762	6.3613533500	6.7497115217
H28	0.1063191705	4.3735322876	5.2972529460
H29	-3.9603566301	3.9170239442	6.0511322161
H30	-3.9869932904	5.9441688834	7.4580778932
C31	-1.8166595993	-1.8867863829	1.8137403137
C32	-2.3466221311	-1.8353686217	3.1141497350
C33	-2.3679427095	-0.6423681786	3.8148092407
N34	-1.8813088728	0.5010042629	3.2787368818
C35	-1.3745491231	0.4764574851	2.0316999811
C36	-1.3237066915	-0.6914868463	1.2847442165
H37	-2.7562581959	-2.7255521620	3.5812662375
H38	-2.7769204281	-0.5707923217	4.8149887203
H39	-1.0299545508	1.4051745814	1.5985876091
H40	-0.9176013833	-0.6377546972	0.2803753960
C41	-1.8225604317	7.5500970931	8.1306834016
H42	-1.3139537807	7.3018862825	9.0698580380
H43	-1.2588566597	8.3629624153	7.6619553923
H44	-2.8221451429	7.9157619467	8.3741997478
C45	-1.7915456063	-3.1755225108	1.0396286599
H46	-1.0804493574	-3.8780131769	1.4898930826
H47	-2.7742306191	-3.6576076273	1.0571067064
H48	-1.5014082913	-3.0170070131	-0.0010078223
S49	-1.7529296854	1.1869983745	7.4332038080
O50	-2.5764050322	1.2140663831	6.1241354790
O51	-3.6931230310	2.3449379737	4.2751649292
O52	-2.1245210221	0.0316439564	8.2477526809
O53	-1.6340079901	2.4969725810	8.0865601020
S54	-1.4990260931	4.6097828927	2.1182653200
O55	-1.6823807763	4.8920315262	0.6950307982
O56	-1.5482577424	5.7045635805	3.0820698440
O57	-4.3189826878	2.1822475484	9.1728534748
H58	-3.4499614998	2.6100056177	9.1468708570
H59	-4.1180584985	1.2702024633	9.4238106986
O60	-5.2986256856	2.4748071605	6.5809718896
H61	-5.0435357829	2.3645876234	7.5219520975
H62	-4.9679601478	1.6749569246	6.1552684026
O63	-3.4174208478	1.8740589176	0.3596806745
H64	-3.3386047036	2.4998741409	1.0989811388
H65	-4.1313954406	2.2151435726	-0.1934344042
O66	-0.7626909493	2.3035618526	-0.3132328526
H67	-0.7900265186	3.2524984320	-0.4948546484
H68	-1.7164737768	2.0765014686	-0.2917258209

Transition state of WNA

E (B3LYP-D3/LACVP**++ 2f(Ru))(a.u.) = -2791.813852

ZPE (kcal mol^{-1}) = 317.839

G_{solv} (kcal mol^{-1}) = -66.309

ΔH_{298} (kcal mol^{-1}) = 27.287

ΔS_{298} (cal K^{-1} mol^{-1}) = 251.703

Cartesian coordinates

Atom	x	y	z
C1	2.2066424499	4.0804768659	2.0912831814
C2	0.9399173854	4.5716966343	1.8027341672
C3	-0.1338176465	4.0262117093	2.5002109820
N4	-0.0278736692	3.0922677493	3.4408279384
C5	1.2038700092	2.6008710848	3.7102728375
C6	2.3382557597	3.0767876438	3.0502344590
H7	3.0805212335	4.4621070485	1.5732428859
H8	0.7664661461	5.3309691849	1.0485442135
H9	3.3173560968	2.6789177580	3.2885532360
Ru10	-2.1837889616	2.2948093776	4.4193055744
C11	1.1118112946	-0.2280394939	6.8666449078
C12	2.2983718834	-0.1167068550	6.1382100342
C13	2.3803983195	0.7920779735	5.0831125340
C14	1.2557815759	1.5642900793	4.7627643464
N15	0.1096708683	1.4226883771	5.4400869683
C16	0.0562659849	0.5787992247	6.4594588739
H17	0.9936807804	-0.9123822735	7.6989089092
H18	3.1560719854	-0.7316200195	6.3922167921
H19	3.2994179611	0.8836126095	4.5162438304
O20	-2.6026087806	3.3231516734	2.6483070440
C21	-1.9457118559	6.4480693382	7.0997100711
C22	-0.9955002786	5.4270233286	7.2350395537
C23	-1.1072427867	4.2682772893	6.4862387995
N24	-2.1105306434	4.0728239669	5.6028241940
C25	-3.0227811637	5.0536082912	5.4505618748
C26	-2.9781429936	6.2316649152	6.1801515386
H27	-0.1644348190	5.5297858107	7.9257814480
H28	-0.3923683925	3.4656994444	6.5886786954
H29	-3.7658429150	4.8996621408	4.6779707998
H30	-3.7326861140	6.9911226781	6.0038463828
C31	-1.8358311293	-1.8932583187	1.8517136367
C32	-2.6851988692	-1.7798121394	2.9630045270
C33	-2.7857243359	-0.5803301633	3.6482793871
N34	-2.0753469813	0.5083692843	3.2794367090
C35	-1.2626906715	0.4234830468	2.2090405790
C36	-1.1217563035	-0.7497175869	1.4828476879
H37	-3.2784469791	-2.6260651839	3.2947858777
H38	-3.4309463004	-0.4633446090	4.5089063857
H39	-0.7424413197	1.3181827095	1.8976912654
H40	-0.4681877484	-0.7440757203	0.6168229673
C41	-1.8518159246	7.7084986562	7.9143451407
H42	-2.2255319033	7.5301400266	8.9298837015
H43	-0.8135424664	8.0413749376	8.0054424925
H44	-2.4403243634	8.5182978618	7.4772620462
C45	-1.7005531944	-3.1913617522	1.1041046784
H46	-1.0621890624	-3.8859765263	1.6635127881
H47	-2.6729370201	-3.6763410694	0.9769889658
H48	-1.2531975189	-3.0434592228	0.1185451880
S49	-1.5403644262	0.6224668245	7.2890169240
O50	-2.4705511497	0.9665287273	6.1262186759
O51	-4.0085022493	2.0816662331	4.4406525052
O52	-1.7996412329	-0.6899446966	7.8696137417
O53	-1.4844220331	1.7883432156	8.2278889697
S54	-1.8151453326	4.5753810388	2.1498100733
O55	-1.9568832078	4.6874929611	0.6962959040
O56	-2.0631416768	5.7631880666	2.9696223569
O57	-3.7812419852	3.0054739121	7.9053591086
H58	-2.8674730694	2.6068724005	8.0571674268
H59	-4.3450223762	2.6609392379	8.6131718674
O60	-4.8476103108	2.5118831283	5.7843265817
H61	-4.2451579718	2.6976446739	6.7438605534

H62	-5.2439549097	3.3408383646	5.4691205297
O63	-3.1044713137	1.8181872391	0.2386500999
H64	-3.1276076412	2.3109067348	1.0770070437
H65	-3.5285872533	2.4142444462	-0.3929388155
O66	-0.3675901144	2.2720329365	-0.0077583371
H67	-0.3787451662	3.1912673350	-0.3022156523
H68	-1.3156605249	2.0259614421	-0.0712491784

Product of WNA

E (B3LYP-D3/LACVP**++ 2f(Ru))(a.u.) = -2791.448376

ZPE (kcal mol^{-1}) = 313.713

G_{solv} (kcal mol^{-1}) = -38.386

ΔH_{298} (kcal mol^{-1}) = 27.486

ΔS_{298} (cal K^{-1} mol^{-1}) = 254.333

Cartesian coordinates

Atom	x	y	z
C1	2.4092114533	3.2347845589	1.7383034297
C2	1.2383130589	3.9635764416	1.5306461333
C3	0.2165709886	3.7829154172	2.4578939935
N4	0.2901399512	2.9693230311	3.5000004075
C5	1.4165455808	2.2734669885	3.7122538306
C6	2.5075133188	2.3850792029	2.8402698562
H7	3.2392604027	3.3218243532	1.0438123348
H8	1.0972588164	4.6253053759	0.6843228900
H9	3.4129655522	1.8123594642	3.0050362115
Ru10	-2.1280106976	2.2486015135	4.5962445910
C11	1.1095844565	-0.0838482191	7.2307379220
C12	2.3280501191	-0.0900969758	6.5541008069
C13	2.4732745593	0.6666455250	5.3910447626
C14	1.3854383079	1.4133399480	4.9227510620
N15	0.2124230783	1.4108223409	5.5787699475
C16	0.0908060372	0.6944922689	6.6882907320
H17	0.9291190892	-0.6601868184	8.1305987405
H18	3.1607120913	-0.6795299024	6.9260412689
H19	3.4190903873	0.6726151639	4.8622398015
O20	-2.3150865059	3.5269529601	2.8179925169
C21	-1.9511976309	6.5737683203	6.9319513587
C22	-0.8454048133	5.7147597420	6.8905461551
C23	-0.9616176101	4.4710349574	6.2973193343
N24	-2.1129269985	4.0387513612	5.7451529624
C25	-3.1900757015	4.8404996569	5.7983225249
C26	-3.1406255507	6.1031871316	6.3748528710
H27	0.1090176296	6.0165691747	7.3111257898
H28	-0.1229619918	3.7902153918	6.2439631346
H29	-4.0979885196	4.4522570465	5.3579480080
H30	-4.0368484686	6.7148864036	6.3798347533
C31	-1.7878951831	-1.7314450936	1.6220938550
C32	-2.2114064218	-1.8528942432	2.9533990108
C33	-2.3041782263	-0.7319677646	3.7649496342
N34	-1.9758098310	0.4997925793	3.3213109860
C35	-1.5680312382	0.6302083291	2.0464441325
C36	-1.4726565885	-0.4464735376	1.1748940234
H37	-2.4877400536	-2.8213454055	3.3600126604
H38	-2.6525169296	-0.7889985157	4.7883840170
H39	-1.3573505062	1.6250381230	1.6855473306
H40	-1.1750138122	-0.2468278494	0.1506520188
C41	-1.8498155279	7.9355360071	7.5671082179
H42	-1.8296126538	7.8487173688	8.6600945833
H43	-0.9279215657	8.4422839078	7.2632994079
H44	-2.6971226429	8.5704726497	7.2962370243
C45	-1.6740693565	-2.9368459228	0.7259767624
H46	-0.7509484486	-3.4890496833	0.9418444435
H47	-2.5084982670	-3.6276354046	0.8824569755
H48	-1.6526339759	-2.6511492250	-0.3288741316

S49	-1.5432271915	0.8241003070	7.4565958077
O50	-2.4223867345	0.8916186573	6.1899960349
O51	-4.0266062170	2.3363273205	4.5517966135
O52	-1.7693385181	-0.4226756954	8.1892618621
O53	-1.5447503455	2.0963776324	8.2004446389
S54	-1.3645894937	4.6506039351	2.3462765512
O55	-1.5980195304	4.9302265534	0.9156732947
O56	-1.3089352692	5.7836638749	3.2705285751
O57	-4.4391182726	2.8756638548	7.5535280369
H58	-3.5518544745	2.7677239586	7.9251318236
H59	-4.4063920930	2.3350122486	6.7519845255
O60	-4.6858122067	1.2341330607	3.9197700853
H62	-4.6293721921	1.4981998777	2.9683124889
O63	-4.2134368223	2.2988758432	1.3896105676
H64	-3.5703231195	2.8982893617	1.8339591989
H65	-4.9426410916	2.8680992978	1.1113144573
O66	-1.8610647017	2.3609744210	-0.4427675410
H67	-1.7253620352	3.2981473767	-0.2279240953
H68	-2.7470932533	2.1834477147	-0.0877333591

Prereactive complex of I2M

E (B3LYP-D3/LACVP**++ 2f(Ru))(a.u.) = -4972.067604

ZPE (kcal mol^{-1}) = 515.012

G_{solv} (kcal mol^{-1}) = -136.471

ΔH_{298} (kcal mol^{-1}) = 42.628

ΔS_{298} (cal K^{-1} mol^{-1}) = 360.344

Cartesian coordinates

Atom	x	y	z
C1	1.8776221204	1.8316944346	1.8925754954
C2	0.7161038848	2.1703709199	1.2018657612
C3	-0.4233820148	2.4393442108	1.9483815280
N4	-0.4716984102	2.3857509432	3.2839438817
C5	0.6731976824	2.1272615108	3.9605207240
C6	1.8606581921	1.8302507867	3.2863959245
H7	2.7896604703	1.5898331227	1.3563150102
H8	0.6664753064	2.2185091151	0.1195632429
H9	2.7613082308	1.6003267477	3.8421294725
Ru10	-2.5583177270	2.3708233731	4.3877138271
C11	0.1368657900	2.6493411527	8.1140791655
C12	1.4326638203	2.4184848581	7.6597192836
C13	1.6487176295	2.2169632902	6.2968762796
C14	0.5638167329	2.2544151835	5.4161762585
N15	-0.6929354243	2.4743488824	5.8636562602
C16	-0.8833168518	2.6597803276	7.1705154048
H17	-0.0999023100	2.7946570625	9.1623075782
H18	2.2653578684	2.3915604426	8.3553349887
H19	2.6509114331	2.0415908943	5.9272672012
O20	-2.9028280371	2.3408631582	2.2528662835
C21	-3.1331829844	7.2289582352	4.7928439142
C22	-1.9481363671	6.6980666185	4.2637754912
C23	-1.7911726884	5.3275810499	4.1428731903
N24	-2.7561673238	4.4696629851	4.5297126169
C25	-3.9042524214	4.9578182477	5.0515357463
C26	-4.1203192500	6.3164649120	5.1870557648
H27	-1.1477533884	7.3505155431	3.9306497327
H28	-0.8981980618	4.8997614868	3.7088411809
H29	-4.6433377477	4.2402498466	5.3762932422
H30	-5.0592494266	6.6427073889	5.6218833557
C31	-2.8500579477	-2.5519491378	4.4129821956
C32	-2.4293788003	-1.8434024669	5.5451824941
C33	-2.2281049365	-0.4742925367	5.4754706231
N34	-2.4023253121	0.2208473105	4.3333034447
C35	-2.7668284451	-0.4484101070	3.2171305052
C36	-2.9882025783	-1.8150619543	3.2264647815

H37	-2.2914757636	-2.3489419858	6.4955457405
H38	-1.9700118254	0.0960058540	6.3568158059
H39	-2.9056006973	0.1514668540	2.3264377125
H40	-3.2949167069	-2.3050529005	2.3080576083
C41	-3.3289235931	8.7097761571	4.9572742634
H42	-3.1323263487	8.9962708127	5.9973041180
H43	-2.6531926983	9.2825330104	4.3179585758
H44	-4.3585553328	9.0003582106	4.7303286238
C45	-3.1839445411	-4.0138778504	4.4644623568
H46	-2.8199654300	-4.4831270542	5.3810982625
H47	-4.2734811095	-4.1374263584	4.4255610132
H48	-2.7656157545	-4.5470671411	3.6051502097
S49	-2.6133960966	2.8632171866	7.6269633805
O50	-3.1985834750	2.1242462841	6.3985124369
O51	-4.2528110515	2.2618352472	4.1887944127
O52	-2.8395340283	2.0488798569	8.8231392090
O53	-2.9125470948	4.2910007265	7.6550920595
S54	-1.9655472951	2.9982866767	1.2144942023
O55	-2.1274240706	2.3479788163	-0.0850359938
O56	-1.9528180125	4.4579728968	1.3049605340
C57	-10.9151485150	-2.1102014510	4.3528218364
C58	-9.7072913274	-2.7879876922	4.5042650810
C59	-8.6219126182	-2.0705208353	4.9884798397
N60	-8.6687793546	-0.7724428297	5.3102458953
C61	-9.8569925166	-0.1280560951	5.2300675576
C62	-10.9951407735	-0.7725899449	4.7375464272
H63	-11.7891179966	-2.6187393178	3.9583869731
H64	-9.5823880587	-3.8350356839	4.2505851974
H65	-11.9342193750	-0.2399685625	4.6572853828
Ru66	-6.6841823586	0.4172819831	5.7105191003
C67	-9.6187055088	3.7263785668	6.9374238315
C68	-10.8720531599	3.2849553207	6.5239826148
C69	-10.9856003493	2.0277903426	5.9323646061
C70	-9.8467498140	1.2362674802	5.7661397402
N71	-8.6298050620	1.6757909992	6.1607676715
C72	-8.5363214956	2.8813384800	6.7244273557
H73	-9.4589274812	4.6990425649	7.3894249315
H74	-11.7505073862	3.9084566343	6.6564993441
H75	-11.9547507097	1.6670851885	5.6117315736
O76	-6.1823154296	-1.5656514733	4.9921337035
C77	-6.4687937162	-0.4992657726	10.5319945268
C78	-7.5763999487	-0.9190693835	9.7796796578
C79	-7.6140801405	-0.7018313315	8.4135062459
N80	-6.6058538146	-0.0808149007	7.7668181971
C81	-5.5269968452	0.3272156695	8.4695900136
C82	-5.4283258525	0.1250097842	9.8346880833
H83	-8.4104032444	-1.4245652251	10.2556823998
H84	-8.4477852894	-1.0398031664	7.8141538872
H85	-4.7495855899	0.8430335957	7.9257538870
H86	-4.5407882826	0.4890108295	10.3399207555
C87	-6.0995115063	2.1748045387	1.1563510344
C88	-6.7514129646	2.9298630444	2.1395506708
C89	-7.0552982265	2.3650921113	3.3679826595
N90	-6.7616259236	1.0807914225	3.6596302838
C91	-6.1749345845	0.3210665896	2.7089012829
C92	-5.8415916319	0.8310568334	1.4659240069
H93	-6.9929607831	3.9734741202	1.9658592885
H94	-7.4957626717	2.9586153094	4.1558483650
H95	-5.9464876009	-0.6994762610	2.9909455770
H96	-5.3473636340	0.1871538542	0.7452554309
C97	-6.4202608383	-0.6820392765	12.0233243674
H98	-6.8374897590	0.2039405029	12.5183194063
H99	-7.0075620434	-1.5466494542	12.3433700105
H100	-5.3936291214	-0.8018720777	12.3787693853

C101	-5.6751402530	2.7615691922	-0.1582322255
H102	-6.2248482907	2.2932786830	-0.9829993040
H103	-5.8499184747	3.8387995430	-0.1980371650
H104	-4.6096555962	2.5738850978	-0.3295861800
S105	-6.8529043431	3.3632975753	7.1469435592
O106	-6.1470535320	2.4375564096	6.1283737983
O107	-4.9777072407	0.3499566941	5.6520145451
O108	-6.6866094382	4.7551622292	6.7220420022
O109	-6.6175162239	2.9961498579	8.5395142033
S110	-7.0246045854	-2.8144038479	5.3416227863
O111	-6.7742488645	-3.8797642648	4.3725609947
O112	-7.0259355954	-3.1156444186	6.7728141266

Transition state of I2M

E (B3LYP-D3/LACVP**++ 2f(Ru))(a.u.) = -4972.057168

ZPE (kcal mol^{-1}) = 514.314

G_{solv} (kcal mol^{-1}) = -135.827

ΔH_{298} (kcal mol^{-1}) = 42.281

ΔS_{298} (cal K^{-1} mol^{-1}) = 357.9

Cartesian coordinates

Atom	x	y	z
C1	2.2673993451	1.6714304353	1.8555686209
C2	1.0912770366	1.9254082170	1.1534129232
C3	-0.0440310137	2.2431024113	1.8866248292
N4	-0.0749478389	2.3043689640	3.2236494318
C5	1.0848338056	2.1370944549	3.9056716427
C6	2.2688351647	1.8029391425	3.2431447234
H7	3.1770481077	1.3966829296	1.3310977549
H8	1.0294845423	1.8756383305	0.0716433769
H9	3.1826602231	1.6480173832	3.8026117606
Ru10	-2.0869691174	2.4127120608	4.3532604113
C11	0.5887309423	3.0155190771	8.0059187042
C12	1.8806970669	2.7537486240	7.5588603662
C13	2.0811641835	2.4364231885	6.2164091469
C14	0.9906959015	2.4008414867	5.3436484247
N15	-0.2610462813	2.6638070364	5.7836353495
C16	-0.4406611760	2.9470195804	7.0755269430
H17	0.3607265830	3.2397102296	9.0423024513
H18	2.7210651499	2.7852884739	8.2450762715
H19	3.0792091341	2.2233728256	5.8547675371
O20	-2.5179751601	2.2512611866	2.2577260270
C21	-2.6503388377	7.2993913392	4.3442246541
C22	-1.4831425002	6.7158950000	3.8283104391
C23	-1.3348241755	5.3391535317	3.8263373614
N24	-2.2893281332	4.5224410398	4.3170849562
C25	-3.4216849054	5.0628610432	4.8188518587
C26	-3.6297503813	6.4307748028	4.8404317102
H27	-0.6905907648	7.3319055232	3.4158259327
H28	-0.4551386345	4.8696790535	3.4070034927
H29	-4.1563579333	4.3792486838	5.2174635860
H30	-4.5560000034	6.7994069827	5.2692490805
C31	-2.6183361431	-2.4647172598	4.7229827367
C32	-2.1572724299	-1.7006076882	5.8027465124
C33	-1.8662193797	-0.3568241669	5.6320272093
N34	-1.9958770302	0.2612805660	4.4404375933
C35	-2.4008859723	-0.4678091942	3.3759991975
C36	-2.7102689168	-1.8129216494	3.4847981496
H37	-2.0568171277	-2.1405568737	6.7898388227
H38	-1.5724818276	0.2605316964	6.4698184927
H39	-2.5144425706	0.0696865479	2.4435363580
H40	-3.0663985197	-2.3464235208	2.6094544947
C41	-2.8276012508	8.7913759431	4.3886184580
H42	-2.4796920824	9.1768467099	5.3546708096
H43	-2.2509115710	9.2904744293	3.6056622121

H44	-3.8792180127	9.0718638755	4.2857592189
C45	-3.0076393430	-3.9071467084	4.8721068951
H46	-3.1450059957	-4.1813988958	5.9204142342
H47	-3.9372367608	-4.1086447375	4.3318142630
H48	-2.2312675115	-4.5560262835	4.4489187039
S49	-2.1690998197	3.1418239242	7.5427393690
O50	-2.7383304309	2.3088801783	6.3671569744
O51	-3.8293312847	2.3312412315	4.1974541756
O52	-2.3727026406	2.3944579495	8.7842993487
O53	-2.5038136729	4.5601142032	7.4805699430
S54	-1.5962451064	2.7314536673	1.1177251266
O55	-1.8191053775	1.9195079764	-0.0766871733
O56	-1.5478277010	4.1904904106	1.0017919906
C57	-10.4208679273	-1.5480390400	3.5572244346
C58	-9.2045584404	-2.2158485993	3.6843629145
C59	-8.1395609103	-1.5197548575	4.2390601886
N60	-8.2142331035	-0.2466658579	4.6465933971
C61	-9.4145145970	0.3808964228	4.5976156853
C62	-10.5324908671	-0.2449905532	4.0399062726
H63	-11.2781745348	-2.0413068259	3.1103543082
H64	-9.0590104375	-3.2412759192	3.3620684339
H65	-11.4809802994	0.2751953716	3.9889611299
Ru66	-6.3145355010	0.9258866105	5.2070478402
C67	-9.2738150583	4.0882896705	6.6126043219
C68	-10.5063514535	3.6772969819	6.1132621232
C69	-10.5870154231	2.4717071210	5.4183747703
C70	-9.4388569385	1.6957171779	5.2424915747
N71	-8.2435548292	2.1037229917	5.7279929695
C72	-8.1787752742	3.2656768330	6.3817785516
H73	-9.1371788124	5.0250565369	7.1418752985
H74	-11.3927034573	4.2874636932	6.2549999592
H75	-11.5378437105	2.1394016794	5.0211403647
O76	-5.7122845585	-0.9888114108	4.4279723069
C77	-6.0285469824	-0.5153275071	9.9037597065
C78	-7.1441549126	-0.8514780765	9.1235952131
C79	-7.2112917967	-0.4568582709	7.7977886095
N80	-6.2253111374	0.2562559032	7.2180582028
C81	-5.1435290035	0.5960706185	7.9533058935
C82	-5.0162405262	0.2234549494	9.2791084356
H83	-7.9599772757	-1.4311990791	9.5431225893
H84	-8.0522465874	-0.7293258926	7.1747050326
H85	-4.3847275649	1.1880083109	7.4635553645
H86	-4.1283008838	0.5411580309	9.8151196826
C87	-5.4790117624	2.9003325981	0.7944667832
C88	-6.1489190117	3.6285305813	1.7903885786
C89	-6.5333889754	3.0115609945	2.9681454031
N90	-6.3036516155	1.6995717047	3.1974157141
C91	-5.7091395985	0.9717514482	2.2289389564
C92	-5.2967675199	1.5334224110	1.0310061559
H93	-6.3383474759	4.6900112853	1.6644556693
H94	-6.9877943456	3.5745502902	3.7715754377
H95	-5.5292000013	-0.0706726090	2.4597220682
H96	-4.7852570533	0.9123862983	0.3032782753
C97	-5.9342510614	-0.9011688081	11.3533563736
H98	-6.2279917895	-0.0523305066	11.9824115888
H99	-6.5917036947	-1.7398293496	11.5945063232
H100	-4.9094036436	-1.1672905495	11.6271575381
C101	-4.9359158064	3.5737148799	-0.4328024731
H102	-5.6588015194	4.2798744538	-0.8526283532
H103	-4.0364858710	4.1425490395	-0.1662334178
H104	-4.6519010542	2.8518282726	-1.2008419776
S105	-6.5093336434	3.7268498441	6.8772895953
O106	-5.7970654173	2.9026937961	5.7762441078
O107	-4.5621250267	0.9001202169	5.2098306440

O108	-6.3397545022	5.1496497235	6.5805671103
O109	-6.2815175663	3.2292428346	8.2292414299
S110	-6.5474179543	-2.2776278318	4.5964036564
O111	-6.2204932444	-3.2314347210	3.5378245694
O112	-6.6195676722	-2.7357032159	5.9835148207

Product of I2M

E (B3LYP-D3/LACVP**++ 2f(Ru))(a.u.) = -4972.088237

ZPE (kcal mol^{-1}) = 515.055

G_{solv} (kcal mol^{-1}) = -135.105

ΔH_{298} (kcal mol^{-1}) = 41.975

ΔS_{298} (cal K^{-1} mol^{-1}) = 358.184

Cartesian coordinates

Atom	x	y	z
C1	2.1604285323	1.1893541794	1.7512411291
C2	0.9720628904	1.5593395367	1.1250850302
C3	-0.0782612016	1.9799881810	1.9284859950
N4	-0.0158918406	2.0326676940	3.2640364784
C5	1.1638153993	1.7595959359	3.8726364574
C6	2.2639365598	1.3184553760	3.1349951623
H7	3.0046103900	0.8312763215	1.1707306914
H8	0.8376930391	1.5244368766	0.0493707263
H9	3.1951623583	1.0829395220	3.6351661602
Ru10	-1.8558401090	2.3472313479	4.5034024796
C11	0.9642520905	2.7507212795	7.9697826648
C12	2.1909996271	2.3086273158	7.4796026855
C13	2.2941223129	1.9462048316	6.1378042264
C14	1.1757782555	2.0454900331	5.3069608508
N15	-0.0106935085	2.4819409915	5.7903035869
C16	-0.1014247397	2.8060523957	7.0823947410
H17	0.8092161338	3.0193374860	9.0091837808
H18	3.0535398985	2.2349512834	8.1341848230
H19	3.2371053423	1.5890930623	5.7430466587
O20	-2.4959671108	2.2367677059	2.4816887019
C21	-2.1564995129	7.2499931464	4.2127659772
C22	-0.9924789046	6.5731821852	3.8208264723
C23	-0.9263229903	5.1938100962	3.9158389934
N24	-1.9544728149	4.4583954510	4.3837720706
C25	-3.0805737285	5.0917279241	4.7777354771
C26	-3.2113729617	6.4680663885	4.6977483194
H27	-0.1397979328	7.1177107137	3.4282321726
H28	-0.0448359863	4.6531889808	3.5953270568
H29	-3.8804376091	4.4763249308	5.1648851732
H30	-4.1396089196	6.9187642073	5.0351578623
C31	-2.5389169125	-2.5433109283	4.8533657688
C32	-2.1900687879	-1.7696494477	5.9692749356
C33	-1.9013743229	-0.4235654105	5.8222351530
N34	-1.9273152558	0.1928797673	4.6216448044
C35	-2.2355115919	-0.5413671867	3.5325265355
C36	-2.5347460389	-1.8926905749	3.6136457782
H37	-2.1687422672	-2.2090408215	6.9617660566
H38	-1.6845132330	0.1993051606	6.6797242755
H39	-2.2824113525	-0.0110953910	2.5905833890
H40	-2.8134549819	-2.4265956988	2.7126990295
C41	-2.2524613660	8.7484899519	4.1417997526
H42	-1.8985387033	9.1884955780	5.0819343135
H43	-1.6353292129	9.1531391791	3.3351763265
H44	-3.2845547773	9.0770541494	3.9946589860
C45	-2.9561614327	-3.9786510069	4.9978046102
H46	-2.3666409343	-4.4906840671	5.7639261990
H47	-4.0079248008	-4.0155455739	5.3058561119
H48	-2.8690661801	-4.5236501811	4.0555483156
S49	-1.7770507182	3.2274877868	7.5945284897
O50	-2.4580653729	2.3505354321	6.4976808975

O51	-3.7581778267	2.3870122746	4.4454138208
O52	-2.0238951227	2.6055384040	8.8928004064
O53	-1.9681318277	4.6603500228	7.4132103188
S54	-1.6190641404	2.6398718144	1.2738748379
O55	-2.0017685587	1.8645404311	0.0939466272
O56	-1.4270185210	4.0836086207	1.1522239967
C57	-9.9131676394	-1.3666776050	2.9118132754
C58	-8.6732152396	-1.9601930268	3.1383706760
C59	-7.7093356089	-1.2168363881	3.8062067365
N60	-7.9022131844	0.0388445706	4.2265514331
C61	-9.1320573968	0.5911933059	4.0813954740
C62	-10.1526661182	-0.0874325797	3.4112289927
H63	-10.6921983040	-1.9005923180	2.3770711964
H64	-8.4346774584	-2.9663934160	2.8106052591
H65	-11.1249069280	0.3736619199	3.2881943992
Ru66	-6.2017239658	1.2939698154	5.0012015150
C67	-9.3622931229	4.2527034490	6.1652895050
C68	-10.5188022004	3.8055069048	5.5306178331
C69	-10.4780843342	2.6127883228	4.8116559396
C70	-9.2892914432	1.8822158973	4.7492599974
N71	-8.1715374430	2.3217851717	5.3728570581
C72	-8.2191182562	3.4752122304	6.0431693400
H73	-9.3174951660	5.1863003034	6.7156688929
H74	-11.4369650601	4.3817543867	5.5832436194
H75	-11.3643129786	2.2563474205	4.3017498420
O76	-5.3607914449	-0.5553987499	4.3523954119
C77	-6.0845566208	-0.3624736762	9.6389400598
C78	-7.1482349343	-0.6865176096	8.7839249778
C79	-7.1642925589	-0.2134817282	7.4830596135
N80	-6.1807126193	0.5680995178	6.9938461977
C81	-5.1492710519	0.8941330833	7.8024698098
C82	-5.0705056791	0.4435007861	9.1096325852
H83	-7.9621816669	-1.3169101896	9.1258457889
H84	-7.9668765803	-0.4726533281	6.8045178609
H85	-4.3832906906	1.5325235863	7.3873310611
H86	-4.2184503080	0.7490545442	9.7090192542
C87	-5.2755599185	3.1613493296	0.5203430657
C88	-5.8506448631	3.9469365671	1.5277576088
C89	-6.2151528113	3.3772120128	2.7382560448
N90	-6.0447222897	2.0629188619	2.9926133858
C91	-5.5176639711	1.2861406692	2.0224908157
C92	-5.1353208356	1.7940788896	0.7930302849
H93	-5.9923017542	5.0133235798	1.3834575980
H94	-6.6082332546	3.9806637356	3.5451880997
H95	-5.3617028506	0.2449959030	2.2725902069
H96	-4.6739215185	1.1332110116	0.0674577217
C97	-6.0555536241	-0.8333977711	11.0664373689
H98	-6.5784564236	-0.1114570130	11.7054099499
H99	-6.5561407796	-1.7984797308	11.1825613862
H100	-5.0319138574	-0.9194647625	11.4398623887
C101	-4.8027216322	3.7405605961	-0.7819911826
H102	-5.4032162769	3.3552062916	-1.6140551090
H103	-4.8656159618	4.8304100458	-0.7884633207
H104	-3.7649366159	3.4442959584	-0.9656019446
S105	-6.6195252673	3.9899895078	6.6958312477
O106	-5.7993014948	3.2485703570	5.5945942160
O107	-4.3153326880	1.3718933203	5.1704143938
O108	-6.4825615183	5.4272095887	6.4773634464
O109	-6.4632397129	3.4269811865	8.0306483501
S110	-6.1168021194	-1.9006293157	4.2941568555
O111	-5.6088126412	-2.7239606564	3.1976374226
O112	-6.3094813625	-2.5090488550	5.6111806436

$[Ru^{III}$−OH$]^{+}$ 6-coordinate

E (B3LYP-D3/LACVP**++ 2f(Ru))(a.u.) = -2792.756173
ZPE (kcal mol^{-1}) = 328.264
G_{solv} (kcal mol^{-1}) = -45.059
ΔH_{298} (kcal mol^{-1}) = 27.131
ΔS_{298} (cal K^{-1} mol^{-1}) = 249.11
Cartesian coordinates

Atom	x	y	z
C1	2.3958416137	2.9063064057	2.1758218724
C2	1.2940827946	3.3747781398	1.4537804010
C3	0.0418361568	3.2103194174	2.0246490375
N4	-0.1264041329	2.6127225584	3.2101750183
C5	0.9170965512	2.1568106963	3.9312516838
C6	2.2128330861	2.3035660700	3.4200076084
H7	3.3977884464	3.0126654402	1.7720927999
H8	1.3868569300	3.8456221260	0.4818699340
H9	3.0721942588	1.9520542586	3.9767091334
Ru10	-2.0364717508	2.2527938974	3.9270088488
C11	-0.1760617561	0.5527419745	7.6718218326
C12	1.1637130792	0.5430212251	7.3227560058
C13	1.5293391940	1.0654301505	6.0872567771
C14	0.5477505377	1.5613237120	5.2321409998
N15	-0.7911960923	1.5417863585	5.5660248648
C16	-1.1309450392	1.0606856678	6.7805917523
H17	-0.5312725085	0.1893901051	8.6280052179
H18	1.9135896967	0.1487810007	8.0014174825
H19	2.5707631472	1.0910922757	5.7920518065
O20	-2.5467708647	2.9342469845	1.9591549893
C21	-2.3396792690	6.7716441523	5.9964585839
C22	-1.1941458963	6.3786015473	5.2959497838
C23	-1.1318563131	5.1201522925	4.7184045630
N24	-2.1374282201	4.2242652719	4.8027003240
C25	-3.2381234239	4.5890031882	5.4900173226
C26	-3.3707613722	5.8343293991	6.0844127187
H27	-0.3517284584	7.0539169905	5.1823825855
H28	-0.2544593396	4.8240128123	4.1620145763
H29	-4.0181423388	3.8550282213	5.5646276440
H30	-4.2940949075	6.0597828516	6.6083640779
C31	-2.0274400943	-2.2816365958	1.9780671571
C32	-2.5050295156	-2.0190121655	3.2670661796
C33	-2.4662034445	-0.7304877902	3.7828237995
N34	-1.9635697057	0.3021750999	3.0737419586
C35	-1.5189649890	0.0724613060	1.8239500847
C36	-1.5303592035	-1.1926392550	1.2535839639
H37	-2.9240989568	-2.8127806764	3.8779052938
H38	-2.8561002157	-0.4986294416	4.7697187840
H39	-1.1774635487	0.9287741947	1.2581751592
H40	-1.1657150519	-1.3184303909	0.2387376225
C41	-2.4589350271	8.1315931323	6.6308156609
H42	-2.4976734046	8.0451022985	7.7226427738
H43	-1.6150525772	8.7760385049	6.3705723254
H44	-3.3819188457	8.6271457134	6.3121928650
C45	-2.0450825884	-3.6739710583	1.4044653530
H46	-1.2583093081	-4.2867972768	1.8607174990
H47	-2.9996439151	-4.1696610614	1.6064067661
H48	-1.8807781005	-3.6671579685	0.3237923573
S49	-2.8769429095	1.0015381829	7.4277513358
O50	-3.4057573303	2.3620592599	7.1781756158
O51	-3.8801190594	1.8271876265	4.2108924950
H52	-4.4975068587	2.5937071942	3.9590143982
O54	-3.4998279999	-0.0831686940	6.6192424888
O55	-2.6816794826	0.6605526742	8.8483332903
S56	-1.5076337043	3.8313809009	1.2841181960
O57	-1.3922242829	3.5870386064	-0.1541422568
O58	-1.6077800939	5.2364277727	1.7383517229

O59	-5.9753516158	0.4987445396	5.4271771593
H60	-5.3844291314	0.1423872718	6.1130165842
H61	-5.3106800495	0.7748354014	4.7681201244
O62	-6.1107821968	3.1000670733	6.3044400077
H63	-6.2579373094	2.1687965104	6.0086280401
H64	-5.3154881577	2.9945348093	6.8522840925
O65	-5.5855308368	3.7033340516	3.6560131599
H66	-5.9204946403	3.7664120341	4.5770331573
H67	-5.1301749251	4.5424957675	3.4340633714
O68	-4.1364250376	5.9547339102	2.8726558911
H69	-3.2508925945	5.6609951848	2.5867765761
H70	-4.5525226370	6.2578326614	2.0559315771

[Ru^{III}−OH]$^{+}$ 7-coordinate
E (B3LYP-D3/LACVP**++ 2f(Ru))(a.u.) = -2792.756819
ZPE (kcal mol^{-1}) = 328.368
G_{solv} (kcal mol^{-1}) = -42.223
ΔH_{298} (kcal mol^{-1}) = 28.077
ΔS_{298} (cal K^{-1} mol^{-1}) = 258.171
Cartesian coordinates

Atom	x	y	z
C1	3.0107410494	2.5529283950	1.9885957643
C2	1.8640432143	3.0764864773	1.3911432603
C3	0.6991189374	3.0728807856	2.1551005814
N4	0.6257320763	2.5842588067	3.3823564147
C5	1.7323595512	2.1248395618	3.9825871415
C6	2.9565652522	2.0873304442	3.3040555788
H7	3.9467382586	2.5163593008	1.4390340121
H8	1.8524433949	3.4685411537	0.3807591544
H9	3.8464609442	1.6924074934	3.7815162413
Ru10	-1.8911909516	2.0004656030	4.2828229033
C11	0.9185728447	0.9527886675	7.9763739791
C12	2.2526255097	1.1074008912	7.6090246197
C13	2.5649518590	1.4805714530	6.3008222442
C14	1.5261422057	1.7097557705	5.3930273933
N15	0.2381377915	1.5892213528	5.7636791255
C16	-0.0464079613	1.2080626436	7.0028172497
H17	0.6179444575	0.6245209188	8.9645625139
H18	3.0461028820	0.9285172214	8.3284251144
H19	3.5991408414	1.6006206432	5.9991006166
O20	-1.9089540947	3.0800463582	2.3374368416
C21	-2.2386777582	6.5054499304	6.2667163265
C22	-1.0469709946	6.0456547042	5.6916374964
C23	-0.9653013043	4.7510352483	5.2095779340
N24	-1.9962287334	3.8816940478	5.2923557751
C25	-3.1235947845	4.2962668178	5.8995052472
C26	-3.2842105379	5.5881630767	6.3782951896
H27	-0.1916442505	6.7052532834	5.5791806880
H28	-0.0733513486	4.3930185307	4.7161459811
H29	-3.9262687608	3.5834457704	5.9844309811
H30	-4.2556695432	5.8428935103	6.7887266531
C31	-1.2655914603	-2.1599016704	1.6844291461
C32	-0.8706520913	-2.0730551061	3.0259261585
C33	-1.0903755464	-0.9044574506	3.7388083166
N34	-1.6715323945	0.1772736518	3.1829123083
C35	-2.0780287959	0.1061302574	1.9017183706
C36	-1.8887818476	-1.0364741288	1.1325472605
H37	-0.3982333655	-2.9163415271	3.5211853322
H38	-0.8228294050	-0.8129451093	4.7835930416
H39	-2.5462435046	0.9985131144	1.5050707899
H40	-2.2295101726	-1.0418241587	0.1017283106
C41	-2.3954069733	7.9430339741	6.6805059579
H42	-3.1553448845	8.0573144810	7.4571988869
H43	-1.4527858928	8.3658671991	7.0419796966

H44	-2.7124819707	8.5357254173	5.8132774131
C45	-1.0079940642	-3.4061918626	0.8787274700
H46	0.0469209839	-3.4579251741	0.5830877733
H47	-1.2250766075	-4.3060527446	1.4629103206
H48	-1.6114507085	-3.4304268457	-0.0321462235
S49	-1.8131051211	0.9222007705	7.3279347001
O50	-2.3088893477	0.6892245051	5.8876561140
O51	-3.7634465692	1.9228425421	3.9265876866
H52	-4.1088726298	2.8377883335	3.7746512686
O54	-1.8640306311	-0.3173282590	8.1081604381
O55	-2.3861381853	2.1420107940	7.9197101222
S56	-0.8333023629	3.8679094761	1.6078069844
O57	-0.9073790769	3.7184149826	0.1503647065
O58	-0.7189583472	5.2555625150	2.1338113143
O59	-4.8810632705	4.3821906773	3.4560707973
H60	-5.3370598936	4.2829238618	2.6117453174
H61	-4.2105842228	5.1007754838	3.3178746612
O62	-3.0753782084	6.3426286771	3.0382620931
H63	-2.2041649853	5.9424601650	2.8238839091
H64	-3.2802328545	6.8822043824	2.2648538143
O65	-6.2109252329	4.4994547786	6.0620678136
H66	-5.9249767020	4.5643474721	5.1365360560
H67	-6.0595855840	3.5583986499	6.2670576762
O68	-5.2165401644	1.7926255883	6.4063076446
H69	-4.7327545726	1.5690981737	5.5880765457
H70	-4.5289448163	1.8062082121	7.0877449614

$[Ru^{IV}(bds)(pic)_2(OH)]^+$ with H_2PO4^- S_0

E (B3LYP-D3/LACVP**++ 2f(Ru))(a.u.) = -3436.313318

ZPE (kcal mol^{-1}) = 353.142

G_{solv} (kcal mol^{-1}) = -49.694

ΔH_{298} (kcal mol^{-1}) = 31.269

ΔS_{298} (cal K^{-1} mol^{-1}) = 281.372

Cartesian coordinates

Atom	x	y	z
C1	2.6367505775	4.4262535014	2.9293518333
C2	1.4822542323	4.5646758973	2.1627390717
C3	0.3746520939	3.8013337227	2.5153178934
N4	0.3537334119	2.9665817659	3.5543789173
C5	1.4939565036	2.7708477270	4.2525191598
C6	2.6522616404	3.4992987139	3.9692258677
H7	3.5246372529	5.0078704670	2.7021777279
H8	1.4322131737	5.2156134429	1.2974887453
H9	3.5531808109	3.3438066851	4.5505206711
Ru10	-1.7074303095	2.0166773821	4.1906026109
C11	0.8805432291	-0.1035227378	7.2769251726
C12	2.1906710289	0.3205389859	7.0600697858
C13	2.4438110474	1.2683161833	6.0703944745
C14	1.3834751652	1.7376747819	5.2882543775
N15	0.1276545355	1.2679700582	5.4549056180
C16	-0.1099930101	0.4134558243	6.4514636715
H17	0.6076378267	-0.7791061762	8.0794579259
H18	3.0009478668	-0.0554700426	7.6767857691
H19	3.4477916729	1.6447886942	5.9170123528
O20	-1.6944505339	2.4261668925	2.0466915242
C21	-2.3160261971	6.4013124529	6.3343782847
C22	-1.3657012458	5.4800915235	6.7903907988
C23	-1.2102635402	4.2727422504	6.1363134220
N24	-1.9274146730	3.9515679789	5.0418595527
C25	-2.8434956768	4.8251264410	4.5893768437
C26	-3.0467472515	6.0530719606	5.1992527436
H27	-0.7814630094	5.6800614603	7.6831487181
H28	-0.5208532333	3.5216515332	6.4992041648
H29	-3.4368361570	4.5101395308	3.7446587648

H30	-3.8269867391	6.7016414613	4.8194459459
C31	-1.4189033992	-2.2675107485	1.7654832832
C32	-2.5960598098	-1.8847829299	2.4164791209
C33	-2.6346090754	-0.7144503473	3.1632444592
N34	-1.5590794315	0.0820389318	3.2824444123
C35	-0.4202095228	-0.2654229716	2.6586527480
C36	-0.3103642253	-1.4227631696	1.9049973426
H37	-3.4919776554	-2.4928942602	2.3431774129
H38	-3.5284524033	-0.3818170288	3.6733365469
H39	0.4118461149	0.4211028345	2.7583911569
H40	0.6303782933	-1.6532223294	1.4144896768
C41	-2.6054635806	7.6675479105	7.0899194451
H42	-3.4340766243	7.4778458755	7.7826406557
H43	-1.7446850022	7.9990203259	7.6779022397
H44	-2.9114091220	8.4774923092	6.4216570462
C45	-1.3392354302	-3.5399579332	0.9636802971
H46	-1.1331371239	-4.3927091495	1.6221492300
H47	-2.2821744982	-3.7447442692	0.4486111058
H48	-0.5398054469	-3.4946840443	0.2187938794
S49	-1.8742476019	0.1457591584	6.7443365594
O50	-2.3497543458	1.4862311321	6.1647142963
O51	-3.4628116051	1.9771865279	3.8980136708
H52	-4.5041623235	2.3829894907	5.1888567298
O53	-2.3046142429	-1.0357231648	5.9944880785
O54	-2.0217133754	0.1224302098	8.2078479391
S55	-1.1010616402	3.7144204532	1.4736133636
O56	-0.6023947862	3.5366147113	0.1087146421
O57	-1.9362503990	4.8997426040	1.7473162602
O58	-3.2769986636	2.3828742577	9.5180496400
H59	-2.8807591170	1.5907245007	9.1194869025
H60	-3.4271071394	2.9961517859	8.7567031887
O61	-5.8735812437	2.0067028862	9.7978360276
H62	-4.8716199365	2.0542444413	9.7651701262
H63	-6.1150815675	2.5541798688	10.5554917972
O64	-4.5678219640	3.5415451670	1.8597335467
H65	-3.8651400840	4.1668758910	1.6211303926
H66	-4.0909688284	2.7905223892	2.2580699364
O67	-6.6124928292	3.9823581961	3.6325942673
H68	-5.9027298705	3.9392736581	2.9509651085
H69	-6.6201809994	3.0924338281	4.0165687780
P69	-5.3232802438	3.7076976072	6.7730386790
O70	-5.7709754418	4.9647320150	5.9039522407
O71	-4.0125152412	3.9415245908	7.4640149627
O72	-5.3178123986	2.4569857497	5.7529614798
O73	-6.5522143024	3.4000394236	7.7165000239
H74	-6.3158309971	2.8111330904	8.5072079203
H75	-6.1465756085	4.6991175914	5.0091300349

[Ru^{IV}(bds)(pic)$_2$(OH)]$^+$ with H$_2$PO4$^-$ T1
E (B3LYP-D3/LACVP**++ 2f(Ru))(a.u.) = -3436.334505
ZPE (kcal mol^{-1}) = 352.877
G$_{solv}$ (kcal mol^{-1}) = -45.741
ΔH$_{298}$ (kcal mol^{-1}) = 32.124
ΔS$_{298}$ (cal K^{-1} mol^{-1}) = 288.944
Cartesian coordinates

Atom	x	y	z
C1	2.7709570901	4.4400439967	3.2151720570
C2	1.6884030501	4.5694446129	2.3487019679
C3	0.5810524404	3.7556555881	2.5910371443
N4	0.5116464421	2.8881343295	3.5871482101
C5	1.5600033768	2.7411869405	4.4132398276
C6	2.7160871484	3.5109309368	4.2574633046
H7	3.6566394349	5.0539012310	3.0814686470
H8	1.6926571104	5.2601639341	1.5133047100

H9	3.5521949148	3.4054101883	4.9393327147
Ru10	-2.0609391132	2.0227023492	4.0375316112
C11	0.6853556991	-0.0353473617	7.5001624525
C12	2.0168397537	0.3401732873	7.3326235707
C13	2.3592792007	1.2405760599	6.3215404170
C14	1.3532572384	1.7341346959	5.4845639101
N15	0.0785024024	1.3352485456	5.6270486251
C16	-0.2385480623	0.5046887741	6.6069857129
H17	0.3590371332	-0.6924440860	8.2978943458
H18	2.7835632138	-0.0497581377	7.9952536116
H19	3.3899782572	1.5507846456	6.1936111056
O20	-1.6424267756	2.5330897229	2.0200223306
C21	-2.4681395735	6.4149110310	6.2114193790
C22	-1.5805491698	5.4393467641	6.6868392827
C23	-1.4659809326	4.2321908133	6.0258683523
N24	-2.1557552453	3.9664976065	4.8950722237
C25	-3.0012482535	4.8983174567	4.4197515228
C26	-3.1814986255	6.1194782440	5.0503762314
H27	-1.0243147479	5.5893640392	7.6061654718
H28	-0.8361923229	3.4402019622	6.4053650470
H29	-3.5613443692	4.6535018675	3.5329924096
H30	-3.9298495167	6.7827157506	4.6300910960
C31	-1.2257059386	-2.4660073078	2.1489772087
C32	-2.4412791578	-2.1688184657	2.7739245933
C33	-2.6584790578	-0.9082370650	3.3141217594
N34	-1.7276262929	0.0601368795	3.2430678707
C35	-0.5605186723	-0.1965350889	2.6195600583
C36	-0.2759045862	-1.4385072695	2.0769382071
H37	-3.2221178334	-2.9179665856	2.8538263827
H38	-3.5782048890	-0.6505880416	3.8226687715
H39	0.1374332415	0.6282560961	2.5548654279
H40	0.6798023006	-1.5981747486	1.5872338067
C41	-2.6987366041	7.6883678312	6.9771564592
H42	-3.4387824954	7.5033725021	7.7656656680
H43	-1.7835727286	8.0410082231	7.4628963955
H44	-3.0861353990	8.4820208825	6.3332387776
C45	-0.9337386871	-3.8361715928	1.5968337880
H46	-0.4884184681	-4.4689066844	2.3747147941
H47	-1.8448748163	-4.3320724366	1.2515034386
H48	-0.2252823114	-3.7890171973	0.7648001935
S49	-2.0192429013	0.1881765345	6.7541496200
O50	-2.5929597005	1.4096439974	6.0259777592
O51	-3.7716551659	1.9980623177	3.6343094894
H52	-4.9926857656	2.6873446853	4.7151853822
O53	-2.3158063946	-1.0976567542	6.1167902987
O54	-2.2830917379	0.3034298917	8.2062598709
S55	-0.8721131366	3.7639056909	1.5017800817
O56	-0.3630659370	3.5228828791	0.1506491927
O57	-1.6012750416	5.0213200558	1.7529057728
O58	-2.5745837444	3.1137993899	9.1080877318
H59	-2.2007609404	2.3220749175	8.6937851664
H60	-3.1255144598	3.5215549608	8.4005522105
O61	-4.6199489120	1.3259729976	9.4637858262
H62	-3.9722239742	2.0637431864	9.5820017510
H63	-4.0351409643	0.5936043969	9.2073600968
O64	-4.4036020361	4.0843877923	1.4808336111
H65	-3.5528796693	4.5304012772	1.3388087945
H66	-4.1577446568	3.2368088779	1.8822386589
O67	-5.7819537262	5.7409555010	3.3872334292
H68	-5.4612932788	5.2458005493	2.6111261887
H69	-6.0002989622	5.0357369996	4.0138458241
P69	-5.5230439723	3.3321679931	6.7465780837
O70	-6.9004732426	4.0951163108	7.1102881188
O71	-4.3464952877	4.0778446027	7.2973947154

O72	-5.6454419487	3.2800482224	5.1498093576
O73	-5.6813996505	1.8346231548	7.1940172078
H74	-5.3023659395	1.6403322259	8.1282770412
H75	-6.6838293537	4.9375527007	7.5313173791

[Ru^{IV}(bds)(pic)$_2$=O] S_0
E (B3LYP-D3/LACVP**++ 2f(Ru))(a.u.) = -2792.073844
ZPE (kcal mol^{-1}) = 319.431
G_{solv} (kcal mol^{-1}) = -42.009
ΔH_{298} (kcal mol^{-1}) = 28.626
ΔS_{298} (cal K^{-1} mol^{-1}) = 264.3
Cartesian coordinates

Atom	x	y	z
C1	2.4791357580	3.5949092343	1.8568549408
C2	1.2734099886	4.2076111010	1.5297818053
C3	0.1546923823	3.8530231247	2.2739569985
N4	0.1705895446	2.9999928585	3.2943062815
C5	1.3412064548	2.4182118998	3.6254493686
C6	2.5144295037	2.6862357527	2.9124901905
H7	3.3814477411	3.8156904557	1.2955400957
H8	1.1755248781	4.9108111250	0.7108781686
H9	3.4460186589	2.2039835830	3.1831440382
Ru10	-2.0383995141	2.3626276734	4.3581299247
C11	0.9927734401	0.1707960854	7.1855438501
C12	2.2061628082	0.1066578259	6.5027534017
C13	2.3521694348	0.8195750180	5.3159623274
C14	1.2707777946	1.5557927030	4.8163341598
N15	0.0764835232	1.5661066497	5.4444113255
C16	-0.0229741016	0.9200531469	6.6059156599
H17	0.8129948057	-0.3403943866	8.1241284750
H18	3.0325045361	-0.4779775914	6.8949172686
H19	3.2956578281	0.8025251434	4.7835793406
O20	-2.3023670987	3.2876050158	2.4293042020
C21	-2.0427731731	6.3179104406	7.2982299283
C22	-0.9188197228	5.9895698626	6.5294540867
C23	-0.9680903383	4.9105478255	5.6652419515
N24	-2.0673312671	4.1455366727	5.5223047260
C25	-3.1617222960	4.4479422965	6.2459053296
C26	-3.1757080208	5.5205639501	7.1306763548
H27	-0.0046559374	6.5711225761	6.6028191525
H28	-0.1098162756	4.6370164926	5.0669215536
H29	-4.0238438427	3.7971762286	6.1462430519
H30	-4.0812943825	5.7104605792	7.6981914612
C31	-2.2061797403	-1.9091486247	1.9122912712
C32	-2.9853086348	-1.6694447517	3.0500631185
C33	-2.9001863507	-0.4515948021	3.7122535390
N34	-2.0666262192	0.5227775986	3.3003733347
C35	-1.3135211705	0.3138183963	2.2032243851
C36	-1.3607361695	-0.8757596994	1.4921271754
H37	-3.6718788725	-2.4242583458	3.4210632230
H38	-3.4961219954	-0.2312796024	4.5882425483
H39	-0.6923993179	1.1296283828	1.8585313560
H40	-0.7489579582	-0.9717667911	0.6007072704
C41	-2.0035918599	7.4575955264	8.2820500561
H42	-1.4956525260	7.1457978673	9.2030373690
H43	-1.4510773618	8.3122073767	7.8785552198
H44	-3.0086557340	7.7904547768	8.5529312878
C45	-2.2612146703	-3.2229444592	1.1782401543
H46	-1.4405480003	-3.8748758787	1.5018874977
H47	-3.1989991312	-3.7513001851	1.3695535387
H48	-2.1570070783	-3.0777061374	0.0989691276
S49	-1.6054943572	1.1827258785	7.4073185523
O50	-2.4983796990	1.2242272295	6.1679529328
O51	-3.7909163745	2.3495013771	4.3174067451

O52	-1.9012159185	0.0165978510	8.2523505581
O53	-1.4827438797	2.4858412726	8.0907784281
S54	-1.4902771647	4.4980089426	1.9347194295
O55	-1.6014317056	4.6231504744	0.4691866362
O56	-1.6569877329	5.7034624186	2.7483002185
O57	-3.9807891379	2.0294902592	9.4616127586
H58	-3.1709176723	2.5145673846	9.2334043776
H59	-3.6671388750	1.1143347323	9.5005505308
O60	-5.3378883771	2.2946471617	6.9738159035
H61	-4.9401378743	2.1900242067	7.8603853191
H62	-4.7432768591	1.8045010215	6.3909954116
O63	-3.0229046633	2.0076795064	-0.0054507227
H64	-3.0356504560	2.1673252651	0.9537405044
H65	-3.0275853316	2.9157081678	-0.3437929651
O66	-0.2533975728	1.9579188432	-0.1932069768
H67	-0.2269630870	2.9057713515	-0.3775198806
H68	-1.2211050660	1.7916056225	-0.2188052469

[Ru^{IV}(bds)(pic)$_2$=O] T$_1$
E (B3LYP-D3/LACVP**++ 2f(Ru))(a.u.) = -2792.11025
ZPE (kcal mol^{-1}) = 320.339
G_{solv} (kcal mol^{-1}) = -40.18
ΔH_{298} (kcal mol^{-1}) = 27.138
ΔS_{298} (cal K^{-1} mol^{-1}) = 247.574
Cartesian coordinates

Atom	x	y	z
C1	2.4885918750	3.0075727983	1.5996878677
C2	1.3589972467	3.7682709230	1.3025223808
C3	0.3059891721	3.7058333101	2.2097917531
N4	0.3207426028	2.9797755853	3.3159614728
C5	1.4023016487	2.2454388652	3.6090581714
C6	2.5172429027	2.2354013631	2.7620976678
H7	3.3380740794	3.0019569574	0.9236748833
H8	1.2728232022	4.3652333755	0.4027734666
H9	3.3868197529	1.6294403895	2.9898138968
Ru10	-2.2613769791	2.4572019054	4.3480393929
C11	0.8481910695	0.1159611817	7.2406197895
C12	2.0855265858	0.0035867535	6.6083481388
C13	2.3129465005	0.6911496653	5.4163692542
C14	1.2847004525	1.4701857833	4.8706113349
N15	0.0919970187	1.5700807231	5.4801931005
C16	-0.1001717223	0.9245019772	6.6204821232
H17	0.6061599752	-0.3982957325	8.1632654043
H18	2.8691285674	-0.6126508207	7.0388986364
H19	3.2752134292	0.6188859671	4.9225550744
O20	-2.2424315077	3.4827260198	2.4623702847
C21	-1.9265765234	6.2900856839	7.3946170602
C22	-0.8576327423	5.9071891774	6.5769863411
C23	-1.0143868125	4.8676690974	5.6772412610
N24	-2.1734656614	4.1912147855	5.5567704329
C25	-3.2181024142	4.5389014986	6.3353244186
C26	-3.1224241151	5.5826955105	7.2465038152
H27	0.0985374567	6.4182229119	6.6334808234
H28	-0.2076058374	4.5581512618	5.0289802971
H29	-4.1251124320	3.9423985404	6.2613511163
H30	-3.9906061975	5.8223266021	7.8524729185
C31	-1.6830616241	-1.8376063481	1.9453470729
C32	-2.3549941103	-1.7643401830	3.1742790711
C33	-2.5275248222	-0.5419953928	3.8050617535
N34	-2.0592429817	0.6073656245	3.2725887721
C35	-1.4282459036	0.5562057677	2.0850232491
C36	-1.2240101140	-0.6350536367	1.4028795769
H37	-2.7511548263	-2.6599715363	3.6433586580
H38	-3.0337242591	-0.4555642302	4.7584198115

H39	-1.0986905135	1.4844434729	1.6441006537
H40	-0.7216397402	-0.5918466773	0.4418340713
C41	-1.7729301606	7.3895268955	8.4119322049
H42	-1.3469802118	6.9868040547	9.3390833064
H43	-1.0975795045	8.1727871009	8.0542848014
H44	-2.7349883476	7.8451303848	8.6605674668
C45	-1.4583058774	-3.1582540839	1.2573828108
H46	-0.6008601111	-3.6786817616	1.7021469709
H47	-2.3272095165	-3.8147143550	1.3645645213
H48	-1.2514407336	-3.0252908390	0.1924748550
S49	-1.7225908194	1.2304320828	7.3487670926
O50	-2.6125245369	1.2926605688	6.0994823879
O51	-3.9996132450	2.5111536117	4.1900791123
O52	-2.0704952010	0.0604166256	8.1705710992
O53	-1.6072094561	2.5261846293	8.0462068946
S54	-1.2621289639	4.5796633030	1.9876265280
O55	-1.4218833581	4.8052200570	0.5404544364
O56	-1.2306735441	5.7441050077	2.8757398400
O57	-4.1798147438	2.0090874599	9.3007201474
H58	-3.3699051280	2.5169235214	9.1305051501
H59	-3.8499877021	1.0985316959	9.3131279130
O60	-5.4747750715	2.4528308846	6.8164915199
H61	-5.0954324629	2.2510553598	7.6950080854
H62	-4.9429578977	1.9344946034	6.1981014084
O63	-3.4923887938	2.0568612901	0.3743727845
H64	-3.2303914398	2.5862602946	1.1515574717
H65	-4.0304779010	2.6624104433	-0.1506879506
O66	-0.8387486775	2.1257976268	-0.5030367602
H67	-0.8287451784	3.0944612606	-0.4843749615
H68	-1.7940242103	1.9503774157	-0.3770238885

Reactant state of WNA from $Ru^{IV}=O_{S0}$ species under buffer solvent

E (B3LYP-D3/LACVP**++ 2f(Ru))(a.u.) = -3435.763654

ZPE (kcal mol^{-1}) = 345.256

G_{solv} (kcal mol^{-1}) = -97.762

ΔH_{298} (kcal mol^{-1}) = 31.568

ΔS_{298} (cal K^{-1} mol^{-1}) = 284.98

Cartesian coordinates

Atom	x	y	z
C1	2.4033237611	4.6240621266	3.2579360978
C2	1.3133208155	4.6945640359	2.3953840972
C3	0.2626670356	3.8059916759	2.6044025834
N4	0.2347191237	2.9135394790	3.5923447158
C5	1.3146627227	2.8028182502	4.3959563121
C6	2.4158050353	3.6521159647	4.2559684308
H7	3.2428641272	5.3035416145	3.1463305591
H8	1.2672105986	5.3958322512	1.5703651100
H9	3.2646826910	3.5639504195	4.9234657459
Ru10	-1.8924946192	1.8455005742	4.0607074144
C11	0.6864057002	-0.1304977431	7.3622804928
C12	1.9645637389	0.4165934253	7.2698626651
C13	2.2241960672	1.3753931002	6.2929101915
C14	1.2039641561	1.7438460826	5.4091623313
N15	-0.0127034099	1.1651163758	5.4626690646
C16	-0.2595926069	0.2869503577	6.4341006215
H17	0.3968954009	-0.8239755924	8.1431665518
H18	2.7416091601	0.1269778232	7.9708595323
H19	3.1999263696	1.8432037369	6.2311415944
O20	-1.7261340787	2.3736796947	1.9418917235
C21	-2.5748211114	6.2153938089	6.2029987294
C22	-1.6239957081	5.3012374272	6.6788108921
C23	-1.4769612266	4.0751605798	6.0623239151
N24	-2.2000522625	3.7321384896	4.9728975171
C25	-3.1160766840	4.5998485365	4.5061439349

C26	-3.3206736738	5.8418155012	5.0880355769
H27	-1.0566564284	5.5082333755	7.5808975253
H28	-0.8192569835	3.3208153391	6.4696993643
H29	-3.7186387847	4.2853889438	3.6703148651
H30	-4.1223499740	6.4446823443	4.6776192609
C31	-1.1809061949	-2.4021369933	1.6482595007
C32	-2.4349502312	-2.0709304259	2.1693832296
C33	-2.6049625821	-0.8912852053	2.8846651279
N34	-1.5889189086	-0.0391761399	3.0893380713
C35	-0.3813519679	-0.3311199402	2.5806307067
C36	-0.1381033625	-1.4940660002	1.8668028619
H37	-3.2855057073	-2.7300854872	2.0267992156
H38	-3.5582640753	-0.5930216084	3.3019710127
H39	0.3979301579	0.4026787393	2.7491590186
H40	0.8566194436	-1.6826229942	1.4735725105
C41	-2.8847060895	7.4741612088	6.9588356372
H42	-3.6883668048	7.2250106395	7.6616424584
H43	-2.0238220795	7.8371352316	7.5302995248
H44	-3.2407390318	8.2699112651	6.2984079723
C45	-0.9483540725	-3.6914183773	0.9028006065
H46	-0.7051140135	-4.4996595437	1.6038618753
H47	-1.8386716220	-3.9971246001	0.3455891253
H48	-0.1141619271	-3.6015570826	0.2002417106
S49	-1.9996514093	-0.1871374293	6.5196878785
O50	-2.5666569628	1.1118167822	5.9376757890
O51	-3.6093908347	1.7781507756	3.6655640342
O53	-2.1758011945	-1.3738224401	5.6715072038
O54	-2.3214643895	-0.3151967999	7.9461707060
S55	-1.1268796590	3.6862073238	1.4518387884
O56	-0.5094395402	3.5522178540	0.1258764126
O57	-1.9994346434	4.8520672748	1.6752606378
O58	-2.5421593475	2.6236907649	8.7136411887
H59	-2.8301252943	1.7338264089	8.4608332092
H60	-3.0873343273	3.1896649331	8.1364107640
O61	-4.6794473571	3.7336035530	10.4258348608
H62	-3.9662204861	3.1571207569	10.0949035067
H63	-5.4683751387	3.4708896738	9.8930484528
O64	-4.7211214544	3.5472389405	1.6478050566
H65	-3.9393567320	4.0950993577	1.4763802756
H66	-4.3670066067	2.7985186642	2.1561820154
O67	-5.9141672545	5.6490121938	3.4099469495
H68	-5.7133136187	4.8941646342	2.8335939872
H69	-6.0699581348	5.2518168742	4.2926129875
P69	-5.6303359406	3.9134978754	7.2407111910
O70	-4.8555751945	2.5193885422	6.7967720923
O71	-6.5461028725	3.5593691913	8.3870468955
O72	-6.0588182152	4.6327181203	5.9912414000
O73	-4.3605087313	4.7642856732	7.9167415853
H74	-4.4609472258	4.6922405571	8.8947954584
H75	-4.6161445895	2.5325554013	5.8589620401

Transition state of WNA from Ru^{IV}=O_{S0} species under buffer solvent

E (B3LYP-D3/LACVP**++ 2f(Ru))(a.u.) = -3435.769485

ZPE (kcal mol^{-1}) = 344.694

G_{solv} (kcal mol^{-1}) = -75.338

ΔH_{298} (kcal mol^{-1}) = 30.742

ΔS_{298} (cal K^{-1} mol^{-1}) = 276.765

Cartesian coordinates

Atom	x	y	z
C1	2.0854200000000	5.3013520000000	3.5781700000000
C2	1.1418700000000	5.1266070000000	2.5667180000000
C3	0.4033440000000	3.9448830000000	2.5891130000000
N4	0.5411060000000	3.0003480000000	3.5047250000000
C5	1.4221070000000	3.1783680000000	4.4937960000000

C6	2.2295640000000	4.3215260000000	4.5616650000000
H7	2.6847670000000	6.2066210000000	3.6209580000000
H8	0.9467600000000	5.8687120000000	1.8016340000000
H9	2.9187700000000	4.4679960000000	5.3864390000000
Ru10	-2.0257130000000	1.9184930000000	4.2149050000000
C11	1.0017250000000	0.3129180000000	7.5763960000000
C12	2.2005230000000	1.0174490000000	7.5142770000000
C13	2.3918040000000	1.9435010000000	6.4892040000000
C14	1.3754890000000	2.1335160000000	5.5455510000000
N15	0.2388580000000	1.4115190000000	5.5733570000000
C16	0.0660950000000	0.5503480000000	6.5701510000000
H17	0.7644640000000	-0.3808960000000	8.3742850000000
H18	2.9717280000000	0.8626920000000	8.2637140000000
H19	3.3125100000000	2.5123330000000	6.4246710000000
O20	-1.9811670000000	2.9916600000000	2.2787270000000
C21	-1.9550710000000	6.1470580000000	6.7466100000000
C22	-1.7460340000000	4.9195200000000	7.3809650000000
C23	-1.7860950000000	3.7453940000000	6.6447310000000
N24	-2.0208630000000	3.7326400000000	5.3185820000000
C25	-2.2425700000000	4.9074880000000	4.6945560000000
C26	-2.1972560000000	6.1182800000000	5.3706930000000
H27	-1.6036860000000	4.8596870000000	8.4554870000000
H28	-1.6911950000000	2.7869440000000	7.1324950000000
H29	-2.4913500000000	4.8435340000000	3.6439070000000
H30	-2.3902390000000	7.0352470000000	4.8215770000000
C31	-1.7723280000000	-2.2444140000000	1.6235790000000
C32	-2.7935930000000	-2.0321370000000	2.5546740000000
C33	-2.8468190000000	-0.8421870000000	3.2663620000000
N34	-1.9434120000000	0.1400930000000	3.0890330000000
C35	-0.9620740000000	-0.0411600000000	2.1844020000000
C36	-0.8488940000000	-1.2096190000000	1.4444880000000
H37	-3.5448850000000	-2.7940540000000	2.7389760000000
H38	-3.6114640000000	-0.6493500000000	4.0087810000000
H39	-0.2747100000000	0.7826470000000	2.0456330000000
H40	-0.0431150000000	-1.2992220000000	0.7218930000000
C41	-2.0076690000000	7.4317270000000	7.5305800000000
H42	-3.0220440000000	7.5788480000000	7.9208760000000
H43	-1.3272840000000	7.4104350000000	8.3879320000000
H44	-1.7611270000000	8.2975760000000	6.9079770000000
C45	-1.6522020000000	-3.5441420000000	0.8690620000000
H46	-1.1068360000000	-4.2855570000000	1.4671450000000
H47	-2.6357430000000	-3.9672010000000	0.6414770000000
H48	-1.1070200000000	-3.4119640000000	-0.0706210000000
S49	-1.5425890000000	-0.2799670000000	6.5871740000000
O50	-2.4422970000000	0.8058570000000	6.0121840000000
O51	-3.8952960000000	1.9221170000000	4.0462940000000
O53	-1.4123450000000	-1.4767550000000	5.7423620000000
O54	-1.8526370000000	-0.5062640000000	8.0171890000000
S55	-0.8897630000000	3.5766530000000	1.3828630000000
O56	-0.3173120000000	2.5723490000000	0.4643230000000
O57	-1.2865300000000	4.8719660000000	0.8060110000000
O58	-3.2570000000000	1.9723890000000	8.6581630000000
H59	-2.8808250000000	1.0840480000000	8.7585180000000
H60	-3.7711450000000	1.9153080000000	7.8267390000000
O61	-4.6310160000000	4.2335640000000	9.4257930000000
H62	-4.1079840000000	3.4048820000000	9.3777170000000
H63	-5.5390790000000	3.9377090000000	9.2141010000000
O64	-4.3732450000000	1.7215190000000	1.3137000000000
H65	-3.4808140000000	2.1041760000000	1.2692860000000
H66	-4.3697480000000	1.3819440000000	2.2266970000000
O67	-4.5824190000000	3.5654490000000	3.4671120000000
H68	-4.7176040000000	3.2001580000000	2.5692910000000
H69	-5.8069840000000	3.7051710000000	4.3957060000000
P69	-5.8721930000000	3.5503100000000	6.5646110000000

O70	-4.9435490000000	2.2372360000000	6.4203050000000
O71	-6.8156230000000	3.4113140000000	7.7128700000000
O72	-6.5059670000000	3.8003470000000	5.1398960000000
O73	-4.8460020000000	4.7826860000000	6.7713540000000
H74	-4.5833070000000	4.7738570000000	7.7277110000000
H75	-4.5265260000000	2.1175010000000	5.5083250000000

Product state of WNA from Ru^{IV}=O_{S0} species under buffer solvent

E (B3LYP-D3/LACVP**++ 2f(Ru))(a.u.) = -3435.784086

ZPE (kcal mol^{-1}) = 346.071

G_{solv} (kcal mol^{-1}) = -71.783

ΔH_{298} (kcal mol^{-1}) = 30.933

ΔS_{298} (cal K^{-1} mol^{-1}) = 279.295

Cartesian coordinates

Atom	x	y	z
C1	1.8710726085	5.4271795263	3.6303955429
C2	1.0337658993	5.1872715847	2.5394908893
C3	0.4603331919	3.9207606176	2.4500301667
N4	0.6693383130	2.9475901661	3.3215894157
C5	1.3992987273	3.2028575417	4.4062562105
C6	2.0418256477	4.4339524280	4.5965257536
H7	2.3432247770	6.3974559035	3.7584447083
H8	0.7895013581	5.9416399467	1.8008370565
H9	2.6073847742	4.6303488931	5.5015776366
Ru10	-1.9768738050	1.8947027340	4.3100291903
C11	0.9762787068	0.3184705781	7.4578110471
C12	2.1851177573	0.9999607306	7.3793267249
C13	2.3576360902	1.9309544107	6.3579155500
C14	1.3266159500	2.1473769219	5.4404314472
N15	0.1620619790	1.4527904445	5.4810087196
C16	0.0136635256	0.5804106994	6.4841898180
H17	0.7459927054	-0.3818896060	8.2520071616
H18	2.9729103487	0.8221955304	8.1057237091
H19	3.2827642754	2.4892935937	6.2636304354
O20	-1.9066096624	2.9895706114	2.2582330102
C21	-1.8802889922	6.1438623315	6.8013185468
C22	-1.7450543283	4.9174371674	7.4579139317
C23	-1.8039986398	3.7351858004	6.7354692737
N24	-1.9801525917	3.7109791089	5.3994210918
C25	-2.1253798463	4.8862307889	4.7548551073
C26	-2.0666195149	6.1047354246	5.4167328964
H27	-1.6465022661	4.8692835918	8.5379734202
H28	-1.7730788286	2.7797214609	7.2391104701
H29	-2.3064873329	4.8157709604	3.6895497100
H30	-2.1961590970	7.0211323079	4.8480979230
C31	-1.7347672975	-2.2306966113	1.6733698857
C32	-2.7980492497	-2.0031937726	2.5519820591
C33	-2.8532753480	-0.8240839113	3.2850192426
N34	-1.9128330591	0.1335133353	3.1770579646
C35	-0.8910250763	-0.0627017158	2.3178825610
C36	-0.7740587893	-1.2194465275	1.5628354999
H37	-3.5792322726	-2.7462625369	2.6826660560
H38	-3.6478287206	-0.6230635086	3.9940942040
H39	-0.1726209616	0.7424009821	2.2388854456
H40	0.0659322849	-1.3201550456	0.8817114154
C41	-1.9077572896	7.4387976380	7.5699686678
H42	-2.9077054207	7.5951308876	7.9923989775
H43	-1.2003437332	7.4264268602	8.4057985582
H44	-1.6772053839	8.2958010815	6.9297132066
C45	-1.6002559984	-3.5187211601	0.9010497173
H46	-0.9404725031	-4.2175524965	1.4315226854
H47	-2.5671022331	-4.0134871721	0.7695491309
H48	-1.1634296481	-3.3454524762	-0.0879802272
S49	-1.5865650583	-0.2611061702	6.6045964200

O50	-2.5280118814	0.8017113707	6.0521917743
O51	-3.9913513986	1.9934166356	4.0523905864
O53	-1.4922599118	-1.4832913261	5.7935832097
O54	-1.8168491461	-0.4493453813	8.0568305397
S55	-0.8399311600	3.4849151551	1.2781375185
O56	-0.3154521260	2.4042411039	0.4205618380
O57	-1.2364446815	4.7429038967	0.6185642488
O58	-3.3707000719	1.9016213440	8.7216847283
H59	-2.8746460416	1.0696515202	8.7807094992
H60	-3.8632764529	1.8258047720	7.8809106604
O61	-4.5283843394	4.2872617432	9.3834300873
H62	-4.0416599231	3.4346204435	9.3542488077
H63	-5.4572056682	4.0185375445	9.2493516467
O64	-4.2575425876	1.8270395704	1.2975624414
H65	-3.3302064004	2.1323124131	1.3226408679
H66	-4.2869982140	1.2590649875	2.0851173094
O67	-4.4619354186	3.2962059085	3.5417429244
H68	-4.5332453187	3.0623844115	2.5838608496
H69	-5.8568192047	3.6624795805	4.4372843732
P69	-5.8809579869	3.5567763265	6.5865223269
O70	-5.0124333189	2.2109696587	6.4388953470
O71	-6.8287120713	3.4771810268	7.7331296125
O72	-6.5321946544	3.8106683484	5.1540834977
O73	-4.8202696483	4.7615975073	6.7368137883
H74	-4.5252825238	4.7663340064	7.6853641205
H75	-4.5758087815	2.0815373999	5.5317546604

Reactant state of WNA from Ru^{IV}=O_{T1} species under buffer solvent

E (B3LYP-D3/LACVP**++ 2f(Ru))(a.u.) = -3435.803335

ZPE (kcal mol^{-1}) = 345.097

G_{solv} (kcal mol^{-1}) = -92.655

ΔH_{298} (kcal mol^{-1}) = 31.606

ΔS_{298} (cal K^{-1} mol^{-1}) = 285.23

Cartesian coordinates

Atom	x	y	z
C1	2.2735436296	2.9301090733	1.5305676900
C2	1.0974966134	3.6346714977	1.2765327666
C3	0.1164114387	3.5949675986	2.2657028602
N4	0.2425361221	2.9336638455	3.4059495442
C5	1.3683758696	2.2527437845	3.6572928513
C6	2.4184307398	2.2320259872	2.7310624702
H7	3.0733169639	2.9140370304	0.7955446530
H8	0.9138761020	4.1748516019	0.3554374726
H9	3.3268881619	1.6757163132	2.9309905111
Ru10	-2.1970591963	2.3592023839	4.6138655206
C11	1.1304708940	0.3554636512	7.4479401892
C12	2.3014379311	0.1784088118	6.7132144277
C13	2.4315239574	0.7857678785	5.4628334677
C14	1.3706757998	1.5545602247	4.9697523565
N15	0.2446166293	1.7175171847	5.6804885464
C16	0.1376233102	1.1452497515	6.8701743261
H17	0.9591017375	-0.1025898081	8.4146877674
H18	3.1091594752	-0.4331584126	7.1052466486
H19	3.3381544972	0.6529431205	4.8829473603
O20	-2.4045240618	3.3384410983	2.6852797121
C21	-2.3967249911	6.6806306322	6.9373271260
C22	-1.1775050465	6.1349211822	6.5221714270
C23	-1.1548933472	4.9169054868	5.8649962666
N24	-2.2778503831	4.2205794780	5.6195649722
C25	-3.4593714080	4.7213259959	6.0231942916
C26	-3.5538908035	5.9459044125	6.6670032386
H27	-0.2437557632	6.6604768281	6.6955359112
H28	-0.2308709284	4.4753315803	5.5137483097
H29	-4.3362415811	4.1271319375	5.8073439266

H30	-4.5452360823	6.3087082277	6.9148159754
C31	-2.5680820415	-1.8840725651	2.2218529579
C32	-2.7094780097	-1.8147271637	3.6082975944
C33	-2.4547616310	-0.6277965154	4.2762780095
N34	-2.0267819678	0.4771242712	3.6286376509
C35	-1.8227632476	0.4077654962	2.2946018785
C36	-2.0579410780	-0.7494057933	1.5759485001
H37	-3.1538461584	-2.6335957315	4.1606414876
H38	-2.6673361786	-0.5212009530	5.3295196783
H39	-1.5295978711	1.3270966784	1.8062185065
H40	-1.9299551323	-0.7365189925	0.4978812182
C41	-2.4534878856	7.9964324302	7.6696677162
H42	-1.6206442412	8.6485826258	7.3884125921
H43	-3.3911179459	8.5227911914	7.4692235556
H44	-2.3931148859	7.8311252522	8.7525220899
C45	-3.0958167455	-3.0657260542	1.4654974048
H46	-2.8080982869	-4.0122920894	1.9359145735
H47	-4.1912665026	-2.9779801876	1.5220531212
H48	-2.7714318584	-3.0702277908	0.4203415819
S49	-1.4361794675	1.4994627151	7.6913551323
O50	-2.3957485955	1.3523216167	6.4928700251
O51	-3.9384235578	2.2889767117	4.5363132497
O52	-1.6460468714	0.4285889686	8.6703514728
O53	-1.3308863947	2.8831342934	8.1769038320
S54	-1.4822608243	4.4310354473	2.1135804208
O55	-1.7106654801	4.6211459818	0.6765744632
O56	-1.4021977027	5.6205342289	2.9743525705
O57	-5.5128978219	1.3344704765	6.6491367768
H58	-4.5792071977	1.5773652630	6.7248858869
H59	-5.4592253965	0.5231580383	6.0507619967
O60	-6.7619522553	2.6218203755	4.7008038423
H61	-7.1118079837	3.4351602514	5.0980637495
H62	-6.2439825305	2.1947937325	5.4463251601
O63	-6.4497581335	5.5100868758	5.4167399089
H64	-5.9019748492	5.2701695500	4.6261480274
H65	-7.1458562068	6.0688051299	5.0510947881
O66	-5.3036361186	4.4294515564	3.2465830969
H67	-5.7197021238	3.5958676319	3.5675373694
H68	-4.3789009782	4.1912427444	3.0844628816
P69	-5.7864165440	-0.6472321788	3.4908990082
O70	-4.9594563811	0.4655388344	2.5851347633
O71	-5.2207024762	-0.5671063782	4.9109249128
O72	-5.7632733140	-1.9507577760	2.7500417766
O73	-7.3059090012	-0.0324948715	3.4943102824
H74	-7.2977003618	0.8630675389	3.8829425670
H75	-4.6924391400	1.1944128118	3.1677995754

Transition state of WNA from $Ru^{IV}=O_{T1}$ species under buffer solvent

E (B3LYP-D3/LACVP**++ 2f(Ru))(a.u.) = -3435.731723

ZPE (kcal mol^{-1}) = 343.339

G_{solv} (kcal mol^{-1}) = -87.51

ΔH_{298} (kcal mol^{-1}) = 29.892

ΔS_{298} (cal K^{-1} mol^{-1}) = 269.568

Cartesian coordinates

Atom	x	y	z
C1	2.3490710000000	2.8248420000000	1.5629270000000
C2	1.2021470000000	3.5424860000000	1.2204270000000
C3	0.2008520000000	3.6238170000000	2.1855090000000
N4	0.2825440000000	3.0748070000000	3.3866540000000
C5	1.3657550000000	2.3562630000000	3.7104460000000
C6	2.4348190000000	2.2125270000000	2.8140970000000
H7	3.1627920000000	2.7194730000000	0.8506030000000
H8	1.0552240000000	3.9994630000000	0.2490250000000
H9	3.3045480000000	1.6185680000000	3.0724100000000

Ru10	-2.3590150000000	2.4766440000000	4.4511570000000
C11	0.9000040000000	0.5458380000000	7.5196950000000
C12	2.1284980000000	0.3923190000000	6.8811240000000
C13	2.3374740000000	0.9878990000000	5.6362910000000
C14	1.2927270000000	1.7124650000000	5.0491990000000
N15	0.1132680000000	1.8587740000000	5.6704630000000
C16	-0.0640400000000	1.3065090000000	6.8589330000000
H17	0.6688600000000	0.0980150000000	8.4788750000000
H18	2.9209210000000	-0.1877720000000	7.3455950000000
H19	3.2936260000000	0.8832700000000	5.1353240000000
O20	-2.3364480000000	3.2840840000000	2.4132390000000
C21	-2.2591330000000	6.5116440000000	7.2490630000000
C22	-1.1777510000000	6.1817320000000	6.4262370000000
C23	-1.2616900000000	5.0912250000000	5.5746120000000
N24	-2.3594010000000	4.3156170000000	5.5128180000000
C25	-3.4059170000000	4.6162840000000	6.3018500000000
C26	-3.3981030000000	5.7077550000000	7.1568060000000
H27	-0.2655200000000	6.7709570000000	6.4440900000000
H28	-0.4487660000000	4.8157580000000	4.9179540000000
H29	-4.2574600000000	3.9563290000000	6.2380500000000
H30	-4.2800170000000	5.9100170000000	7.7552310000000
C31	-2.4886750000000	-1.9585020000000	2.3462300000000
C32	-2.5928540000000	-1.7995270000000	3.7277760000000
C33	-2.4224630000000	-0.5469590000000	4.2957070000000
N34	-2.1105020000000	0.5410290000000	3.5638700000000
C35	-1.9140040000000	0.3821700000000	2.2360330000000
C36	-2.0774420000000	-0.8400510000000	1.6090470000000
H37	-2.9500930000000	-2.6069330000000	4.3556560000000
H38	-2.6174550000000	-0.3830240000000	5.3450140000000
H39	-1.6940300000000	1.2821740000000	1.6780800000000
H40	-1.9716500000000	-0.8972070000000	0.5297090000000
C41	-2.1810440000000	7.6581520000000	8.2241340000000
H42	-1.4748260000000	8.4241760000000	7.8887960000000
H43	-3.1587890000000	8.1271670000000	8.3707140000000
H44	-1.8390610000000	7.2977370000000	9.2025380000000
C45	-2.9587700000000	-3.2238020000000	1.6911140000000
H46	-2.5999540000000	-4.1144080000000	2.2189130000000
H47	-4.0566750000000	-3.2064520000000	1.7630340000000
H48	-2.6579060000000	-3.2842820000000	0.6404930000000
S49	-1.6655220000000	1.6752990000000	7.6201380000000
O50	-2.6194870000000	1.5761570000000	6.4160350000000
O51	-4.1973370000000	2.4352550000000	4.0959630000000
O52	-1.9276690000000	0.5939720000000	8.5796660000000
O53	-1.5405590000000	3.0450690000000	8.1442010000000
S54	-1.4197010000000	4.3726500000000	1.8641880000000
O55	-1.5176510000000	4.5072530000000	0.4046400000000
O56	-1.5026310000000	5.6130390000000	2.6730420000000
O57	-5.3560610000000	0.9802560000000	6.7426180000000
H58	-4.4043480000000	1.0991630000000	6.9035000000000
H59	-5.3659930000000	0.1614440000000	6.1153360000000
O60	-5.7415530000000	2.4573380000000	4.8017350000000
H61	-5.9426460000000	3.4046070000000	5.0113780000000
H62	-5.5976460000000	1.8954340000000	5.6965430000000
O63	-6.1653940000000	5.1259560000000	5.1612300000000
H64	-5.4881280000000	5.3109640000000	4.4538620000000
H65	-7.0123380000000	5.2832930000000	4.7250970000000
O66	-4.4628310000000	5.1928440000000	3.1424360000000
H67	-4.3314140000000	4.2269990000000	3.0906850000000
H68	-3.5546800000000	5.5384240000000	3.1563890000000
P69	-5.7741920000000	-0.8954020000000	3.6009970000000
O70	-4.9702250000000	0.1723150000000	2.6492380000000
O71	-5.1690640000000	-0.8349050000000	5.0092310000000
O72	-5.8482780000000	-2.2061560000000	2.8838350000000
O73	-7.2720160000000	-0.1997810000000	3.6831140000000

H74 -7.1446400000000 0.7026140000000 4.0168300000000
H75 -4.7247560000000 0.9813540000000 3.1410220000000

Product state of WNA from Ru^{IV}=O_{T1} species under buffer solvent

E (B3LYP-D3/LACVP**++ 2f(Ru))(a.u.) = -3435.767197

ZPE (kcal mol^{-1}) = 345.547

G_{solv} (kcal mol^{-1}) = -81.328

ΔH_{298} (kcal mol^{-1}) = 31.366

ΔS_{298} (cal K^{-1} mol^{-1}) = 286.349

Cartesian coordinates

Atom	x	y	z
C1	1.9339091451	2.1418043273	1.4579875016
C2	0.9680026742	3.0530023374	1.0424475194
C3	-0.0025797215	3.4455674533	1.9645285654
N4	-0.0403038135	3.0231142737	3.2304588586
C5	0.9197287963	2.1774013411	3.6406843638
C6	1.9106267043	1.6918260663	2.7743367237
H7	2.6880253556	1.7765335798	0.7661604223
H8	0.9210296388	3.4510498758	0.0362271991
H9	2.6269497897	0.9544068370	3.1185998859
Ru10	-2.2513163004	2.3306480377	4.6603896715
C11	0.8165049454	0.8824558269	7.6579036129
C12	2.0480200092	0.9828387627	7.0281671053
C13	2.0804686230	1.4343687603	5.7134947500
C14	0.8882252965	1.7642759148	5.0611013323
N15	-0.3147318354	1.7192706169	5.6934054901
C16	-0.3268803404	1.2767639188	6.9626937424
H17	0.7040277252	0.5051403974	8.6671985170
H18	2.9652962298	0.7150607974	7.5447893887
H19	3.0234965310	1.5467852540	5.1914146082
O20	-2.5491529893	3.6320541224	1.6089186908
C21	-2.2493741734	6.5222415919	7.2735602008
C22	-1.4527595680	6.3550781264	6.1330079590
C23	-1.5265352404	5.1803224695	5.4037083757
N24	-2.3479294637	4.1646301667	5.7532017575
C25	-3.1381657124	4.3234412081	6.8290020979
C26	-3.1132022691	5.4784833149	7.6028505776
H27	-0.7786293985	7.1399818092	5.8019319713
H28	-0.9454221554	5.0387551568	4.5019808852
H29	-3.7872491549	3.4925793786	7.0661620785
H30	-3.7657589682	5.5452753163	8.4671333850
C31	-2.0845904728	-1.9234414848	2.1563036971
C32	-2.2525173104	-1.8814775626	3.5424080453
C33	-2.2647246354	-0.6612753529	4.2017218515
N34	-2.1076576903	0.5082232130	3.5529338712
C35	-1.9573312526	0.4866817206	2.2126354182
C36	-1.9237109302	-0.7007011219	1.4962295175
H37	-2.4173369880	-2.7900191303	4.1130285812
H38	-2.4375068839	-0.5934830637	5.2690564604
H39	-1.9129890963	1.4534545363	1.7229445515
H40	-1.8141389243	-0.6635729330	0.4165219488
C41	-2.1447569696	7.7707905024	8.1120150017
H42	-2.0398930704	8.6622698367	7.4844779701
H43	-3.0198103794	7.9006453184	8.7546944882
H44	-1.2598137263	7.7237658221	8.7594742669
C45	-2.1513728604	-3.2272496830	1.4051122933
H46	-1.7062489783	-4.0452876121	1.9810941624
H47	-3.1995960471	-3.4895299046	1.2175857013
H48	-1.6436380763	-3.1644983196	0.4378571817
S49	-1.8905872770	1.1981990933	7.8977894946
O50	-2.9220882291	1.1197844591	6.8004479041
O51	-4.1133074210	2.4004695447	3.9765955370
O52	-1.7743163185	-0.0436729101	8.6843442630
O53	-1.9290167198	2.4587347457	8.6650252819

S54	-1.3626939322	4.5197052102	1.4148617245
O55	-1.0333753971	4.8474363647	0.0133319355
O56	-1.3356628283	5.6809934210	2.3581064874
O57	-5.5624971347	0.6057939159	6.9119475972
H58	-4.6202282000	0.5759975768	7.1674715865
H59	-5.4663267196	-0.4400801329	5.6405483225
O60	-5.1681141940	2.6309185213	4.9717942075
H61	-5.2689808959	3.6289853904	5.0142178510
H62	-5.5608494620	1.4177347478	6.3723642572
O63	-5.4788535605	5.2348284496	5.1940489879
H64	-4.8276811491	5.4616612071	4.4629100705
H65	-6.3377609278	5.4433339927	4.8047793593
O66	-4.0184104058	5.5163085213	3.0870325693
H67	-3.9071734091	4.6087464582	2.7460964024
H68	-3.1088455019	5.8585755016	2.9891085726
P69	-6.0607455560	-0.4727491591	3.5155568100
O70	-4.9728447961	0.2582726098	2.5822936517
O71	-5.2492238697	-0.9616838945	4.8125789429
O72	-6.7965804727	-1.5416978871	2.8068138310
O73	-7.0055208386	0.7295072116	4.0562475763
H74	-6.4770599829	1.5155956779	4.3147079258
H75	-4.6031391373	1.0625337115	3.0299800983

Reactant state of I2M from Ru^{IV}=O species

E (B3LYP-D3/LACV3P**++ 2f(Ru))(a.u.) = -4973.51422

ZPE (kcal mol^{-1}) = 513.688

G_{solv} (kcal mol^{-1}) = -46.178

ΔH_{298} (kcal mol^{-1}) = 42.974

ΔS_{298} (cal K^{-1} mol^{-1}) = 362.931

Cartesian coordinates

Atom	x	y	z
C1	1.8446051701	1.7663758173	1.8603867363
C2	0.6587824133	2.1262661459	1.2194654676
C3	-0.4111894673	2.4881514580	2.0333153986
N4	-0.3643489785	2.5073175331	3.3574914681
C5	0.7716082303	2.1636364471	3.9783170133
C6	1.9090153660	1.7840130080	3.2542239861
H7	2.7120292290	1.4626193089	1.2814432513
H8	0.5400793650	2.1149156541	0.1423072102
H9	2.8188422578	1.4851827182	3.7630582524
Ru10	-2.8851086766	2.1785619512	4.5131472709
C11	0.2946478970	2.2597811313	8.1918542513
C12	1.5866547092	2.1092836823	7.6954244872
C13	1.7920646378	2.0830658352	6.3149733206
C14	0.6898151895	2.1998338519	5.4605064498
N15	-0.5540776112	2.3497028450	5.9447164648
C16	-0.7325031690	2.3813354057	7.2572963384
H17	0.0648709200	2.2652767679	9.2510777467
H18	2.4292950735	2.0093345634	8.3735581514
H19	2.7939437535	1.9752764844	5.9159407346
O20	-2.9455305295	2.2131471180	2.3524910770
C21	-3.6682482937	6.8822671310	5.5569870181
C22	-2.5095990617	6.4933883911	4.8751297306
C23	-2.3297852403	5.1668914710	4.5134909914
N24	-3.2451582111	4.2269212624	4.8040456168
C25	-4.3739357347	4.5804492957	5.4518437924
C26	-4.6163982207	5.8907482850	5.8285272381
H27	-1.7418043769	7.2185143133	4.6223943280
H28	-1.4550698074	4.8362735208	3.9695755964
H29	-5.0688582355	3.7865190344	5.6849667854
H30	-5.5357424981	6.1024977789	6.3635257476
C31	-2.9344440994	-2.7084568140	4.5331782739
C32	-2.3402339979	-2.0119341137	5.5906363952
C33	-2.1378139517	-0.6433532837	5.4920754886

N34	-2.4664038368	0.0565494278	4.3894048418
C35	-2.9471719020	-0.6156644625	3.3249005355
C36	-3.1817102149	-1.9813893090	3.3615763815
H37	-2.1047291218	-2.5160014114	6.5227823158
H38	-1.7766188010	-0.0697706488	6.3340949293
H39	-3.1852603910	-0.0111663575	2.4582639512
H40	-3.6129378374	-2.4696934682	2.4923122907
C41	-3.8693688366	8.3023544148	6.0172557554
H42	-3.6319424741	8.3872037414	7.0848560273
H43	-3.2240932265	8.9991565769	5.4745177633
H44	-4.9093051893	8.6185160908	5.8898033296
C45	-3.3670953125	-4.1417123361	4.6634259604
H46	-2.8623723606	-4.6458374609	5.4919508325
H47	-4.4475319949	-4.1708027795	4.8564443923
H48	-3.1825994141	-4.7047905773	3.7426998703
S49	-2.4572397459	2.5933220552	7.7606933567
O50	-3.1788674027	1.8926335542	6.6041563273
O51	-4.5913315448	1.9186819598	4.2859193453
O52	-2.6079255020	1.8085111248	8.9981629506
O53	-2.6962864540	4.0395112959	7.8286018683
S54	-2.0297426131	2.9856001250	1.3973547405
O55	-2.1468724638	2.4461394622	0.0332942026
O56	-2.1050562064	4.4430091562	1.5799333430
C57	-11.1229277976	-2.7100868125	4.5626869675
C58	-9.9308505340	-3.3943137084	4.8047386202
C59	-8.9326627828	-2.7002504792	5.4830681567
N60	-9.0526402848	-1.4482558093	5.9004574304
C61	-10.1956390911	-0.7876965862	5.6729568249
C62	-11.2657773563	-1.3923048947	5.0000964697
H63	-11.9339639232	-3.1942458870	4.0263019339
H64	-9.7537999284	-4.4112799645	4.4745418512
H65	-12.1784898201	-0.8430754522	4.7976396124
Ru66	-6.5748063104	-0.1678920326	6.0930431730
C67	-9.9467528760	3.2082606693	7.0700502365
C68	-11.2033531153	2.7337983056	6.7033107907
C69	-11.3351121359	1.4204260707	6.2485447092
C70	-10.1950334478	0.6133049958	6.1638332282
N71	-8.9862577489	1.0735553462	6.5256222726
C72	-8.8781339016	2.3196321260	6.9624888155
H73	-9.7732834090	4.2235857301	7.4074864811
H74	-12.0749307624	3.3790197125	6.7655685915
H75	-12.3088866400	1.0346499575	5.9683371979
O76	-6.4034690125	-2.2395790859	5.5095513553
C77	-6.0501454188	-0.5342220901	10.9341594200
C78	-7.1602836952	-1.1062166726	10.3012706155
C79	-7.2719164642	-1.0620964383	8.9195703850
N80	-6.3322743094	-0.4745592237	8.1574500713
C81	-5.2513013534	0.0777011191	8.7456214514
C82	-5.0780707682	0.0540261809	10.1191182120
H83	-7.9419924211	-1.5897442067	10.8793931385
H84	-8.1037314413	-1.5140771788	8.3960366376
H85	-4.5364719529	0.5606684700	8.0944658656
H86	-4.1939113438	0.5317237429	10.5274645622
C87	-6.2850711908	1.2640001705	1.4296766312
C88	-6.9813123824	2.0454879311	2.3576349549
C89	-7.2422921601	1.5480510797	3.6258989939
N90	-6.8753304910	0.3086473648	4.0033763878
C91	-6.2887907230	-0.4910579031	3.0907732992
C92	-5.9914594878	-0.0517282973	1.8103693890
H93	-7.2537408560	3.0700484821	2.1242537180
H94	-7.6852558865	2.1701316149	4.3911564837
H95	-6.0204304879	-1.4807650772	3.4392146239
H96	-5.4772546170	-0.7200974932	1.1255497037
C97	-5.9246720043	-0.5148554076	12.4352401163

H98	-6.2399721118	0.4609215664	12.8249981195
H99	-6.5500575688	-1.2800850763	12.9040230350
H100	-4.8880544035	-0.6704180354	12.7496542895
C101	-5.7978723532	1.8198210471	0.1212597253
H102	-5.9047135194	1.0931078278	-0.6907889829
H103	-6.3262860475	2.7363347711	-0.1543239812
H104	-4.7300407240	2.0579036295	0.2105207640
S105	-7.1918613924	2.7988923834	7.4086562070
O106	-6.3845414486	1.9268109751	6.4405086310
O107	-4.8478462507	-0.2470088719	5.8844721788
O108	-7.0725259847	4.2175261073	7.0302399909
O109	-7.0148277627	2.4473194958	8.8221482594
S110	-7.3181418126	-3.4073223171	5.8944558586
O111	-7.0942113578	-4.5461186764	4.9900545675
O112	-7.3465371447	-3.6608081986	7.3428906782

Transition state of I2M from Ru^{IV}=O species

E (B3LYP-D3/LACV3P**++ 2f(Ru))(a.u.) = -4973.49797

ZPE (kcal mol^{-1}) = 511.794

G_{solv} (kcal mol^{-1}) = -47.084

ΔH_{298} (kcal mol^{-1}) = 42.528

ΔS_{298} (cal K^{-1} mol^{-1}) = 365.853

Cartesian coordinates

Atom	x	y	z
C1	2.1217330000000	2.0970760000000	1.6079950000000
C2	0.9238380000000	2.4780440000000	1.0064780000000
C3	-0.1493330000000	2.7496840000000	1.8514210000000
N4	-0.0989600000000	2.6517220000000	3.1733450000000
C5	1.0569220000000	2.2994740000000	3.7599400000000
C6	2.1968700000000	2.0159550000000	2.9983770000000
H7	2.9933570000000	1.8660530000000	1.0025670000000
H8	0.7979590000000	2.5539230000000	-0.0671670000000
H9	3.1271940000000	1.7332220000000	3.4769110000000
Ru10	-2.5435450000000	2.5394210000000	4.3343710000000
C11	0.6309240000000	2.1257540000000	7.9798540000000
C12	1.8605080000000	1.7458500000000	7.4431890000000
C13	2.0552000000000	1.7955990000000	6.0621790000000
C14	1.0055500000000	2.2354130000000	5.2441300000000
N15	-0.1668490000000	2.6137350000000	5.7681580000000
C16	-0.3429090000000	2.5496910000000	7.0793400000000
H17	0.4045800000000	2.0755620000000	9.0386020000000
H18	2.6586090000000	1.3958930000000	8.0918930000000
H19	2.9996970000000	1.4770820000000	5.6368360000000
O20	-2.7062300000000	2.5976820000000	2.1758440000000
C21	-3.1291520000000	7.3147070000000	5.2297240000000
C22	-1.9549760000000	6.8336670000000	4.6418380000000
C23	-1.8370740000000	5.4875990000000	4.3327770000000
N24	-2.8244510000000	4.6119190000000	4.5834230000000
C25	-3.9681760000000	5.0562190000000	5.1461810000000
C26	-4.1526890000000	6.3909620000000	5.4673300000000
H27	-1.1273180000000	7.5010790000000	4.4214830000000
H28	-0.9470750000000	5.0890060000000	3.8647180000000
H29	-4.7322410000000	4.3158660000000	5.3436120000000
H30	-5.0955080000000	6.6779500000000	5.9217900000000
C31	-3.0489580000000	-2.3398520000000	4.2273520000000
C32	-2.5337140000000	-1.7047690000000	5.3629440000000
C33	-2.2030010000000	-0.3585690000000	5.3175340000000
N34	-2.3413460000000	0.3802180000000	4.1998150000000
C35	-2.7813210000000	-0.2301420000000	3.0803490000000
C36	-3.1305370000000	-1.5714420000000	3.0594130000000
H37	-2.4377610000000	-2.2430710000000	6.3008280000000
H38	-1.8834460000000	0.1738150000000	6.2035740000000
H39	-2.8891150000000	0.4066830000000	2.2104010000000

H40	-3.5093970000000	-2.0078640000000	2.1398040000000
C41	-3.2751610000000	8.7607520000000	5.6239850000000
H42	-3.1210560000000	8.8718470000000	6.7038590000000
H43	-2.5442390000000	9.3950780000000	5.1142770000000
H44	-4.2787910000000	9.1345870000000	5.3990220000000
C45	-3.5503960000000	-3.7561350000000	4.2620700000000
H46	-3.1830660000000	-4.2952260000000	5.1394730000000
H47	-4.6472870000000	-3.7510900000000	4.2981650000000
H48	-3.2541540000000	-4.3060460000000	3.3624840000000
S49	-2.0221900000000	2.9999570000000	7.5763330000000
O50	-2.8024030000000	2.3070390000000	6.4469830000000
O51	-4.3118070000000	2.3401230000000	4.2341320000000
O52	-2.2769260000000	2.3110590000000	8.8526070000000
O53	-2.1082310000000	4.4636570000000	7.5435290000000
S54	-1.7494760000000	3.3253420000000	1.2286070000000
O55	-1.8802460000000	2.8016550000000	-0.1421780000000
O56	-1.7508550000000	4.7840540000000	1.4105570000000
C57	-11.0884230000000	-1.7365710000000	3.8560430000000
C58	-9.8767400000000	-2.3740960000000	4.1145290000000
C59	-8.8653370000000	-1.6031100000000	4.6812660000000
N60	-8.9851820000000	-0.3131530000000	4.9685410000000
C61	-10.1562990000000	0.3034380000000	4.7367520000000
C62	-11.2378480000000	-0.3879540000000	4.1788430000000
H63	-11.9138340000000	-2.2826250000000	3.4091820000000
H64	-9.6941990000000	-3.4167350000000	3.8828840000000
H65	-12.1803980000000	0.1142470000000	3.9955200000000
Ru66	-6.6136020000000	0.9166780000000	5.3473220000000
C67	-9.9611280000000	4.3995740000000	5.8352200000000
C68	-11.1385070000000	3.9514410000000	5.2371710000000
C69	-11.2584450000000	2.6113720000000	4.8658630000000
C70	-10.1875380000000	1.7413900000000	5.1125800000000
N71	-9.0670370000000	2.1761130000000	5.7023610000000
C72	-8.9603570000000	3.4530350000000	6.0369470000000
H73	-9.7920640000000	5.4337060000000	6.1119380000000
H74	-11.9530860000000	4.6432270000000	5.0432330000000
H75	-12.1604120000000	2.2627000000000	4.3755480000000
O76	-6.3260180000000	-1.1513360000000	4.7683080000000
C77	-6.2943010000000	0.4172520000000	10.2052610000000
C78	-7.4175810000000	-0.0484700000000	9.5135460000000
C79	-7.4636660000000	0.0337330000000	8.1302140000000
N80	-6.4498370000000	0.5559290000000	7.4182160000000
C81	-5.3550900000000	1.0069400000000	8.0666730000000
C82	-5.2434580000000	0.9408240000000	9.4453760000000
H83	-8.2616090000000	-0.4760740000000	10.0462400000000
H84	-8.3112950000000	-0.3307970000000	7.5653460000000
H85	-4.5705880000000	1.4343800000000	7.4563580000000
H86	-4.3368830000000	1.3262470000000	9.9008770000000
C87	-5.8764260000000	2.3206600000000	0.7021780000000
C88	-6.5047000000000	3.1781760000000	1.6122360000000
C89	-6.8919840000000	2.7078060000000	2.8584730000000
N90	-6.7024570000000	1.4288280000000	3.2370020000000
C91	-6.1471310000000	0.5754770000000	2.3517250000000
C92	-5.7364520000000	0.9827250000000	1.0920010000000
H93	-6.6483270000000	4.2275510000000	1.3735810000000
H94	-7.3027570000000	3.3705920000000	3.6090280000000
H95	-6.0004290000000	-0.4378430000000	2.7064730000000
H96	-5.2656160000000	0.2637410000000	0.4279870000000
C97	-6.2292350000000	0.3917530000000	11.7096440000000
H98	-6.4421910000000	1.3896640000000	12.1118140000000
H99	-6.9589940000000	-0.3031710000000	12.1340870000000
H100	-5.2319760000000	0.1069740000000	12.0590100000000
C101	-5.3243320000000	2.8092500000000	-0.6073810000000
H102	-5.5663450000000	2.1191750000000	-1.4227720000000
H103	-5.7053810000000	3.8014010000000	-0.8636360000000

H104	-4.2307680000000	2.8682660000000	-0.5416470000000
S105	-7.3329880000000	3.8582550000000	6.7140060000000
O106	-6.4605670000000	3.0141960000000	5.7699540000000
O107	-4.8349870000000	0.9439020000000	5.2425220000000
O108	-7.1161860000000	5.2897450000000	6.4447170000000
O109	-7.3132540000000	3.3997090000000	8.1070630000000
S110	-7.2607260000000	-2.3032870000000	5.1452320000000
O111	-7.0352300000000	-3.4629320000000	4.2653920000000
O112	-7.3310280000000	-2.5411940000000	6.5941500000000

Product state of I2M from Ru^{IV}=O species

E (B3LYP-D3/LACV3P**++ 2f(Ru))(a.u.) = -4973.512695

ZPE (kcal mol^{-1}) = 515.219

G_{solv} (kcal mol^{-1}) = -45.223

ΔH_{298} (kcal mol^{-1}) = 42.833

ΔS_{298} (cal K^{-1} mol^{-1}) = 370.299

Cartesian coordinates

Atom	x	y	z
C1	2.1733250000000	2.2768240000000	1.5662930000000
C2	0.9513940000000	2.6105640000000	0.9877560000000
C3	-0.1268700000000	2.8097040000000	1.8472850000000
N4	-0.0575320000000	2.6760030000000	3.1663920000000
C5	1.1244280000000	2.3721860000000	3.7332250000000
C6	2.2686490000000	2.1695870000000	2.9536820000000
H7	3.0485220000000	2.1073950000000	0.9461510000000
H8	0.8110230000000	2.7074030000000	-0.0823840000000
H9	3.2193640000000	1.9359860000000	3.4192000000000
Ru10	-2.4275200000000	2.5838150000000	4.3725570000000
C11	0.7771940000000	2.1026230000000	7.9542220000000
C12	1.9852040000000	1.7040880000000	7.3824940000000
C13	2.1535960000000	1.7824490000000	5.9997960000000
C14	1.1023090000000	2.2768100000000	5.2157640000000
N15	-0.0457390000000	2.6770410000000	5.7744390000000
C16	-0.2017480000000	2.5764700000000	7.0863510000000
H17	0.5697460000000	2.0280860000000	9.0152590000000
H18	2.7842870000000	1.3125010000000	8.0049400000000
H19	3.0748580000000	1.4382550000000	5.5447780000000
O20	-2.6950980000000	2.6813680000000	2.2085360000000
C21	-3.0100890000000	7.4076440000000	5.1110620000000
C22	-1.8391310000000	6.9023570000000	4.5364840000000
C23	-1.7160480000000	5.5424990000000	4.2969740000000
N24	-2.6933100000000	4.6722200000000	4.6033210000000
C25	-3.8310150000000	5.1420910000000	5.1573300000000
C26	-4.0225470000000	6.4907740000000	5.4110890000000
H27	-1.0195720000000	7.5622090000000	4.2681360000000
H28	-0.8278180000000	5.1271430000000	3.8391310000000
H29	-4.5955650000000	4.4139490000000	5.3892730000000
H30	-4.9656290000000	6.7957200000000	5.8537120000000
C31	-3.0728390000000	-2.3052480000000	4.1223700000000
C32	-2.5848480000000	-1.7076390000000	5.2900340000000
C33	-2.2340240000000	-0.3651700000000	5.2921560000000
N34	-2.3284980000000	0.4062700000000	4.1913980000000
C35	-2.7537650000000	-0.1657980000000	3.0450850000000
C36	-3.1221680000000	-1.5006190000000	2.9774270000000
H37	-2.5195070000000	-2.2740400000000	6.2140670000000
H38	-1.9288010000000	0.1404430000000	6.1997430000000
H39	-2.8345110000000	0.4959060000000	2.1906240000000
H40	-3.4880880000000	-1.9030990000000	2.0372860000000
C41	-3.1646680000000	8.8728210000000	5.4230050000000
H42	-2.9693060000000	9.0528240000000	6.4866180000000
H43	-2.4655290000000	9.4851320000000	4.8460700000000
H44	-4.1826250000000	9.2170620000000	5.2172640000000
C45	-3.5793790000000	-3.7201810000000	4.1000190000000

H46	-3.2443230000000	-4.2846710000000	4.9746210000000
H47	-4.6766730000000	-3.7102760000000	4.0969810000000
H48	-3.2515640000000	-4.2455350000000	3.1965810000000
S49	-1.8704890000000	3.0305350000000	7.6159400000000
O50	-2.6601370000000	2.3187450000000	6.4998350000000
O51	-4.2901630000000	2.4151260000000	4.3507020000000
O52	-2.1120890000000	2.3549660000000	8.9012230000000
O53	-1.9617490000000	4.4931440000000	7.5627220000000
S54	-1.7401210000000	3.3591590000000	1.2254020000000
O55	-1.8779110000000	2.7776570000000	-0.1215560000000
O56	-1.7295890000000	4.8249550000000	1.3386940000000
C57	-11.0534920000000	-1.4630180000000	3.7300290000000
C58	-9.8271860000000	-2.0830960000000	3.9568520000000
C59	-8.7995690000000	-1.2941220000000	4.4692260000000
N60	-8.9176090000000	0.0040720000000	4.7250620000000
C61	-10.1068750000000	0.6036120000000	4.5288860000000
C62	-11.2030610000000	-0.1100290000000	4.0327270000000
H63	-11.8906530000000	-2.0269020000000	3.3294030000000
H64	-9.6459120000000	-3.1298420000000	3.7435310000000
H65	-12.1594590000000	0.3799410000000	3.8902740000000
Ru66	-6.5996780000000	1.2152830000000	5.1475770000000
C67	-9.9381900000000	4.7121620000000	5.5734520000000
C68	-11.0878970000000	4.2629020000000	4.9233180000000
C69	-11.1983880000000	2.9192020000000	4.5634030000000
C70	-10.1503800000000	2.0452670000000	4.8843460000000
N71	-9.0622720000000	2.4791140000000	5.5289040000000
C72	-8.9570210000000	3.7618870000000	5.8402130000000
H73	-9.7739090000000	5.7496340000000	5.8400110000000
H74	-11.8840270000000	4.9587960000000	4.6749110000000
H75	-12.0694990000000	2.5715670000000	4.0196300000000
O76	-6.2456060000000	-0.8520450000000	4.5983720000000
C77	-6.3239550000000	0.5489250000000	10.0057200000000
C78	-7.4542800000000	0.1415310000000	9.2885880000000
C79	-7.4913750000000	0.2872560000000	7.9101660000000
N80	-6.4638510000000	0.8164090000000	7.2223800000000
C81	-5.3636570000000	1.2108020000000	7.8978580000000
C82	-5.2590080000000	1.0823840000000	9.2731780000000
H83	-8.3100320000000	-0.2916070000000	9.7978060000000
H84	-8.3471610000000	-0.0288810000000	7.3285450000000
H85	-4.5591410000000	1.6364860000000	7.3139510000000
H86	-4.3433890000000	1.4219190000000	9.7482000000000
C87	-5.7330970000000	2.4366520000000	0.4263780000000
C88	-6.1945620000000	3.3825550000000	1.3483980000000
C89	-6.5885980000000	2.9838900000000	2.6171050000000
N90	-6.5660860000000	1.6954160000000	3.0108680000000
C91	-6.1586590000000	0.7656710000000	2.1215540000000
C92	-5.7489870000000	1.0986200000000	0.8385420000000
H93	-6.2051820000000	4.4390990000000	1.0982040000000
H94	-6.8779260000000	3.7030100000000	3.3737610000000
H95	-6.1295920000000	-0.2540150000000	2.4863050000000
H96	-5.4067490000000	0.3135820000000	0.1704320000000
C97	-6.2654130000000	0.4500060000000	11.5074250000000
H98	-6.4779300000000	1.4275250000000	11.9574900000000
H99	-6.9987110000000	-0.2633370000000	11.8942200000000
H100	-5.2701170000000	0.1460170000000	11.8458440000000
C101	-5.1835640000000	2.8368590000000	-0.9142780000000
H102	-5.4516580000000	2.1119120000000	-1.6900360000000
H103	-5.5408350000000	3.8250170000000	-1.2180060000000
H104	-4.0883400000000	2.8747770000000	-0.8549640000000
S105	-7.3416030000000	4.1560740000000	6.5501110000000
O106	-6.4680900000000	3.3352290000000	5.5824660000000
O107	-4.7312860000000	1.3197900000000	5.1027980000000
O108	-7.1161080000000	5.5935980000000	6.3270400000000
O109	-7.3304770000000	3.6517630000000	7.9272890000000

S110	-7.1940070000000	-2.0101290000000	4.9202310000000
O111	-6.9691260000000	-3.1357280000000	3.9961380000000
O112	-7.2827220000000	-2.3077780000000	6.3565490000000

References

1) Xie, Y. et al. Water Oxidation by Ruthenium Complexes Incorporating Multifunctional Bipyridyl Diphosphonate Ligands. *Angew. Chem. Int. Ed.* **55**, 8067-8071 (2016).

2) Matheu, R. et al. Intramolecular Proton Transfer Boosts Water Oxidation Catalyzed by a Ru Complex. *J. Am. Chem. Soc.* **137**, 10786-10795 (2015).

3) Matheu, R. et al. The Role of Seven-Coordination in Ru-Catalyzed Water Oxidation. *ACS Catal.* **8**, 2039-2048 (2018).

4) Shatskiy, A. et al. Electrochemically Driven Water Oxidation by a Highly Active Ruthenium-Based Catalyst. *Chemsuschem* ***12***, 2251-2262 (2019).
